# USP7 regulates the ERK1/2 signaling pathway through deubiquitinating Raf-1 in lung adenocarcinoma

**DOI:** 10.1038/s41419-022-05136-6

**Published:** 2022-08-10

**Authors:** Hong-Beom Park, Sohyun Hwang, Kwang-Hyun Baek

**Affiliations:** 1 Department of Biomedical Science, CHA University, Seongnam-Si, Gyeonggi-Do 13488 Republic of Korea; 2 Department of Pathology, Center for Cancer Precision Medicine, CHA Bundang Medical Center, CHA University School of Medicine, Seongnam-si, Gyeonggi-Do 13496 Republic of Korea

**Keywords:** Ubiquitylation, Non-small-cell lung cancer, Protein-protein interaction networks

## Abstract

Ubiquitin-specific protease 7 (USP7) is one of the deubiquitinating enzymes (DUBs) in the ubiquitin-specific protease (USP) family. It is a key regulator of numerous cellular functions including immune response, cell cycle, DNA damage and repair, epigenetics, and several signaling pathways. USP7 acts by removing ubiquitin from the substrate proteins. USP7 also binds to a specific binding motif of substrate proteins having the [P/A/E]-X-X-S or K-X-X-X-K protein sequences. To date, numerous substrate proteins of USP7 have been identified, but no studies have been conducted using the binding motif that USP7 binds. In the current study, we analyzed putative substrate proteins of USP7 through the [P/A/E]-X-X-S and K-X-X-X-K binding motifs using bioinformatics tools, and confirmed that Raf-1 is one of the substrates for USP7. USP7 binds to the Pro-Val-Asp-Ser (PVDS) motif of the conserved region 2 (CR2) which contains phosphorylation sites of Raf-1 and decreased M1-, K6-, K11-, K27-, K33-, and K48-linked polyubiquitination of Raf-1. We further identified that the DUB activity of USP7 decreases the threonine phosphorylation level of Raf-1 and inhibits signaling transduction through Raf activation. This regulatory mechanism inhibits the activation of the ERK1/2 signaling pathway, thereby inhibiting the G2/M transition and the cell proliferation of lung adenocarcinoma cells. In summary, our results indicate that USP7 deubiquitinates Raf-1 and is a new regulator of the ERK1/2 signaling pathway in lung adenocarcinoma.

## Introduction

The mitogen-activated protein kinase (MAPK) cascade is an important signaling pathway that plays a critical role in cell proliferation, metabolism, differentiation, DNA repair, and apoptosis [1–4]. The activation of MAPK kinase (MAPKK) by extracellular or intracellular responses induces phosphorylation and activation of MAPK. The phosphorylated canonical MAPKs, such as extracellular signal regulated kinase 1/2 (ERK1/2), c-Jun N-terminal kinase (JNK), p38 MAPK, and extracellular signal regulated kinase 5 (ERK5), regulate the expressions of numerous target genes that modulate diverse cellular processes [4, 5]. Among the MAPK cascade pathways, the ERK1/2 signaling pathway is the most studied and critical signaling pathway involved in regulating apoptosis, differentiation, and cell cycle. Moreover, dysregulation or mutation of the ERK1/2 signaling factors is a cause for drug resistance and varied cancers, including lung cancer [6, 7]. The binding of an epidermal growth factor (EGF) and EGF receptor (EGFR) at the cell surface initiates activation of the ERK1/2 signaling pathway. Tyrosine residues of the EGFR bound to EGF are phosphorylated. Thereafter, the growth factor receptor-bound protein 2 (GRB2) generates the GRB2-Son of sevenless (SOS) complex to bind with tyrosine-phosphorylated residues of EGFR. Activated SOS increases the guanosine triphosphate (GTP)-bound active forms of the Rat sarcoma virus (Ras) through the exchange of guanosine diphosphate (GDP) to GTP [8, 9]. The GTP-bound active form of Ras rapidly recruits accelerated fibrosarcoma (Raf) kinases (A-Raf, B-Raf, and C-Raf) to the plasma membrane and Raf activates MEK1/2 as a MAPKK kinase (MAPKKK). In turn, the phosphorylated MEK also acts as a MAPKK and induces activation and translocation of ERK1/2 to the nucleus [10].

Among the three Raf kinase family members that are conserved and ubiquitous serine/threonine protein kinases, *Raf-1* (also called C-Raf) was first discovered as a retroviral oncogene [11]. All Raf kinase families have three conserved regions (CR): CR1, CR2, and CR3 [7]. The CR1 domain contains a Ras binding domain and recruits to the plasma membrane, the CR2 domain contains phosphorylation sites that activate or inhibit the activity of Raf-1, and the CR3 domain contains the Raf kinase domain activated by phosphorylation of Raf-1 [12]. In the resting state, inactive Raf-1 is phosphorylated on Ser259 of the N-terminal region and is maintained by 14-3-3 protein binding that prevents interaction between Raf-1 and Ras-GTP. Conversely, the binding with 14-3-3 following phosphorylation of Ser621 of the C-terminal region of Raf-1 is essential for the activation of Raf-1 [13]. Dephosphorylation of Ser259 by protein phosphatase 2 A (PP2A) exposes the CR1 (the Ras binding domain) and initiates the activation of Raf-1. Furthermore, phosphorylation of Ser471, Thr491, Ser494, Ser497, Ser499, and especially Ser338, is important for complete activation of Raf-1 and signal transduction to MEK1/2 [12, 14].

Ubiquitination is a post-translational modification (PTM) and has critical functions in regulating DNA damage, autophagy, protein stability, and cell signaling activation. Ubiquitination proceeds through three enzymes: ubiquitin-activating enzyme (E1 enzyme), ubiquitin-conjugating enzyme (E2 enzyme), and ubiquitin ligase (E3 ligase). The E1 enzyme activates ubiquitin using adenosine triphosphate (ATP) and the activated ubiquitin is transferred to the E2 enzyme, after which the E3 ligases usually attach the ubiquitin to the lysine of the target proteins. The attached ubiquitin constitutes either monoubiquitination (where one ubiquitin binds to a substrate) or polyubiquitination (which forms a ubiquitin chain by binding to one or more of the seven lysine sites (K6, K11, K27, K29, K33, K48, and K63) or methionine 1 (M1) site of ubiquitin). The process in which ubiquitin is detached in the reverse process of ubiquitination is achieved by deubiquitinating enzymes (DUBs).

Ubiquitin-specific protease (USP7), also known as herpesvirus-associated ubiquitin-specific protease (HAUSP), is the most studied DUB belonging to the ubiquitin-specific protease (USP) family. USP7 was first found to be a binding partner of the herpes simplex virus type 1 immediate-early protein (Vmw110) of the herpes simplex virus type 1 (HSV-1) regulatory protein. Moreover, USP7 regulates numerous proteins that function in immune response, tumor generation, tumor suppression, epigenetics, DNA damage, and several signaling pathways. USP7 is typically known to function as a tumor suppressor or an oncogene by regulating the stability of the p53-mouse double minute (MDM) 2 axis. Recently, the roles of USP7 through deubiquitination of substrate proteins such as Yki, Axin, and tripartite motif containing 27 (TRIM27) have been revealed in several signaling pathways [15–17]. In addition, USP7 is known to have two binding pockets, one of which is the tumor necrosis factor receptor associated factor (TRAF) domain in the N-terminal region of USP7, and the other is the ubiquitin-like structures (UBL) domain of the C-terminal region. The TRAF domain of USP7 functions to bind with the [P/A/E]-X-X-S motif of diverse substrates such as p53, MDM2, MDM4, enzyme pyruvate kinase M2 (PKM2), Epstein-Barr nuclear antigen 1 (EBNA1), minichromosome maintenance complex binding protein (MCM-BP), F-box protein 38 (FBXO38), tripeptidyl-peptidase 1 (TPP1), and ubiquitin-conjugating enzyme E2 E1 (UbE2E1) [18–24]. Also, the UBL domain binds to proteins having the K-X-X-X-K sequence, such as guanine monophosphate synthase (GMPS), DNA methyltransferase 1 (DNMT1), ubiquitin like with PHD and ring finger domains 1 (UHRF1), ring finger protein 169 (RNF169), and HSV-1 [18, 25]. Therefore, proteins having the [P/A/E]-X-X-S or K-X-X-X-K sequence are expected to bind with the TRAF domain or UBL domain of USP7, respectively.

Lung cancer is the most frequently diagnosed cancer and accounts for a large percentage of cancer-related deaths in the world [26, 27]. The two most common types of lung cancer are lung adenocarcinoma (LUAD) and lung squamous cell carcinoma (LUSC), both of which are classified as non-small-cell lung cancers (NSCLC), which account for 85% of lung cancers. Both LUAD and LUSC exhibit different biological mechanisms [26, 28].

For the first time in 2006, the *USP7* expression in lung cancer was found to be associated with lung cancer tumorigenesis, and reduction of the mRNA level of *USP7* was determined to be related to poor survival via the p53-dependent pathway [29]. Furthermore, altered macrophage reprogramming by USP7 inhibition promotes M1 polarization of tumor-related macrophages and inhibits lung cancer cell proliferation [30]. However, the cellular functions of USP7 in lung cancer remain controversial. In LUSC and large cell carcinomas, inhibition of USP7 promotes cancer cell apoptosis through the MDM2-p53 axis [31]. Also, in LUAD cell lines, inactivation of USP7 induces sensitivity of paclitaxel and docetaxel through degradation of Ki-67 [32]. However, a recent study reported that inhibition of USP7 reversely induces cell proliferation regulating SMAD3 autoregulation regardless of the p53 axis in p53-deficient lung cancer [33].

In the current study, proteins having the [P/A/E]-X-X-S and K-X-X-X-K motifs were identified by the bioinformatics tool. Of these, putative substrate proteins expected to bind with USP7 were selected. Using functional annotation analysis, we confirmed that the MAPK signaling pathway is correlated with putative USP7 substrate proteins, and found that Raf-1 is a novel binding partner of USP7. In addition, USP7 deubiquitinates M1-, K6-, K11-, K27-, K33-, and K48-linked polyubiquitin chains of Raf-1, and inhibits the ERK1/2 signaling pathway, disturbing the threonine phosphorylation of Raf-1. Collectively, we found that USP7 inhibits the ERK1/2 signaling pathway by regulating the activity of Raf-1 and suppresses LUAD cell viability in a manner independent of the p53/MDM2 axis.

## Materials and methods

### Cell culture and transfection

HEK293T, HeLa, and H1299 cells were grown in Dulbecco’s Modified Eagle’s Medium (Cat #12800-017, DMEM, Gibco, Grand Island, NY, USA) containing 10% fetal bovine serum (FBS, Gibco, Grand Island, NY, USA) and 1% antibiotic-antimycotic reagent (Cat #15240062, Gibco, Grand Island, NY, USA). A549 cells were grown in Roswell Park Memorial Institute (RPMI)-1640 medium (Cat #11875-093, Gibco BRL, Rockville, MD, USA). Transfections were performed with 150 mM NaCl and polyethyleneimine (Cat #23966, PEI, Polysciences, Inc., Warrington, PA, USA) and Lipofectamine 2000 Reagent (Cat #11668-019, Invitrogen, Waltham, MA, USA). The cells were grown in a 5% CO_2_ incubator at 37 °C.

### Bioinformatics analysis

Using the MOTIF search (https://www.genome.jp/tools/motif/MOTIF2.html), the proteins having either the [P/A/E]-X-X-S or K-X-X-X-K sequence were found. We set the pattern in the PROSITE format field to [P/A/E]-X-X-S or K-X-X-X-K, set the *Homo sapiens* (hsp) to the Kyoto Encyclopedia of Genes and Genomes (KEGG) gene, and set the maximum number to 20,000. And then, only proteins having both the [P/A/E]-X-X-S and K-X-X-X-K sequences were selected. The gene ID was changed to ENSEMBL gene ID for the database for annotation, visualization, and integrated discovery (DAVID) analysis. DAVID analysis was performed except for unknown genes or duplicated genes. Protein information of 184 genes including components in the MAPK signaling pathway was uploaded to the STRING network (https://version-11-0b.string-db.org) using Cytoscape. And then, binding between already published proteins and USP7 is shown in gray, and interactions that have not yet been discovered are shown in green.

### Western blotting and immunoprecipitation

HEK293T, HeLa, A549, and H1299 cells were lysed with lysis buffer (50 mM Tris-HCl [pH 7.5], 1 mM EDTA, 10% glycerol, 300 mM NaCl, and 1% Triton X-100). Samples were incubated in an ice-cold environment for 20 min and centrifuged at 13,000 rpm for 20 min at 4 °C, and supernatants were collected. Western blotting was performed using sodium dodecyl-sulfate polyacrylamide gel electrophoresis (SDS-PAGE) gels, and proteins were transferred to polyvinylidene fluoride (PVDF) microporous membranes (Cat #IPVH00010, Millipore, Billerica, MA, USA), which were then blocked with TTBS (20 mM Tris-HCl [pH 7.5], 0.05% Tween 20 and 150 mM NaCl) containing 5% skim milk or 5% bovine serum albumin (BSA) for 1 h and incubated overnight at 4 °C with primary antibodies. Membranes were then washed three times for 7 min each with TTBS, incubated for 2 h with the secondary antibody, and rewashed three more times in TTBS. Blots were detected using an ECL reagent solution (Cat # LF-QC0101, Young-In Frontier, Seoul, Korea).

For the immunoprecipitation study, cell lysates were incubated with an antibody at 4 °C overnight and then for 2 h with protein A/G PLUS-Agarose Beads (Cat #sc-2003, Santa Cruz Biotechnology, Santa Cruz, CA, USA). Samples were boiled in 2X SDS sample buffer for 7 min and detected by Western blotting. Ubiquitination and deubiquitination assays were performed by the ubiquitination assay kit according to the manufacturer’s manual (Cat #uBAK-100, D&P Biotech Inc., Seoul, Korea).

### Plasmid DNA and antibodies

*Raf-1* gene and deletion mutants of *Raf-1* gene were subcloned into the pCS4-Flag vector, deletion mutants of *USP7* gene were subcloned into pcDNA3.1-6-Myc vector. And Myc*-USP7* (WT), Myc*-USP7* (C223S), pGEX-4T-1 vector, pGEX-4T-1-*USP7*, and wild-type HA*-ubiquitin* (Ub) and its mutants (M1, K6, K11, K27, K29, K33, K48, and K63) were used as previously described [19, 34]. Deletion mutant constructs of *USP7* (TRAF and UBL) and *Raf-1* (Δ1, Δ2, Δ3, and Δ4) were produced by subcloning. And mutants for the binding motif of Flag*-Raf-1* (S274R, S283R, S289R, S291R, S295R, S322R, S428R, S508R, and S571R) were produced by site-directed point mutagenesis. The primers for making deletion mutants and binding motif mutants are described in Supplement Table S1.

Anti-HA (12CA5) and anti-Myc (9E10) antibodies were acquired from hybridoma cell media and Anti-Flag (Cat #M185-3L, Sigma-Aldrich, St. Louis, MO, USA), anti-β-actin (Cat #sc-4778, Santa Cruz Biotechnology, Santa Cruz, CA, USA), anti-USP7 (Cat #sc-137008, Santa Cruz Biotechnology, Santa Cruz, CA, USA), anti-p53 (Cat #sc-126, Santa Cruz Biotechnology, Santa Cruz, CA, USA), anti-p-Tyr (Cat #sc-508, Santa Cruz Biotechnology, Santa Cruz, CA, USA), anti-p-Ser (Cat #sc-81514, Santa Cruz Biotechnology, Santa Cruz, CA, USA), anti-p-Thr (Cat #sc-5267, Santa Cruz Biotechnology, Santa Cruz, CA, USA), anti-p-Raf-1 (Cat #sc-271928, Santa Cruz Biotechnology, Santa Cruz, CA, USA), anti-Raf-1 (Cat #sc-7267, Santa Cruz Biotechnology, Santa Cruz, CA, USA), anti-Raf-1 (Cat #LF-PA0195, Ab Frontier, Seoul, Korea), anti-MEK (Cat #sc-6250, Santa Cruz Biotechnology, Santa Cruz, CA, USA), anti-p-MEK (Cat #sc-81503, Santa Cruz Biotechnology, Santa Cruz, CA, USA), anti-ERK (Cat #9102 L, Cell Signaling, Danvers, MA, USA) and anti-p-ERK (Cat #4668 S, Cell Signaling, Danvers, MA, USA) antibodies were used for Western blotting, GST pull-down assay, and immunoprecipitation.

### Glutathione S-transferase (GST) pull-down assay

For protein induction, Escherichia coli BL21 cells transformed with pGEX-4T-1 vector or pGEX-4T-1-*USP7* were incubated at 20 °C overnight. LB broth (5 mL) was then added in a 15 mL conical tube. Transfected cells were induced using 1 mM isopropyl β-D-1-thiogalactopyranoside (IPTG) (Cat #V3955, Promega, Madison, WI, USA) and adjusted to an A600 of 0.4–0.5. Cells were lysed by sonication and incubated with glutathione-sepharose beads (Cat #27-4574-01, Pharmacia Biotech, Uppsala, Sweden). Purified proteins were rotated with HEK293T cell lysates overexpressing Flag-Raf-1 at 4 °C overnight, and bound proteins were analyzed by Western blotting.

### Immunocytochemistry

HeLa cells were seeded on glass coverslips, placed on a 12-well plate, washed briefly with phosphate-buffered saline (PBS), fixed with 4% formaldehyde for 15 min, blocked with PBS containing 1% BSA for 1 h at room temperature, and treated with primary antibodies (Raf-1 and USP7) in 1% BSA at 4 °C overnight. Cells were then washed with PBS, incubated with Alexa-Fluor-488-conjugated goat anti-mouse (Cat #a11001, Invitrogen, Carlsbad, CA, USA) and with Alexa-Fluor-568-conjugated goat anti-rabbit (Cat #a11011, Invitrogen, Carlsbad, CA, USA) for 1 h at room temperature in the dark, washed with PBS, and stained with DAPI (Cat #D9542, Sigma-Aldrich, St. Louis, MO, USA). Samples were visualized under a confocal microscope (Zeiss LSM880, Carl Zeiss Microscopy GmbH, Jena, Germany).

### Protein stability assay

HEK293T cells were transfected with *siUSP7* (GenePharma. Shanghai, China) or were treated with USP7 inhibitor, P22077 (Cat #HY-13865, MedChemExpress, NJ, USA). After 24 h of incubation, Cycloheximide (CHX) (100 μg/ml) (Cat #01810, Sigma-Aldrich, St. Louis, MO, USA) was treated into HEK293T cells. Samples were harvested in 6, 12, and 24 h later. And then, the protein stability was determined by Western blotting analysis.

### Annexin V staining and cell apoptosis analysis

Annexin V staining and cell apoptosis analysis was performed with a fluorescence-activated cell sorting (FACS) Calibur (BD BioScience, San Jose, CA, USA) and CellQuest analysis software. Apoptosis was determined by staining with Annexin V-FITC/propidium iodide (PI) (Cat #556547, BD BioScience, San Jose, CA, USA) double staining according to the manufacturer’s instructions. Briefly, cells (1 × 10^6^) were washed with cold PBS and then resuspended in a binding buffer (10 mM Hepes/NaOH (pH 7.4), 0.14 M NaCl, 2.5 mM CaCl_2_), and FITC Annexin V and PI solution were added. After incubation at room temperature for 15 min in the dark, Annexin V-FITC and PI-staining was analyzed by flow cytometry. Annexin V-FITC and PI double staining were regarded as late apoptotic or necrotic cells.

### Cell cycle analysis

For cell synchronization in G1 or early S phage, A549 and H1299 cells were synchronized by double thymidine block. The first thymidine block was treated with thymidine (Cat #SLBT0908, Sigma-Aldrich, St. Louis, MO, USA) with a final concentration of 2 mM for 16 h. And then, cells were washed with PBS and cultured in fresh media for 8 h followed by the second thymidine block (2 mM). The cell cycle was released after PBS washing and fresh media change. H1299 cells were harvested at 0, 4, and 8 h after the release and A549 cells were harvested at 0, 6, 12, and 18 h after the release. The cells were washed in PBS and were fixed and permeabilized in ice-cold 70% ethanol at −20 °C. The fixed cells were washed with PBS to remove ethanol. Then, the cells were stained with 0.5 mL of FxCycle™ PI/RNase staining solution (Cat #F10797, Thermo Fisher Scientific, Waltham, MA, USA) for 30 min. The stained cells were analyzed using flow cytometry.

### Cell motility assay

For the colony formation assay, A549 and H1299 cells (2 × 10^3^) transfected with pcDNA3.1–6myc vector or Myc-*USP7* were seeded onto 100 mm cell culture plates, and the medium was changed every 2–3 days. After 1–2 weeks, the colonies were washed with PBS and stained with crystal violet solution. Following washing with PBS, images were captured using a DUALED Blue/White Transilluminator (Cat #A-6020, Bioneer, Daejeon, Korea) and the number of colonies in each plate were manually counted using image J. For wound healing assay, A549 cells (2 × 10^3^) transfected with pcDNA3.1–6myc vector or Myc-*USP7* were seeded onto 6-well plates, and the cells were wounded with a 10-µl micropipette tip. Images were captured at 0, 12, 24, and 48 h, and the distances between cells were measured using Image J (National Institutes of Health, Bethesda, MD, USA).

### Statistical analysis

Densitometric analysis was performed by Image J, and the paired *t*-test and two-way ANOVA were performed by GraphPad Prism version 5 (GraphPad Software, La Jolla, CA, USA). *p*-values of *p < 0.05, **p < 0.01, ***p < 0.001 were deemed significant. All the results shown are representative data of at least three independent experiments and are presented as the mean ± standard error of the mean (SEM).

## Results

### Motif analysis for identifying new binding partners of USP7

**Fig. 1:**
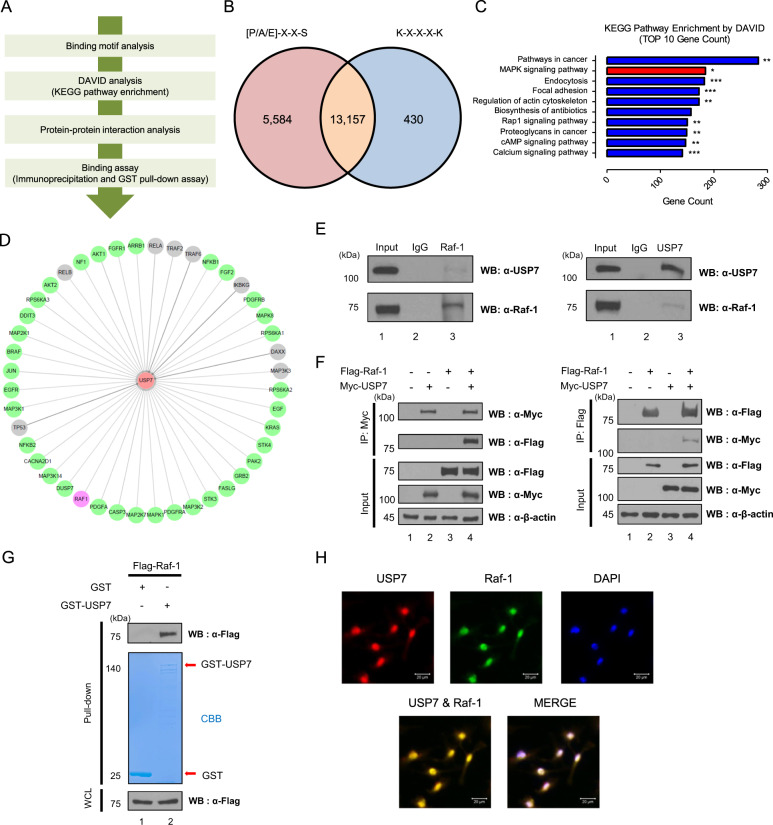
Motif analysis for identifying new binding partners of USP7. **A** Schematic overview of the workflow for identifying new binding partners of USP7 using binding motifs, DAVID, protein-protein interaction, and binding analyses. **B** The diagram shows the number of proteins having each or both [P/A/E]-X-X-S and K-X-X-X-K motifs. **C** Pathway enrichment of putative substrate proteins of USP7 was analyzed by DAVID analysis. Y-axis indicates the pathway name, and X-axis indicates gene count in the pathways, and the asterisk indicates the *p*-value (*p < 0.05, **p < 0.01, ***p < 0.001.) **D** Interaction network between USP7 and putative substrates with high interaction score and with or without the connection revealed. The color of nodes indicates the confirmation of binding between USP7 and proteins (Gray: published and green: nonpublished), and Raf-1 binding with USP7 is shown in purple. Interaction network is shown by Cytoscape. **E** Endogenous USP7 or Raf-1 was precipitated with an anti-USP7 or an anti-Raf-1 antibody in HeLa cell lysate. **F** HEK293T cells were transfected with Myc-*USP7*, and Flag-*Raf-1* and immunoprecipitations were performed by an anti-Myc or an anti-Flag antibody. **G** Purified GST- or GST-USP7 expressed in BL21 was incubated with Flag-*Raf-1*-overexpressed HEK293T cells. GST- or GST-USP7 was pull downed by GST bead and gel was stained by Coomassie Brilliant Blue. **H** Immunocytochemical analysis was performed to investigate the localization of respective USP7 and Raf-1, and co-localization of USP7 and Raf-1.

To find novel substrate proteins of USP7, we used two USP7 binding motifs: [P/A/E]-X-X-S and K-X-X-X-K. Proteins with the binding motifs are expected to interact and be deubiquitinated by USP7, and especially proteins carrying both the binding motifs are more likely to bind to USP7. Therefore, in this study, we found and analyzed proteins with both [P/A/E]-X-X-S and K-X-X-X-K binding motifs and discovered new putative substrate proteins of USP7. The overall analysis method is presented as a schematic overview (Fig. 1A).

We first analyzed the putative substrate proteins of USP7 through the MOTIF search that detects proteins having the [P/A/E]-X-X-S and K-X-X-X-K motifs. Totally, 13,157 proteins having both the [P/A/E]-X-X-S and K-X-X-X-K motifs were determined (Fig. 1B and Datasheet 1). Thereafter, to select proteins that actually bind to USP7, we performed the DAVID analysis [35]. The pathways with TOP 10 gene count were summarized by analyzing the proteins with two binding motifs through the KEGG pathway enrichment. In addition, it is previously known that substrate proteins of USP7 play an important role in signal transduction, and several signaling pathways such as Wnt/β-catenin, Hippo, NF-kB, Hedgehog, and Notch are positively or negatively regulated by USP7 [15, 16, 24, 36]. Therefore, we focused on signaling pathways with TOP 10 gene count among the KEGG pathway enrichment and found that the putative substrate proteins are significantly related to the MAPK signaling pathway as well as several other signaling pathways (Fig. 1C).

Using the gene information belonging to the MAPK signaling pathway organized by DAVID analysis, we narrowed down the putative substrate proteins of USP7 by protein-protein interaction (PPI) analysis in the STRING database [37]. We only selected the edge connected to USP7, and using the BioGRID interaction database (https://thebiogrid.org/), we exclude the proteins previously known to bind USP7 [38]. The interaction network was drawn using a Cytoscape (Fig. 1D). Among the putative substrate proteins, Raf-1 is a serine/threonine kinase essential for signal transduction from Ras to MEK1/2 in the MAPK signaling pathway. Also, in the TOP 10 gene count of KEGG pathway enrichment, Raf-1 belongs to other signaling pathways, such as focal adhesion, regulation of actin cytoskeleton, Rap-1 signaling pathway, cAMP signaling pathway, and proteolysis in cancer. In addition, we have previously elucidated the phosphorylation sites required for the activity of D-Raf and its cellular functions in the Torso (Tor) signaling pathway [39]. Therefore, we investigated whether USP7 binds several putative substrate proteins, including Raf-1.

To confirm whether putative substrate proteins bind to USP7, we performed an immunoprecipitation assay using an anti-USP7 antibody. The putative substrate proteins ME2K1 (MEK1), MAPK1/2 (ERK1/2), MAPK9 (JNK2), NFKB1, FASLG (Fas), and AKT1 do not bind to USP7 (Supplement Fig. S1A, B). However, Raf-1 was determined to bind to USP7. Reversely, endogenously expressed USP7 was precipitated by an anti-Raf-1 antibody (Fig. 1E), and binding between exogenously expressed Flag-Raf-1 also binds to Myc-USP7 (Fig. 1F). In addition, we checked for direct binding between USP7 and Raf-1 through the GST pull-down assay using purified GST-USP7 (Fig. 1G). In a previous study, we found that USP7 exists in the nucleus and the cytoplasm of HeLa cells [40]. In this study, we confirmed the co-localization of USP7 and Raf-1 in HeLa cells. Immunocytochemical analysis revealed that USP7 and Raf-1 co-localize both in the nucleus and the cytoplasm of HeLa cells (Fig. 1H). These results suggest that Raf-1 can be a novel binding partner of USP7.

### The PVDS motif belonging to the CR2 domain of Raf-1 interacts with the TRAF domain of USP7

**Fig. 2:**
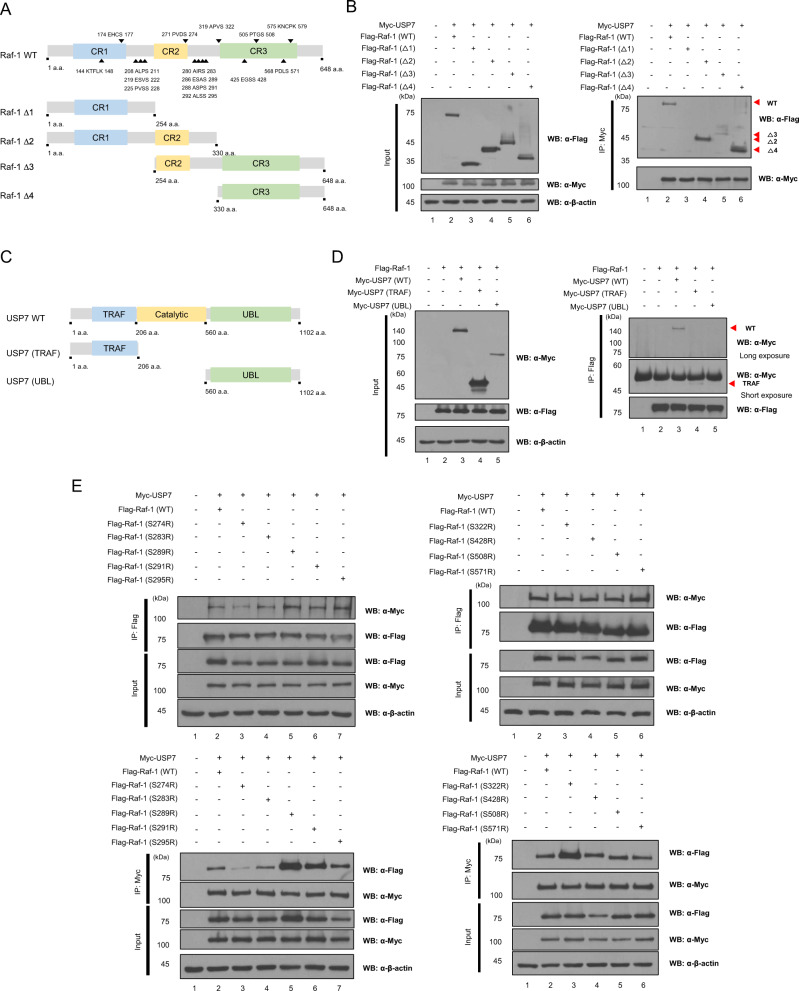
The PVSS motif belonging to the catalytic domain of Raf-1 binds to proteins containing the ubiquitin-like (Ubl) domain of USP7. **A** The deletion mutants and two binding motif sites ([P/A/E]-X-X-S and K-X-X-X-K) of Raf-1 are indicated by the schematic drawing. Raf-1 ∆1 contains only the CR1 domain and Raf-1 ∆2 contains the CR1 and CR2 domains. Raf-1 ∆3 contains the CR2 and CR3 domains, and Raf-1 ∆4 contains only the CR3 domain (Raf-1 ∆1:1 a.a.-254 a.a., Raf-1 ∆2:1 a.a.-330 a.a., Raf-1 ∆3: 254 a.a.-648 a.a., and Raf-1 ∆4: 330 a.a.-648 a.a.). **B** HEK293T cells were both transfected with Myc-*USP7* and four different Flag-Raf-1 deletion mutants. And then immunoprecipitation assay using deletion mutants of Raf-1 was performed with an anti-Myc antibody. **C** The deletion mutants of *USP7* are indicated by the schematic drawing. USP7 (TRAF) contains only the TRAF domain of USP7 and USP7 (UBL) contains only the UBL domain (USP7 (TRAF): 1 a.a.-104 a.a. and USP7 (UBL): 562 a.a.-1103 a.a.). **D** Flag-*Raf-1* and two different Myc-*USP7* deletion mutants were transfected into HEK293T cells and then immunoprecipitation assay was performed with an anti-Flag antibody. **E** The binding motif mutants of *Raf-1* (S274R, S283R, S289R, S291R, S295R, S322R, S428R, S508R, and S571R) are generated by site-directed mutagenesis and Myc-*USP7* were co-transfected into HEK293T cells. And then immunoprecipitations were performed with an anti-Myc antibody.

Through bioinformatics analysis and binding study, we found that USP7 directly binds to Raf-1. To identify the binding domain and which protein motifs of Raf-1 bind to USP7, we first generated deletion forms of *Raf-1* (Fig. 2A). Myc-*USP7* and each deletion form of Flag-*Raf-1* were transfected into HEK293T cells. Immunoprecipitation analysis revealed that Myc-USP7 binds to Raf-1 ∆2, ∆3, and ∆4 containing the CR2 and CR3 domains, but the other deletion forms containing the CR1 domain were not detected (Fig. 2B). We also determined that the TRAF and UBL domains of USP7 bind to [P/A/E]-X-X-S or K-X-X-X-K motif, respectively. Therefore, the TRAF domain and UBL domain mutants of *USP7* were generated to confirm which of the two binding motifs on Raf-1 binds to USP7 (Fig. 2C). Flag-*Raf-1* and deletion mutants of *USP7* were transfected into HEK293T cells, and immunoprecipitation analysis showed that Flag-Raf-1 binds to the TRAF domain of USP7 (Fig. 2D). These results indicate that Raf-1 binds to USP7 via the [P/A/E]-X-X-S motif.

We further investigated the [P/A/E]-X-X-S and K-X-X-X-K motifs in the CR2 and CR3 domains of Raf-1 (Fig. 2A). Nine [P/A/E]-X-X-S motifs were determined to belong to the CR2 and CR3 domains of Raf-1: PVDS (271-274), AIRS (280–283), ESAS (286–289), ASPS (288–291), ALSS (292–295), APVS (319–322), EGSS (425–428), PTGS (505–508), and PDLS (568–571). The last serine residue of these motifs was mutated to arginine for detecting the binding motif of Raf-1. Binding motif mutants of *Raf-1* and Myc-*USP7* were transfected into HEK293T cells and immunoprecipitated by an anti-Myc or an anti-Flag antibody. The binding affinity of Flag-Raf-1 (S274R) was found to be decreased, compared to the other motif mutants (Fig. 2E). These results show that the PVDS motif in the CR2 domain of Raf-1 binds to the TRAF domain of USP7.

### USP7 deubiquitinates Raf-1

**Fig. 3:**
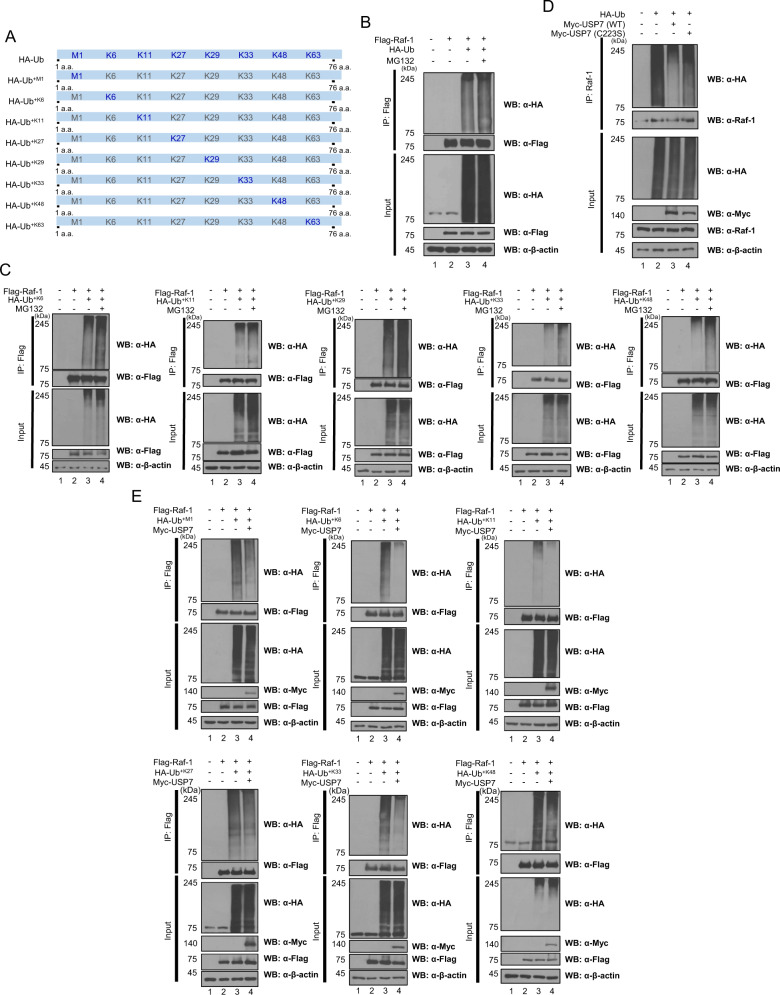
USP7 deubiquitinates M1-, K6-, K11-, K27-, K33-, and K48-linked polyubiquitin chains of Raf-1. **A** Schematic diagram of wild-type ubiquitin and its mutant constructs (M1, K6, K11, K27, K29, K33, K48, and K63). **B** Ubiquitination assay of Raf-1 was performed in HEK293T cells transfected with Flag-*Raf-1* and HA-*Ub* and treated with a proteasome inhibitor MG132. **C** HEK293T cells were transfected with Flag-Raf-1 and each HA-*Ub* mutant (K6, K11, K29, K33, and K48) and treated with a proteasome inhibitor MG132. And then ubiquitination assay of Raf-1 was performed for checking the association between the specific lysine sites of polyubiquitination and UPS. **D** Myc-*USP7* and Flag-*Raf-1* were transfected into HEK293T cells and Flag-Raf-1 was precipitated for deubiquitination assay. For checking the decrease of overall polyubiquitination of Raf-1 by DUB activity of USP7, deubiquitination assay was performed. **E** HEK293T cells were transfected with each HA-*Ub* mutant (M1, K6, K11, K27, K33, and K48) and Myc-*USP7*. A change in the polyubiquitin chains (M1, K6, K11, K27, K33, and K48) of Raf-1 by USP7 was detected through deubiquitination assay.

Raf-1 undergoes post-translational modifications including ubiquitination and phosphorylation, thereby regulating the function of Raf-1 [41]. Ubiquitination creates diverse types of ubiquitin chains on one methionine site and seven lysine sites (for example, M1, K6, K11, K27, K29, K33, K48, and K63) of ubiquitin [42]. Ubiquitination of Raf-1 has been reported, and it is known that E3 ligases such as CHIP and HUWE1 mediate ubiquitination of Raf-1 [12, 43, 44]. However, details of chain-linked polyubiquitination of Raf-1 and the regulation of Raf-1 stability by the ubiquitin-proteasome system (UPS) through polyubiquitination are not well known. Therefore, wild-type *ubiquitin* and its mutant constructs (M1, K6, K11, K27, K29, K33, K48, and K63) were generated for checking which types of polyubiquitin chains are formed for Raf-1 (Fig. 3A). Ubiquitination assay of Raf-1 was performed by immunoprecipitation of HEK293T cells transfected with wild-type *ubiquitin*, its mutants, and Flag-*Raf-1*. MG132 (a proteasome inhibitor) was also treated to verify that the stability of Raf-1 is regulated by UPS. In the ubiquitination assay using wild-type ubiquitin, the overall ubiquitination of Raf-1 is associated with UPS (Fig. 3B). Moreover, K6-, K11-, K29-, K33-, and K48-linked polyubiquitinations of Raf-1 are associated with UPS, while M1-, K27-, and K63-linked polyubiquitinations are irrelevant (Fig. 3C and Supplement Fig. S2A). These results indicate that Raf-1 undergoes polyubiquitination on one methionine site and seven lysine sites of ubiquitin, and K6-, K11-, K29-, K33-, and K48-linked polyubiquitinations of Raf-1 are related to protein degradation of Raf-1 through UPS.

The DUB activity of USP7 regulates the ubiquitination level of various proteins including MDM2, p53, nucleolin, annexin-1, Axin, PP2A, and PKM2 [16, 18, 19, 45, 46]. Depending on which polyubiquitin chain is deconjugated by the DUB activity of USP7, the stability or cellular functions of target proteins are regulated. To confirm that USP7 deubiquitinates Raf-1, and which polyubiquitin chains of Raf-1 were deconjugated by USP7, we performed deubiquitination assays. We first confirmed that polyubiquitin chains of Raf-1 were reduced in HEK293T cells transfected with Myc-*USP7* (WT) but not reduced in HEK293T cells transfected with a catalytic mutant of Myc-*USP7* (C223S) (Fig. 3D). Thereafter, the effects of USP7 on M1-, K6-, K11-, K27-, K29-, K33-, K48-, and K63-linkd polyubiquitin chains of Raf-1 were investigated using ubiquitin mutants. Among the eight polyubiquitin chain types, M1-, K6-, K11-, K27-, K33-, and K48-linked polyubiquitin chains of Raf-1 were determined to be regulated by the DUB activity of USP7, but the K29- and K63-linked polyubiquitin chains of Raf-1 were not (Fig. 3E and Supplement Fig. S2B). These results indicate that USP7 functions as a DUB targeting M1-, K6-, K11-, K27-, K33-, and K48-linked polyubiquitin chains of Raf-1.

### USP7 regulates the ERK1/2 signaling pathway by reducing the phosphorylation level of Raf-1

The ubiquitination level of target proteins not only affects the stability of the target protein but is also involved in cellular and protein functions such as gene induction, DNA damage response, protein trafficking, and protein kinase. We confirmed that K6-, K11-, K29-, K33-, and K48-linked polyubiquitinations of Raf-1 are related to proteasomal degradation mediated by UPS and USP7 deubiquitinates M1-, K6-, K11-, K27-, K33-, and K48-linked polyubiquitinations of Raf-1. Typically, M1-linked polyubiquitination is related to innate immunity, whereas K6-, K11-, and K48-linked polyubiquitinations are involved in protein degradation by UPS. Moreover, K27- and K33-linked polyubiquitinations are involved in kinase modification [47–49]. Therefore, the protein stability or kinase activity of Raf-1 could be controlled by the DUB activity of USP7. To check whether deubiquitination of Raf-1 by USP7 inhibits UPS-dependent proteasomal degradation of Raf-1, the expression of USP7 was knocked down or overexpressed using *siUSP7* or Myc-*USP7* construct, respectively. *siRNA* targeting USP7 was previously designed and used [45]. However, no change was observed in the stability of Raf-1 depending on the expression of USP7 (Supplement Fig. S3A). Also, the CHX exposure with P22077 (USP7 inhibitor) or *siUSP7* showed that the half-life of Raf-1 is not regulated by USP7 (Supplement Fig. S3B). These results indicate that the protein stability of Raf-1 is not regulated by the DUB activity of USP7.

**Fig. 4:**
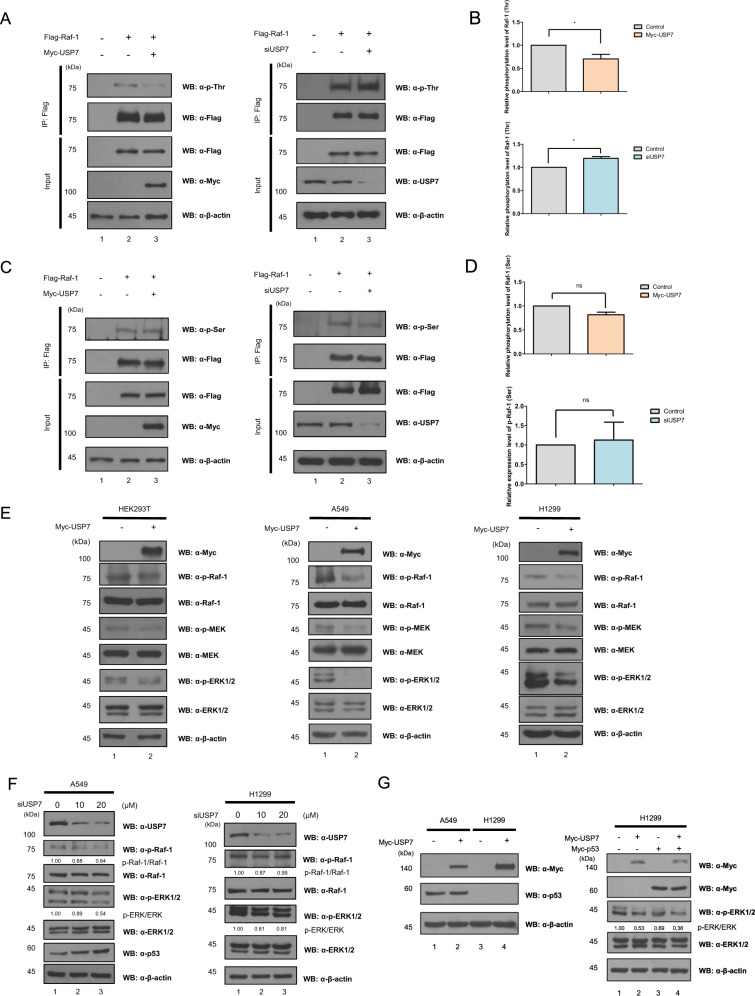
USP7 regulates the ERK1/2 signaling pathway by reducing the phosphorylation level of Raf-1. **A** Flag-*Raf-1* and Myc-*USP7* or *siUSP7* were transfected into HEK293T cells and Flag-Raf-1 was precipitated by an anti-Flag antibody. Threonine phosphorylation of Raf-1 was detected by Western blotting using an anti-p-Thr antibody. **B** Threonine phosphorylation level of Flag-Raf-1 in at least three independent experiments was calculated by a two-tailed Student’s *t*-test, *p < 0.05, *n* = 5 (left), *n* = 3 (right). **C** Flag-*Raf-1* and Myc-*USP7* or *siUSP7* were transfected into HEK293T cells and Flag-Raf-1 was precipitated by an anti-Flag antibody. Threonine phosphorylation of Raf-1 was detected by Western blotting using an anti-p-Ser antibody. **D** Serine phosphorylation level of Flag-Raf-1 in at least three independent experiments was calculated by a two-tailed Student’s *t*-test, *ns* = not significant, *n* = 3 (left), *n* = 3 (right). **E** The expression of the ERK1/2 signaling factors (Raf-1, MEK1/2, and ERK1/2) and their active forms (p-Raf-1, p-MEK1/2, and p-ERK1/2) were detected by Western blotting using indicated antibodies in HEK293T, A549, and H1299 cells. The experiment was performed at least three times and representative data were shown. **F** *siUSP7* was transfected into A549 and H1299 cells in a dose-dependent manner. The expression of USP7, p53, Raf-1, p-Raf-1, ERK1/2, and p-ERK1/2 was detected by Western blotting. **G** Myc-*USP7* was transfected into A549 and H1299 cells. The expression of Myc-USP7, and p53 was detected by Western blotting, and a partial deletion form of p53 protein in H1299 cells was also confirmed by Western blotting using an anti-p53 antibody. Myc-*USP7* was transfected with or without Myc-*p53* in H1299 cells. The expression of ERK1/2 and p-ERK1/2 was detected by Western blotting. The experiment was performed at least three times and representative data were shown.

We subsequently investigated alternations in the phosphorylation level of Raf-1 to confirm that the deubiquitination of Raf-1 is related to the kinase activity of Raf-1. Raf-1 is phosphorylated at serine, threonine, and tyrosine sites, and phosphorylation at each site activates or inhibits the activity of Raf-1. We applied phospho-specific antibodies to confirm changes in the overall phosphorylation at the serine, threonine, and tyrosine sites of Raf-1 by overexpression of USP7. Immunoprecipitation assay showed that threonine phosphorylation levels of Raf-1 were significantly decreased, while serine phosphorylation level of Raf-1 was weakly decreased with USP7 overexpression (Fig. 4A, B). Conversely, knockdown of *USP7* resulted in increased threonine phosphorylation levels of Raf-1. The serine phosphorylation level of Raf-1 by *USP7* knockdown increases but is not significant. (Fig. 4C, D). However, tyrosine phosphorylation of Raf-1 was not detected by immunoprecipitation assay (Supplement Fig. S4). These results show that USP7 suppresses threonine phosphorylation of Raf-1.

Phosphorylation of Raf-1 is not only related to activation of Raf-1 but also related to activation of the ERK1/2 signaling pathway through inducing phosphorylation of MEK1/2. Since USP7 overexpression inhibits the threonine phosphorylation of Raf-1, activation of MEK1/2 and ERK1/2 is expected to be affected by USP7. To confirm that the DUB activity of USP7 affects the ERK1/2 signaling pathway, the activated phosphorylation levels of Raf-1, MEK1/2, and ERK1/2 were analyzed in kidney and LUAD cell lines, where Raf-1 dysregulation is already known due to upregulation of Raf-1 or downregulation of Raf regulatory proteins [50, 51]. USP7 overexpression suppresses the activation of Raf-1, MEK1/2, and ERK1/2 in HEK293T, A549, and H1299 cell lines (Fig. 4E). Since USP7 interacts with only Raf-1 in the Ras-Raf-MEK-ERK cascade (Supplement Fig. S1A, B), these results indicate that USP7 inhibits the ERK1/2 signaling pathway via binding to Raf-1. Also, downregulation of USP7 was applied in A549 and H1299 cells to determine whether *USP7* knockdown showed opposite results to USP7 overexpression. Surprisingly, the *USP7* knockdown suppressed the activation of ERK1/2 along with an increase in p53 protein level in A549 cells. However, knockdown of *USP7* in H1299 cells that have a partial deletion of the p53 protein did not induce a significant decrease in ERK1/2 activation (Fig. 4F). These results indicate that the knockdown of *USP7* inhibits the ERK1/2 signaling pathway via a p53-dependent mechanism. We also examined the p53 level to determine whether USP7 overexpression regulates the ERK signaling pathway via a p53-dependent mechanism. However, USP7 overexpression does not affect the protein level of p53 in A549 cells, and inhibits the activation of ERK1/2 signaling in p53-overexpressed H1299 cells (Fig. 4G). These results suggest that the ERK1/2 signaling pathway is regulated by a different mechanism depending on the expression of USP7, and that the ERK1/2 signaling pathway is modulated independently of p53 level in USP7 overexpression, and dependently of p53 level in knockdown of *USP7*. Thus, our results indicate that USP7 overexpression inhibits the ERK1/2 signaling pathway by deubiquitination of Raf-1 and independently of p53.

### USP7 inhibits LUAD cell proliferation via modulation of the ERK1/2 signaling pathway

**Fig. 5:**
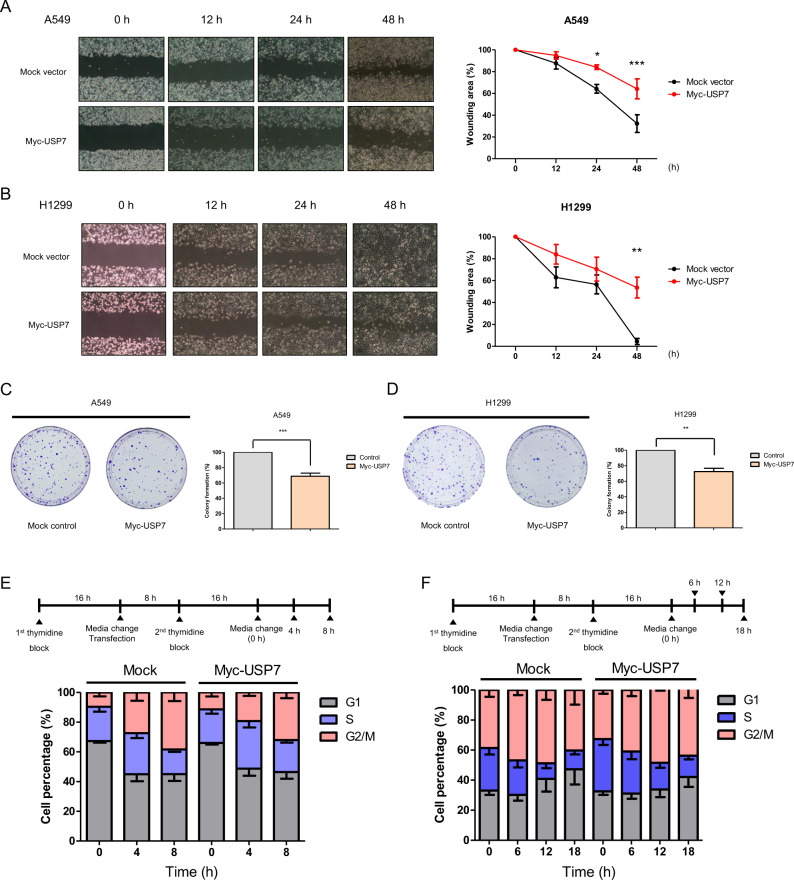
USP7 inhibits LUAD cell proliferation via modulation of the ERK1/2 signaling pathway. **A**, **B** Wound healing assays were performed in Myc-*USP7*-transfected A549 and H1299 cells and mock vector-transfected A549 and H1299 cells as controls. And cell images were captured at 0, 12, 24, and 48 h after scratch. Wound area (%) was determined by the rate of remaining wound area compared to control, *p < 0.05, ***p < 0.001, *n* = 4 (A549), *n* = 3 (H1299). **C**, **D** Colony-forming assays were performed in Myc-*USP7*-transfected A549 and H1299 cells and mock vector-transfected A549 and H1299 cells as controls. Cells were stained with crystal violet solution and cell images were captured. The number of colonies is analyzed by image J and calculated by a two-tailed Student’s *t*-test, *p < 0.05, **p < 0.01, *n* = 6 (A549), *n* = 4 (H1299). **E** Cell cycle a*n*alysis were performed in Myc-*USP7*-transfected H1299 cells and mock vector-transfected H1299 cells as controls with flow cytometry. Cells were synchronized by double thymidine block and fixed by 70% ethanol and stained by FxCycle™ PI/RNase staining solution to analyze the cell cycle, and the percentage of cells in G1, S, and G2/M phases was measured in a time-dependent manner (0, 4, and 8 h). Representative data was shown from at least three independent experiments. **F** A549 cells were also synchronized by double thymidine block and fixed by 70% ethanol and stained by FxCycle™ PI/RNase staining solution to analyze the cell cycle, and the percentage of cells in G1, S, and G2/M phases was measured in a time-dependent manner (0, 6, 12, and 18 h). Representative data was shown from three independent experiments.

Activation of the ERK1/2 signaling pathway is implicated in cancer cell proliferation, migration, invasion, metastasis, and tumor angiogenesis [1]. Activation of the ERK1/2 signaling pathway by oncogenic mutation has been reported in LUAD, and inhibitors targeting ERK1/2 signaling components are a promising cancer therapeutic strategy [52]. Until now, studies conducted on the cellular function of USP7 in LUAD have mostly focused on knockdown or inactivation of USP7, whereas the effects of USP7 overexpression in LUAD have not been investigated. In our study, we confirmed that USP7 overexpression inhibits the ERK1/2 signaling pathway in a p53-independent manner in LUAD cell lines. Therefore, regardless of the results of USP7 knockdown, overexpression of USP7 in LUAD is expected to affect cell proliferation by regulating the ERK1/2 signaling pathway via a novel mechanism that deubiquitinates Raf-1 in a p53-independent manner. To examine the effect of USP7 overexpression on lung cancer, cell proliferation and migration were confirmed by applying the colony-forming assay and wound healing assay. Wound healing coverage of Myc-*USP7*-transfected A549 and H1299 cells decreases when compared with a mock control. Moreover, the colony-forming efficiency of Myc-*USP7-*transfected A549 and H1299 cells was also decreased, as compared with the mock control (Fig. 5A–D). These results indicate that USP7 overexpression inhibits lung cancer cell proliferation and migration. However, USP7 overexpression does not induce early and late apoptosis in A549 and H1299 cells (Supplement Fig. S5). Therefore, the effect of USP7 on the cell cycle was investigated using FACS analysis. Synchronized H1299 and A549 cells by double thymidine block were harvested after release in a time-dependent manner, and we observed that compared with a mock control, USP7 overexpression decreases the number of cells in G2/M phase compared to a mock control (Fig. 5E, F). Collectively, these results indicate that overexpression of USP7 inhibits LUAD cell proliferation by suppressing the ERK1/2 signaling pathway in a p53-independent manner.

## Discussion

Of the nine DUB families, USP7 is a cysteine peptidase that belongs to the USP family. Being one of the well-studied DUBs, many substrate proteins of USP7 have been identified. It is reported that the TRAF and the Ubl2 domains of USP7 bind to the [P/A/E]-X-X-S and K-X-X-X-K protein sequences of substrate proteins, respectively [25, 53]. However, studies have not revealed the binding motif of the proteins to which USP7 binds, and studies to identify the novel binding proteins of USP7 using [P/A/E]-X-X-S and K-X-X-X-K binding motifs are insufficient. In the current study, we applied the motif analysis using bioinformatics tools, focusing on both the [P/A/E]-X-X-S and K-X-X-X-K motifs to identify new substrate proteins of USP7. Through DAVID analysis and protein-protein interaction analysis, we selected putative substrate proteins from about 18,000 proteins having both binding motifs. Several proteins with known binding to USP7, such as p53, TRAF6, death-domain associated protein (DAXX), and avian reticuloendotheliosis viral oncogene homolog A (RELA), as well as proteins where binding has not yet been identified, were detected [24, 54–56]. This indicates that the analysis methods applied in this study are effective assays for discovering substrate proteins of USP7. Although we confirmed the binding between putative substrates and USP7, some proteins did not actually bind to USP7 even though they had both the [P/A/E]-X-X-S and K-X-X-X-K motifs. There may be several causes that are yet to be elucidated, but it is expected that the diversity of the protein structure due to the inherent protein sequence may have influenced the binding of USP7 [25]. Therefore, further studies on the specific molecular mechanism of USP7 through which it associates with the binding motifs of substrate proteins will help identify novel substrates of USP7.

Until now, USP7 has been shown to function in numerous cellular functions, and a recent study revealed that the DUB activity of USP7 functions as a regulator in various signaling pathways such as Hippo, Wnt, PI3K/AKT, and IFN via deubiquitination of substrate proteins [15–17, 57, 58]. The relationship between the MAPK signaling pathway and USP7 is yet to be elucidated, but in this study, through DAVID analysis, we proved putative binding proteins are related to the MAPK signaling pathway in addition to Rap1 and cAMP signaling pathways. These results will aid in discovering novel substrates and functions of USP7 in the diverse signaling pathways.

In addition, the polyubiquitin chains of Raf-1 have not been fully understood, but our results show that K6-, K11-, K29-, K33-, and K48-linked polyubiquitin chains of Raf-1 are related to proteasomal degradation of Raf-1. It is already known that K11-, K29-, and K48-linked polyubiquitination is mainly associated with proteasomal degradation of target proteins. But in this study, we discovered that K6- and K33-linked polyubiquitinations are related to proteasomal degradation. Therefore, further studies on the mechanism of proteasomal degradation by K6- and K33-linked polyubiquitinations will help to elucidate an important role in controlling the stability of several proteins.

**Fig. 6:**
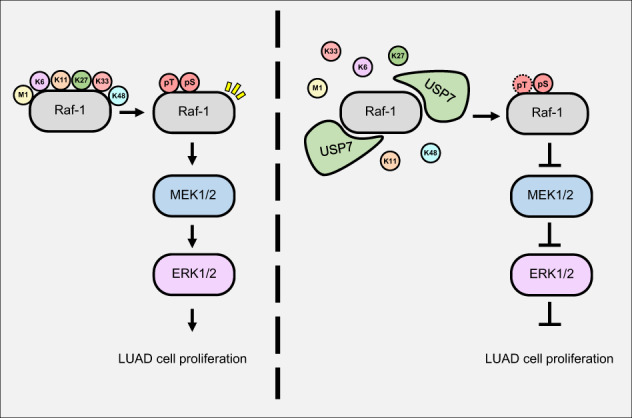
A model for USP7 mediating the ERK1/2 signaling pathway through deubiquitination of Raf-1. USP7 deubiquitinates M1-, K6-, K11-, K27-, K33-, K48-linked polyubiquitin chains of Raf-1. And USP7 decreases the phosphorylation level of Raf-1 disrupting MEK1/2-ERK1/2 signal transduction through Raf activation. Inactivation of ERK1/2 signaling pathway inhibits the proliferation of lung adenosarcoma cells though delaying the G2/M phase.

We also discovered that M1-, K6-, K11-, K27-, K33-, and K48-linked polyubiquitin chains of Raf-1 are deubiquitinated by USP7. However, it is not known which M1- or K-linked polyubiquitin chain of Raf-1 is related to the activity of Raf-1. On the other hand, kinase activity of B-Raf (another Raf family isoform) is increased by K27-linked polyubiquitination via the E3 ligase activity of itchy E3 ubiquitin protein ligase (ITCH) [49]. Since the Raf family has common structural features, such as having three CR domains, the K27-linked polyubiquitination of Raf-1 is also expected to be related to kinase activity [59]. However, to regulate the activity of Raf-1 by USP7, additional studies on Raf-1 polyubiquitin chains related to kinase activity are required. While USP7 decreases K6- and K33-linked polyubiquitination of Raf-1 related to proteasomal degradation, USP7 does not regulate the stability of Raf-1 unlike CTLH and HUWE1, which were identified as E3 ligases of Raf-1. Even though, the protein stability of Raf-1 is not modulated by USP7, the threonine phosphorylation level of Raf-1 and activation of Raf-1 are inhibited by USP7. Threonine phosphorylation of Raf-1 is known to occur at Thr268, Thr269, and Thr491. Phosphorylation of Thr268 and Thr269 are autophosphorylation, and are not related to the activation of Raf [14, 60]. Phosphorylation of Thr491 occurs in the activation loop and a Thr491 mutant inhibits the full activation of Raf-1. However, the phosphorylation of Thr491 is insufficient to activate Raf-1. Kinases and phosphatases that regulate serine phosphorylation of Raf-1 have been elucidated such as PP2A and protein kinase A (PKA), but diverse research has not been performed on the proteins that regulate threonine phosphorylation of Raf-1. Therefore, the DUB activity of USP7 may play an important role in elucidating the regulatory mechanism of threonine phosphorylation of Raf-1. Therefore, further research on the ubiquitination sites of Raf-1 regulated by E3 ligases or USP7 is expected to modulate the ERK1/2 signaling pathway by selectively regulating the stability or activity of Raf-1, which could be a new therapeutic strategy in the ERK1/2 cascade activated cancers (Fig. 6).

Raf-MEK-ERK cascade is a key signaling pathway that modulates diverse cellular processes such as cell proliferation, differentiation, and apoptosis [1, 4]. Also, dysfunction of the ERK1/2 signaling pathway and mutants of ERK1/2 signaling components such as B-Raf (V600E) and K-Ras (G12D) are the causes of several cancers and diseases, including neurodegeneration diseases [6, 61, 62]. Therefore, several inhibitory proteins such as Raf kinase inhibitor protein (RKIP), and specific inhibitors such as Vemurafenib (B-Raf V600E inhibitor), Trametinib (MEK1/2 inhibitor), and Gefitinib (EGFR inhibitor) that regulate the Raf-MEK-ERK cascade, have been studied. However, inhibition of the ERK1/2 signaling pathway using each inhibitor induces drug resistance by the negative feedback loop of the ERK1/2 signaling pathway. Therefore, the use of Raf and MEK inhibitors as a combination drug is commonly used in various cancer types, including malignant melanoma [63]. Contrarily, our study showed that overexpression of USP7 inhibits the phosphorylation of Raf-1, MEK, and ERK1/2 through deubiquitination of Raf-1. On the other hand, the USP7-MDM2/MDMX-p53 axis plays an important role in cancer proliferation, metastasis, and growth in various cancer types. USP7 is a deubiquitinating enzyme that regulates both MDM2/MDMX and p53 proteins and acts as an oncoprotein or a tumor suppressor depending on the regulation direction [18]. Most studies have focused on cancer therapy through the regulation of two proteins, MDM2/MDMX and p53. Similarly, in our study, we confirmed that knockdown of USP7 increases the p53 expression. Conversely, USP7 overexpression is expected to decrease p53 expression, but does not affect p53 protein expression. Also, USP7 overexpression suppresses LUAD cell growth through inhibition of the ERK1/2 signaling pathway independently of p53. As a result, we determined that the balance of expression of USP7 plays an important role in the tumorigenesis of LUAD. In addition, although it is necessary to further study whether USP7 regulates the ERK1/2 signaling pathway via the same mechanism in other cancer cells, inhibition of the ERK1/2 signaling pathway through USP7 could be a good target for new cancer therapeutics along with p53/MDM2 targeting.

In the current study, we identified that USP7 binds to the PVDS motif in the CR3 domain of Raf-1 through the binding motif and PPI analyses. We also found that USP7 deubiquitinates M1-, K6-, K11-, K27-, K33-, and K48-linked polyubiquitin chains of Raf-1. Moreover, the DUB activity of USP7 reduces the threonine phosphorylation of Raf-1 and inhibits the ERK1/2 signaling pathway through the suppression of p-Raf. Overexpression of USP7 was found to inhibit the ERK1/2 signaling pathway and induce cell cycle delay. Taken together, we deduce that USP7 suppresses LUAD cell proliferation. Therefore, we believe that USP7 has the potential to be applied as a novel regulator in the ERK1/2 signaling pathway.

### Reporting summary

Further information on research design is available in the Nature Research Reporting Summary linked to this article.

## Supplementary information

Reporting Summary

Supplemental information

Datasheet 1

Original Data File

## Data Availability

Data will be made available on reasonable request.

## References

[1] Guo YJ, Pan WW, Liu SB, Shen ZF, Xu Y, Hu LL (2020). ERK/MAPK signalling pathway and tumorigenesis. Exp Ther Med.

[2] McCain J (2013). The MAPK (ERK) pathway: investigational combinations for the treatment of BRAF-mutated metastatic melanoma. P T..

[3] Lu Y, Liu B, Liu Y, Yu X, Cheng G (2020). Dual effects of active ERK in cancer: a potential target for enhancing radiosensitivity. Oncol Lett.

[4] Sun Y, Liu WZ, Liu T, Feng X, Yang N, Zhou HF (2015). Signaling pathway of MAPK/ERK in cell proliferation, differentiation, migration, senescence and apoptosis. J Recept Signal Transduct Res.

[5] Dhillon AS, Hagan S, Rath O, Kolch W (2007). MAP kinase signalling pathways in cancer. Oncogene.

[6] Degirmenci U, Wang M, Hu J (2020). Targeting aberrant RAS/RAF/MEK/ERK signaling for cancer therapy. Cells.

[7] Roskoski R (2010). RAF protein-serine/threonine kinases: structure and regulation. Biochem Biophys Res Commun.

[8] Margolis B, Skolnik EY (1994). Activation of ras by receptor tyrosine kinases. J Am Soc Nephrol.

[9] Watanabe T, Shinohara N, Moriya K, Sazawa A, Kobayashi Y, Ogiso Y (2000). Significance of the Grb2 and son of sevenless (Sos) proteins in human bladder cancer cell lines. IUBMB Life.

[10] Santarpia L, Lippman SM, El-Naggar AK (2012). Targeting the MAPK-RAS-RAF signaling pathway in cancer therapy. Expert Opin Ther Targets.

[11] Maurer G, Tarkowski B, Baccarini M (2011). Raf kinases in cancer-roles and therapeutic opportunities. Oncogene.

[12] McTavish C, Bérubé-Janzen W, Wang X, Maitland MER, Salemi LM, Hess DA (2019). Regulation of c-Raf stability through the CTLH complex. Int J Mol Sci.

[13] Dhillon AS, Meikle S, Yazici Z, Eulitz M, Kolch W (2002). Regulation of Raf-1 activation and signalling by dephosphorylation. Embo j.

[14] Zang M, Gong J, Luo L, Zhou J, Xiang X, Huang W (2008). Characterization of Ser338 phosphorylation for Raf-1 activation. J Biol Chem.

[15] Sun X, Ding Y, Zhan M, Li Y, Gao D, Wang G (2019). USP7 regulates Hippo pathway through deubiquitinating the transcriptional coactivator Yorkie. Nat Commun.

[16] Ji L, Lu B, Zamponi R, Charlat O, Aversa R, Yang Z (2019). USP7 inhibits Wnt/β-catenin signaling through promoting stabilization of axin. Nat Commun.

[17] Cai J, Chen HY, Peng SJ, Meng JL, Wang Y, Zhou Y (2018). USP7-TRIM27 axis negatively modulates antiviral type I IFN signaling. Faseb j.

[18] Qi SM, Cheng G, Cheng XD, Xu Z, Xu B, Zhang WD (2020). Targeting USP7-mediated deubiquitination of MDM2/MDMX-p53 pathway for cancer therapy: are we there yet?. Front Cell Dev Biol.

[19] Choi HS, Pei CZ, Park JH, Kim SY, Song SY, Shin GJ (2020). Protein stability of pyruvate kinase isozyme M2 is mediated by HAUSP. Cancers (Basel).

[20] Jagannathan M, Nguyen T, Gallo D, Luthra N, Brown GW, Saridakis V (2014). A role for USP7 in DNA replication. Mol Cell Biol.

[21] Georges A, Coyaud E, Marcon E, Greenblatt J, Raught B, Frappier L (2019). USP7 regulates cytokinesis through FBXO38 and KIF20B. Sci Rep..

[22] Zemp I, Lingner J (2014). The shelterin component TPP1 is a binding partner and substrate for the deubiquitinating enzyme USP7. J Biol Chem.

[23] Sarkari F, Wheaton K, La Delfa A, Mohamed M, Shaikh F, Khatun R (2013). Ubiquitin-specific protease 7 is a regulator of ubiquitin-conjugating enzyme UbE2E1. J Biol Chem.

[24] Wang Z, Kang W, You Y, Pang J, Ren H, Suo Z (2019). USP7: novel drug target in cancer therapy. Front Pharm.

[25] Cheng J, Li Z, Gong R, Fang J, Yang Y, Sun C (2015). Molecular mechanism for the substrate recognition of USP7. Protein Cell.

[26] Gridelli C, Rossi A, Carbone DP, Guarize J, Karachaliou N, Mok T (2015). Non-small-cell lung cancer. Nat Rev Dis. Prim.

[27] Thai AA, Solomon BJ, Sequist LV, Gainor JF, Heist RS (2021). Lung cancer. Lancet.

[28] Anusewicz D, Orzechowska M, Bednarek AK (2020). Lung squamous cell carcinoma and lung adenocarcinoma differential gene expression regulation through pathways of Notch, Hedgehog, Wnt, and ErbB signalling. Sci Rep..

[29] Masuya D, Huang C, Liu D, Nakashima T, Yokomise H, Ueno M (2006). The HAUSP gene plays an important role in non-small cell lung carcinogenesis through p53-dependent pathways. J Pathol.

[30] Dai X, Lu L, Deng S, Meng J, Wan C, Huang J (2020). USP7 targeting modulates anti-tumor immune response by reprogramming tumor-associated macrophages in lung cancer. Theranostics.

[31] Zhao GY, Lin ZW, Lu CL, Gu J, Yuan YF, Xu FK (2015). USP7 overexpression predicts a poor prognosis in lung squamous cell carcinoma and large cell carcinoma. Tumour Biol.

[32] Zhang C, Lu J, Zhang QW, Zhao W, Guo JH, Liu SL (2016). USP7 promotes cell proliferation through the stabilization of Ki-67 protein in non-small cell lung cancer cells. Int J Biochem Cell Biol.

[33] Huang YT, Cheng AC, Tang HC, Huang GC, Cai L, Lin TH (2021). USP7 facilitates SMAD3 autoregulation to repress cancer progression in p53-deficient lung cancer. Cell Death Dis.

[34] Park JH, Kim SY, Cho HJ, Lee SY, Baek KH (2020). YOD1 deubiquitinates NEDD4 involved in the hippo signaling pathway. Cell Physiol Biochem.

[35] Huang da W, Sherman BT, Lempicki RA (2009). Systematic and integrative analysis of large gene lists using DAVID bioinformatics resources. Nat Protoc.

[36] Bufalieri F, Lospinoso Severini L, Caimano M, Infante P, Di Marcotullio L (2020). DUBs activating the hedgehog signaling pathway: a promising therapeutic target in cancer. Cancers (Basel).

[37] Doncheva NT, Morris JH, Gorodkin J, Jensen LJ (2019). Cytoscape stringApp: network analysis and visualization of proteomics data. J Proteome Res.

[38] Oughtred R, Stark C, Breitkreutz BJ, Rust J, Boucher L, Chang C (2019). The BioGRID interaction database: 2019 update. Nucleic Acids Res.

[39] Baek KH, Fabian JR, Sprenger F, Morrison DK, Ambrosio L (1996). The activity of D-raf in torso signal transduction is altered by serine substitution, N-terminal deletion, and membrane targeting. Dev Biol.

[40] Park JJ, Lim KH, Baek KH (2015). Annexin-1 regulated by HAUSP is essential for UV-induced damage response. Cell Death Dis.

[41] Dougherty MK, Müller J, Ritt DA, Zhou M, Zhou XZ, Copeland TD (2005). Regulation of Raf-1 by direct feedback phosphorylation. Mol Cell.

[42] Lim KH, Joo JY, Baek KH (2020). The potential roles of deubiquitinating enzymes in brain diseases. Ageing Res Rev.

[43] Jang ER, Shi P, Bryant J, Chen J, Dukhande V, Gentry MS (2014). HUWE1 is a molecular link controlling RAF-1 activity supported by the Shoc2 scaffold. Mol Cell Biol.

[44] Fan Q, Wang Q, Cai R, Yuan H, Xu M (2020). The ubiquitin system: orchestrating cellular signals in non-small-cell lung cancer. Cell Mol Biol Lett.

[45] Lim K-H, Park J-J, Gu B-H, Kim J-O, Park SG, Baek K-H (2015). HAUSP-nucleolin interaction is regulated by p53-Mdm2 complex in response to DNA damage response. Sci Rep..

[46] Galarreta A, Valledor P, Ubieto-Capella P, Lafarga V, Zarzuela E, Muñoz J (2021). USP7 limits CDK1 activity throughout the cell cycle. Embo j.

[47] Kim SH, Baek KH (2021). Regulation of cancer metabolism by deubiquitinating enzymes: the warburg effect. Int J Mol Sci.

[48] Prescott JA, Mitchell JP, Cook SJ (2021). Inhibitory feedback control of NF-κB signalling in health and disease. Biochem J.

[49] Yin Q, Han T, Fang B, Zhang G, Zhang C, Roberts ER (2019). K27-linked ubiquitination of BRAF by ITCH engages cytokine response to maintain MEK-ERK signaling. Nat Commun.

[50] Cekanova M, Majidy M, Masi T, Al-Wadei HA, Schuller HM (2007). Overexpressed Raf-1 and phosphorylated cyclic adenosine 3'-5'-monophosphatate response element-binding protein are early markers for lung adenocarcinoma. Cancer.

[51] Moon A, Park JY, Sung JY, Park YK, Kim YW (2012). Reduced expression of Raf-1 kinase inhibitory protein in renal cell carcinoma: a significant prognostic marker. Pathology.

[52] Cristea S, Sage J (2016). Is the canonical RAF/MEK/ERK signaling pathway a therapeutic target in SCLC?. J Thorac Oncol.

[53] Georges A, Marcon E, Greenblatt J, Frappier L (2018). Identification and characterization of USP7 targets in cancer cells. Sci Rep..

[54] Mitxitorena I, Somma D, Mitchell JP, Lepistö M, Tyrchan C, Smith EL (2020). The deubiquitinase USP7 uses a distinct ubiquitin-like domain to deubiquitinate NF-ĸB subunits. J Biol Chem.

[55] Xiang Q, Ju H, Nicholas J (2020). USP7-dependent regulation of TRAF activation and signaling by a viral interferon regulatory factor homologue. J Virol.

[56] Sheng Y, Saridakis V, Sarkari F, Duan S, Wu T, Arrowsmith CH (2006). Molecular recognition of p53 and MDM2 by USP7/HAUSP. Nat Struct Mol Biol.

[57] Ye M, He J, Zhang J, Liu B, Liu X, Xie L (2021). USP7 promotes hepatoblastoma progression through activation of PI3K/AKT signaling pathway. Cancer Biomark.

[58] Al-Eidan A, Wang Y, Skipp P, Ewing RM (2022). The USP7 protein interaction network and its roles in tumorigenesis. Genes Dis.

[59] Migliaccio N, Sanges C, Ruggiero I, Martucci NM, Rippa E, Arcari P (2013). Raf kinases in signal transduction and interaction with translation machinery. Biomol Concepts.

[60] Chong H, Lee J, Guan KL (2001). Positive and negative regulation of Raf kinase activity and function by phosphorylation. Embo j.

[61] Albert-Gascó H, Ros-Bernal F, Castillo-Gómez E, Olucha-Bordonau FE (2020). MAP/ERK signaling in developing cognitive and emotional function and its effect on pathological and neurodegenerative processes. Int J Mol Sci.

[62] Kim EK, Choi EJ (2010). Pathological roles of MAPK signaling pathways in human diseases. Biochim Biophys Acta.

[63] Lee S, Rauch J, Kolch W (2020). Targeting MAPK signaling in cancer: mechanisms of drug resistance and sensitivity. Int J Mol Sci.

