# Supplementary material for: USP7 regulates the ERK1/2 signaling pathway through deubiquitinating Raf-1 in lung adenocarcinoma
Source: Cell Death Dis. 2022 Aug 10;13(8):698. doi: 10.1038/s41419-022-05136-6 (PMC9365811; doi:10.1038/s41419-022-05136-6)
Supplement: Supplementary file 2 — Supplemental information [file 41419_2022_5136_MOESM2_ESM.docx]

Supplementary Information for

**USP7 regulates the ERK1/2 signaling pathway through deubiquitinating Raf-1 in lung adenocarcinoma**

Hong-Beom Park^1^, Sohyun Hwang^1,2^, and Kwang-Hyun Baek^1,*^,

*This file contains the following contents:

Supplementary Figures and Figure Legends

Supplementary Tables


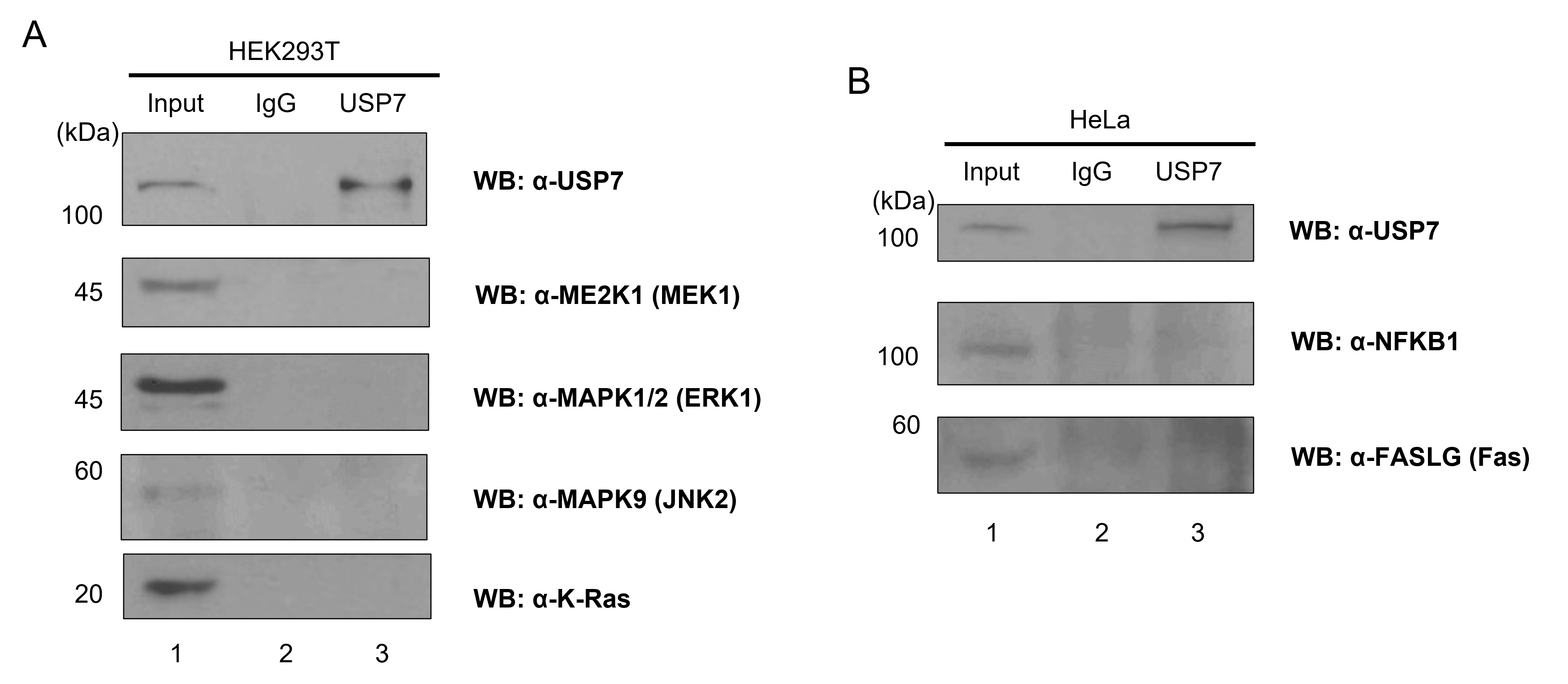


**Supplement Figure S1. Immunoprecipitation between putative substrate proteins and USP7.** (A) HeLa cell lysate was precipitated by an anti-USP7 and control IgG antibodies. Anti-NFKB1 and anti-FASLG antibodies were used for immunoblotting. (B) And HEK293T cell lysate also was precipitated by anti-USP7 and control IgG antibodies. Anti-ME2K1, anti-MAPK1/2, anti-MAPK9, and anti-K-Ras antibodies were used for immunoblotting.


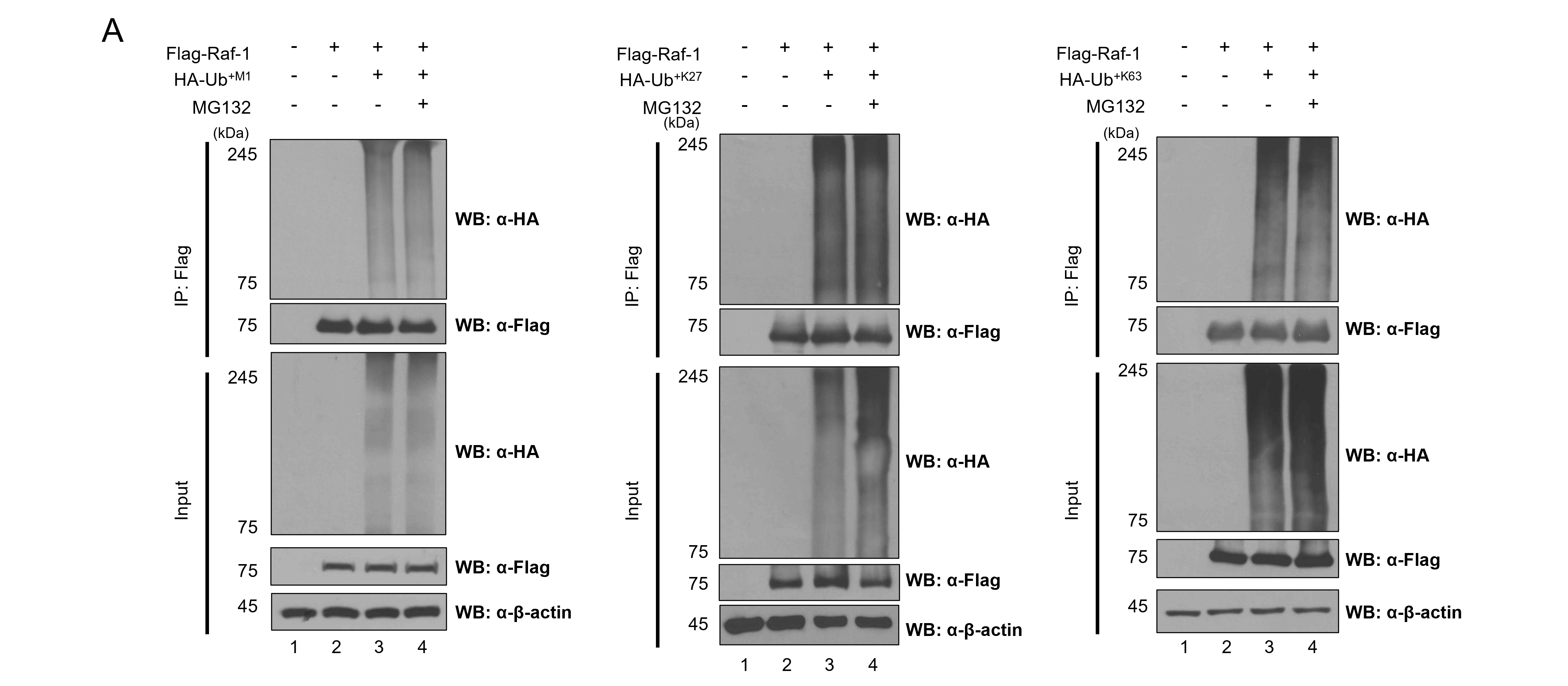

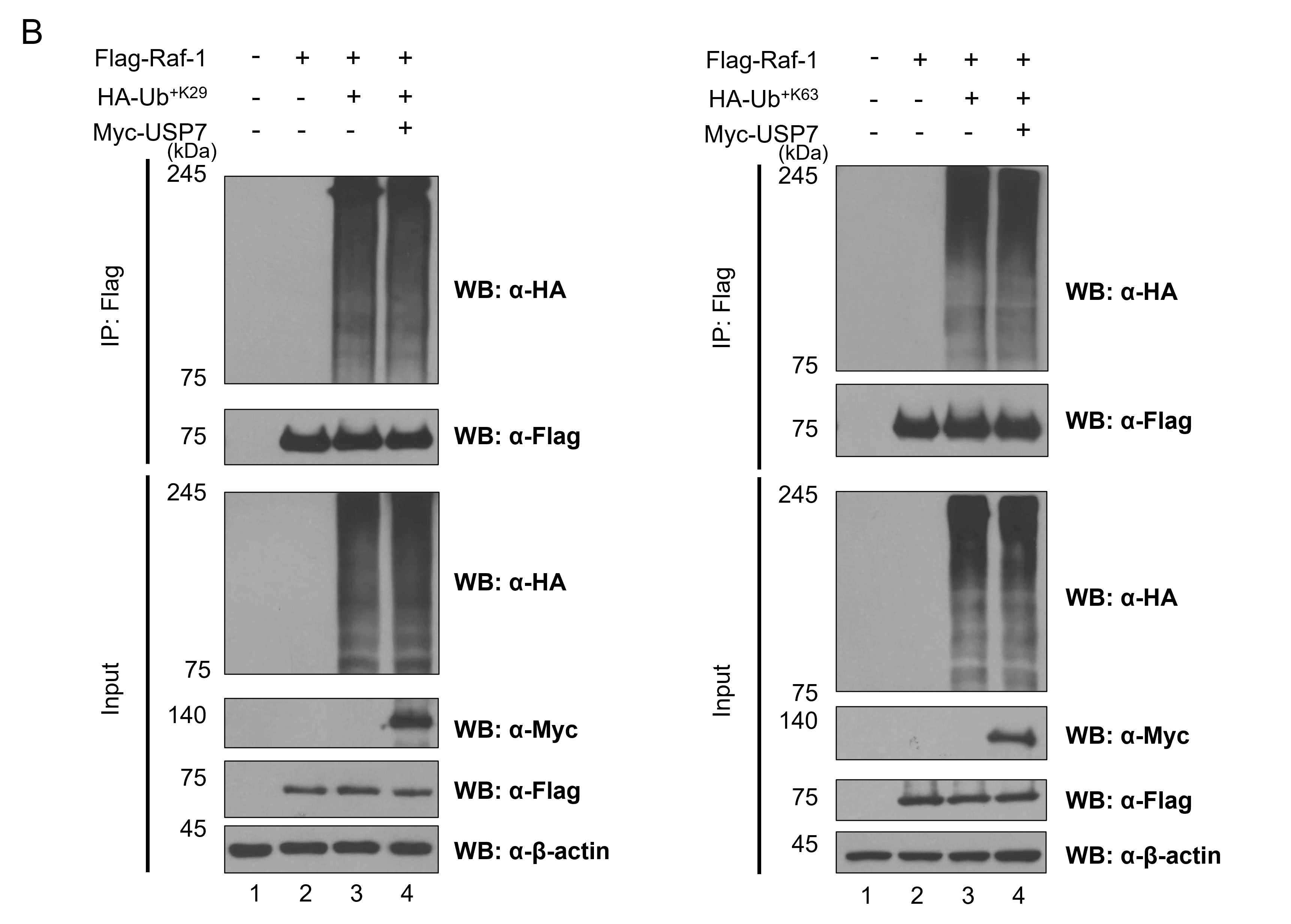


**Supplement Figure S2. M1-, K27, and K63-linked polyubiquitin chains of Raf-1 is not related with UPS and USP7 does not regulates K29- and K63-linked polyubiquitin chains of Raf-1.** (A) HEK293T cells were transfected with Flag*-Raf-1* and each HA*-Ub* mutant (M1, K27, and K63) and treated with a proteasome inhibitor MG132. And then ubiquitination assay of Raf-1 was performed for checking association between specific lysine site of polyubiquitination and UPS. (B) HEK293T cells were transfected with each HA*-Ub* mutant (K29 and K63) and Myc*-USP7*. A change in the polyubiquitin chains (K29 and K63) of Raf-1 by USP7 was detected through deubiquitination assay.


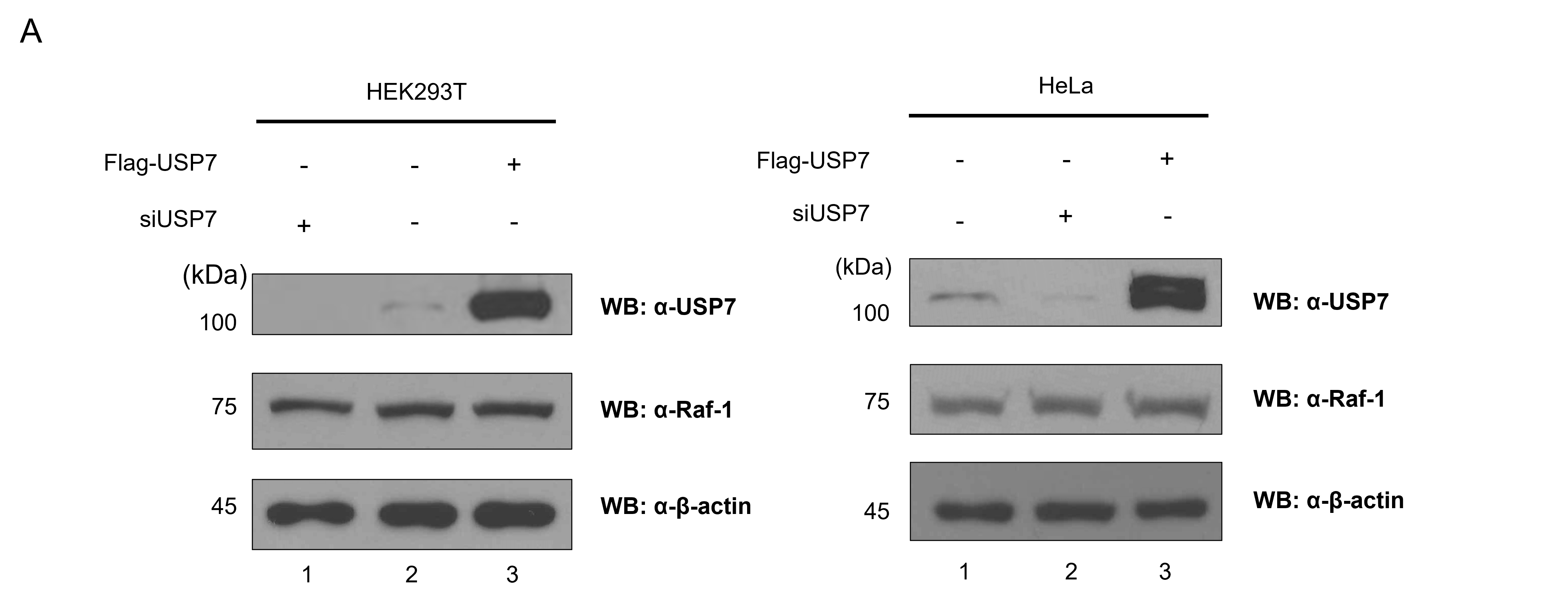


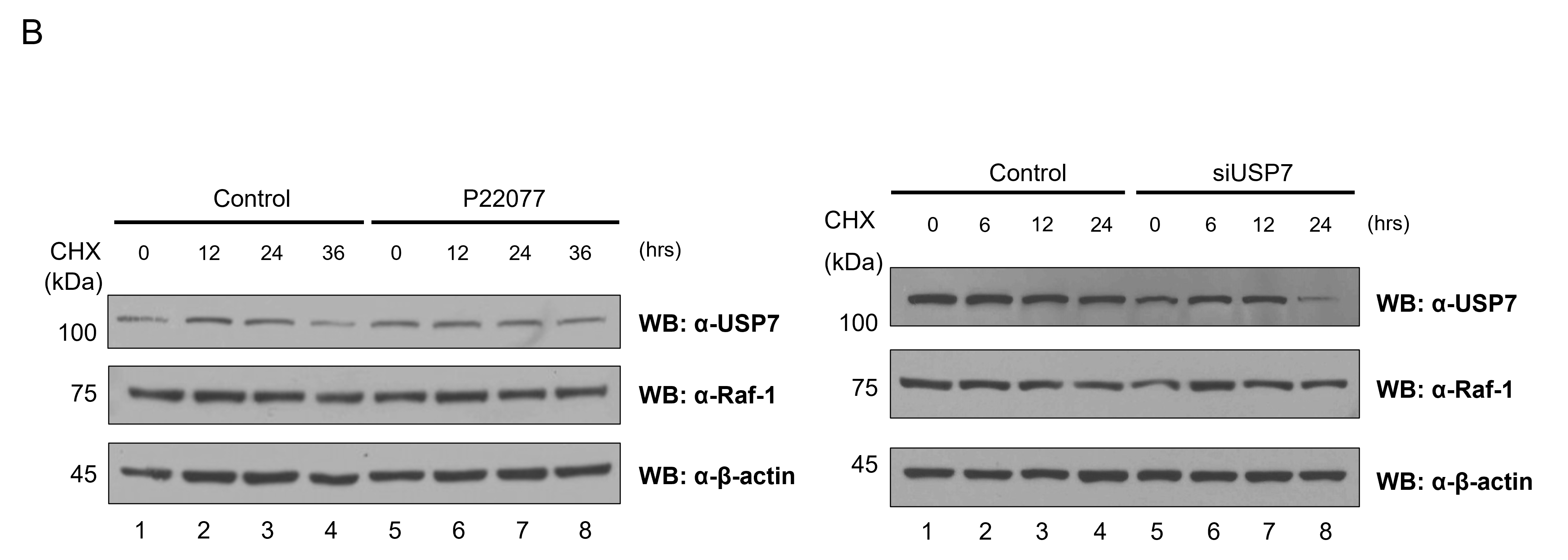


**Supplement Figure S3. The protein stability of Raf-1 is not regulated by the DUB activity of USP7.** (A) In HEK293T or HeLa cells, USP7 is overexpressed or downregulated using Flag*-USP7* or *siUSP7* constructs for checking the effects of USP7 in the protein stability of Raf-1. The experiment was performed at least three times and representative data were shown. (B) For protein stability assay, HEK293T cells were treated with CHX and P22077 or transfected with *siUSP7*. Cells were harvested in time-dependent manner and the half-life of Raf-1 is determined by WB.


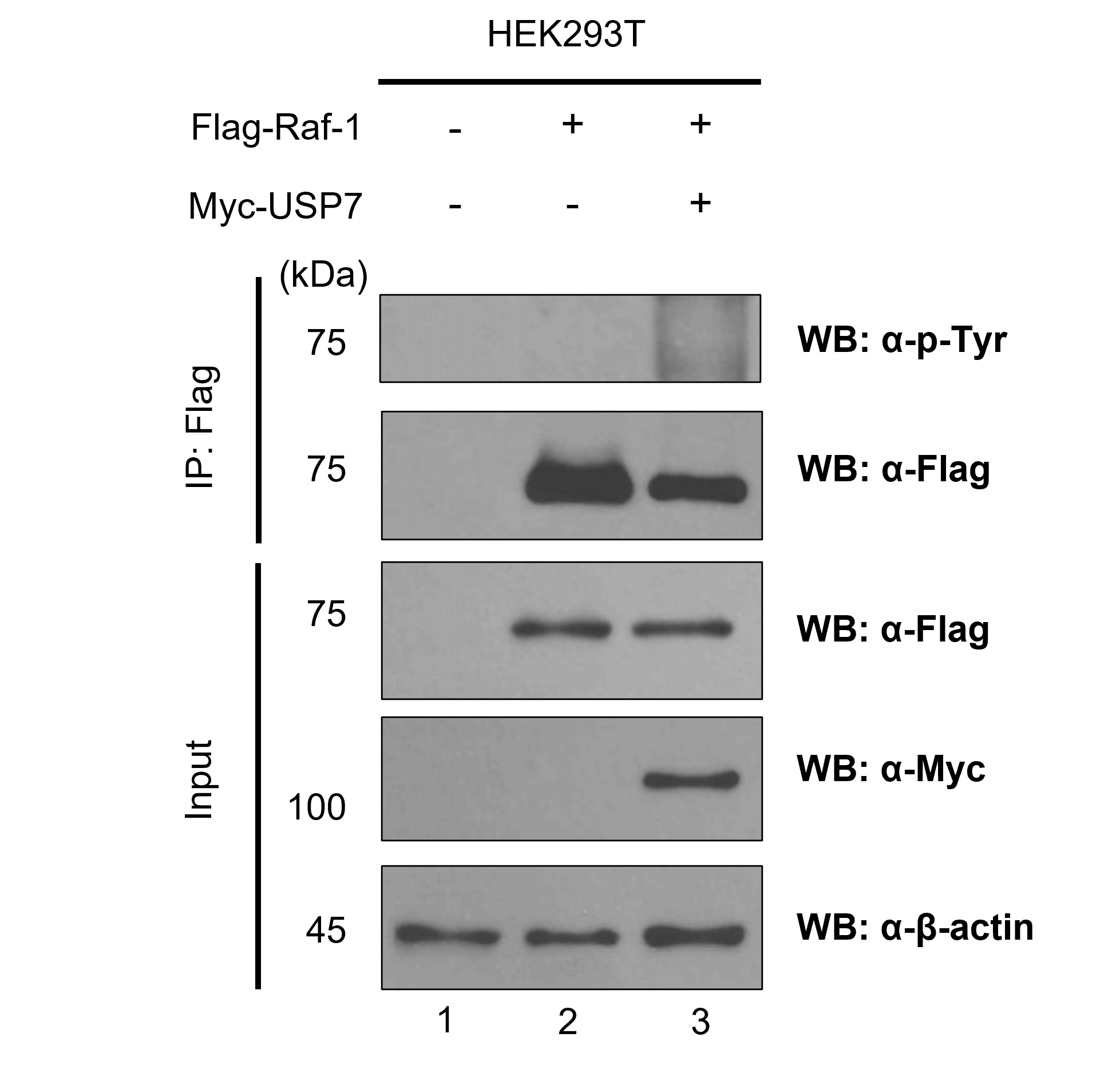


**Supplement Figure S4. Tyrosine phosphorylation level of Raf-1.** HEK293T cells were transfected with Flag*-Raf-1* and Myc*-USP7*, and cell lysates were precipitated by an anti-Flag antibody. And anti-p-Tyr antibody was used for immunoblotting. The expression of p-Tyr was not detected.


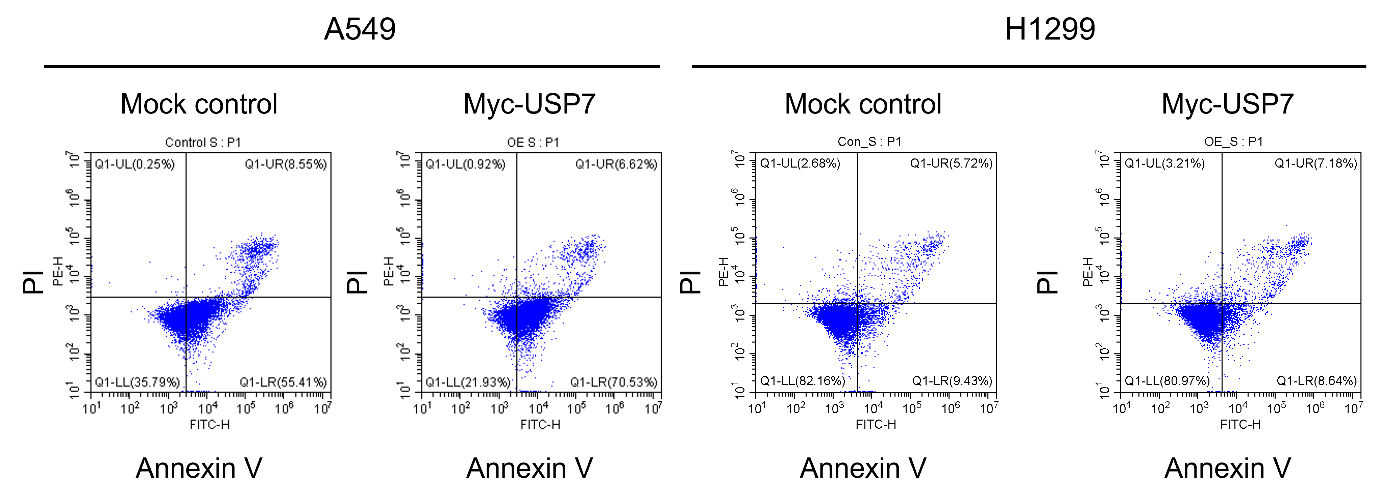


**Supplement Figure S5. USP7 overexpression dose not induces lung cancer cell apoptosis.** A549 or H1299 cells were transfected with mock vector or Myc*-USP7*. And annexin V and PI of 1x10^5^ cells are stained and early and late apoptosis of mock vector or Myc*-USP7*-transfected cells measured by FACS analysis.

**Supplementary Table.**

**Supplement Table S1. Oligos used in this study**

| Primer | Sequence (5’ to 3’) |
| --- | --- |
| Myc-USP7 (UBL) | FP: GGATCCCATCTCTATATG |
|  | RP: CTCGAGTCAGTTATGGAT |
| Myc-USP7 (TRAF) | FP: GGATCCATGAACCACCAG |
|  | RP: CTCGAGTCAATCCCACGC |
| Flag-Raf-1 (S274R) | FP: TGT GGA CAG AAG GAT GAT |
|  | RP: ATC ATC CTT CTG TCC ACA |
| Flag-Raf-1 (S283R) | FP: AAT TCG AAG ACA CAG CGA |
|  | RP: TCG CTG TGT CTT CGA ATT |
| Flag-Raf-1 (S289R) | FP: ATC AGC CCG ACC TTC AGC |
|  | RP: GCT GAA GGT CGG GCT GAT |
| Flag-Raf-1 (S291R) | FP: CTC ACC TCG AGC CCT GTC |
|  | RP: GAC AGG GCT CGA GGT GAG |
| Flag-Raf-1 (S295R) | FP: CCT GTC CAG AAG CCC CAA |
|  | RP: TTG GGG CTT CTG GAC AGG |
| Flag-Raf-1 (S322R) | FP: ACC AGT ACG TGG GAC CCA |
|  | RP: TGG GTC CCA CGT ACT GGT |
| Flag-Raf-1 (S428R) | FP: GGG CAG CAG ACT CTA CAA |
|  | RP: TTG TAG AGT CTG CTG CCC |
| Flag-Raf-1 (S508R) | FP: TAC TGG CCG TGT CCT CTG |
|  | RP: CAG AGG ACA CGG CCA GTA |
| Flag-Raf-1 (S571R) | FP: AGA TCT TAG AAA GCT ATA |
|  | RP: TAT AGC TTT CTA AGA TCT |
| Flag-Raf-1 (∆1) | FP: GAA TTC ATG GAG CAC ATA CA |
|  | RP: TCT AGA CCT CTG GGA GAG G |
| Flag-Raf-1 (∆2) | RP: TCT AGA AAT TTT GTT TTT CTC CT |
| Flag-Raf-1 (∆3) | FP: GAA TTC CAG AGG TCG ACA T |
|  | RP: TCT AGA CCT CTG GGA GAG G |
| Flag-Raf-1 (∆4) | FP: GAA TTC AGG CCT CGT GGA |
