# Supplementary material for: USP7 regulates the ERK1/2 signaling pathway through deubiquitinating Raf-1 in lung adenocarcinoma
Source: Cell Death Dis. 2022 Aug 10;13(8):698. doi: 10.1038/s41419-022-05136-6 (PMC9365811; doi:10.1038/s41419-022-05136-6)
Supplement: Supplementary file 4 — Original Data File [file 41419_2022_5136_MOESM4_ESM.pptx]

## Slide 1
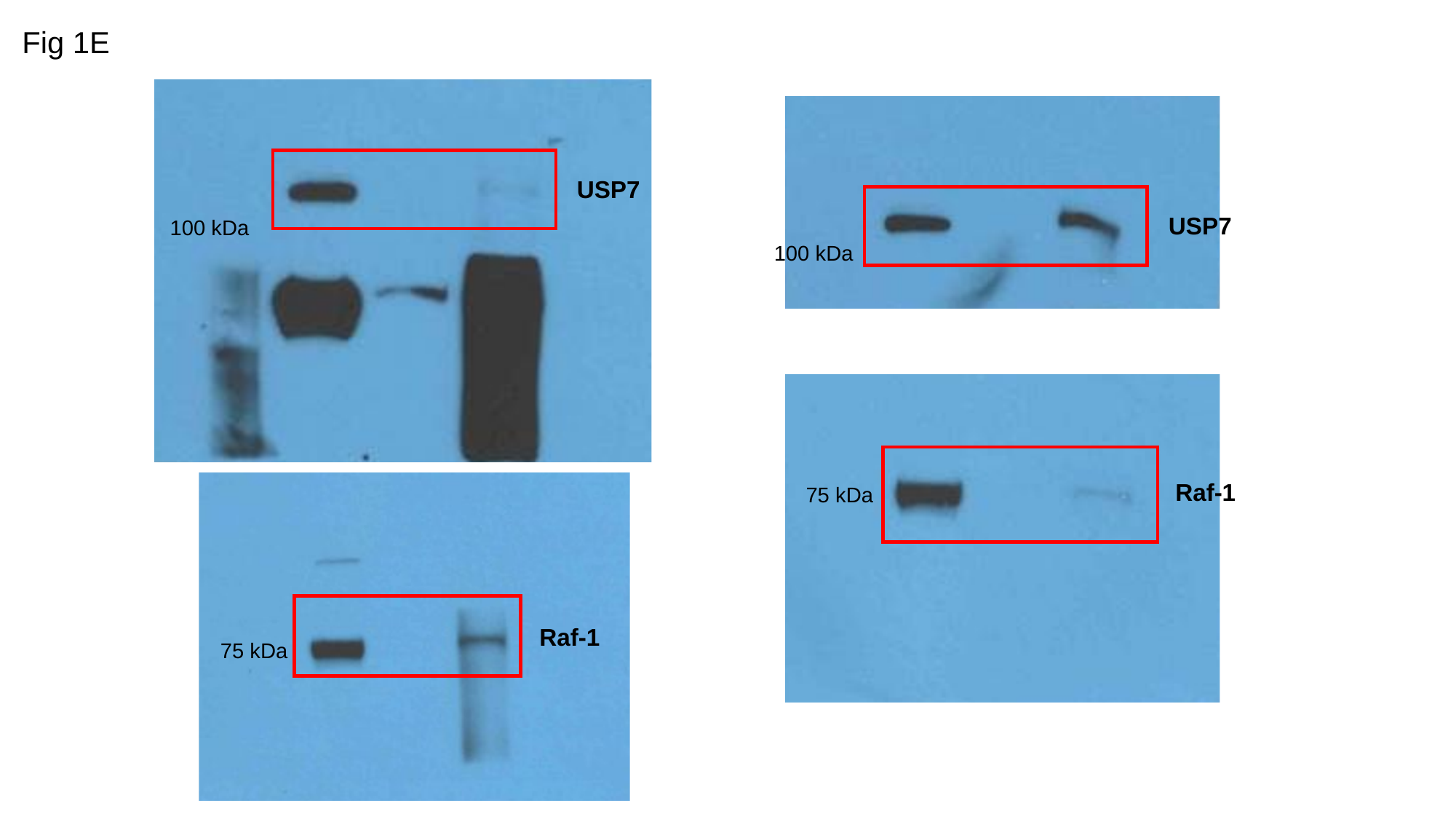

Fig 1E
USP7
USP7
100 kDa
100 kDa
Raf-1
75 kDa
Raf-1
75 kDa

## Slide 2
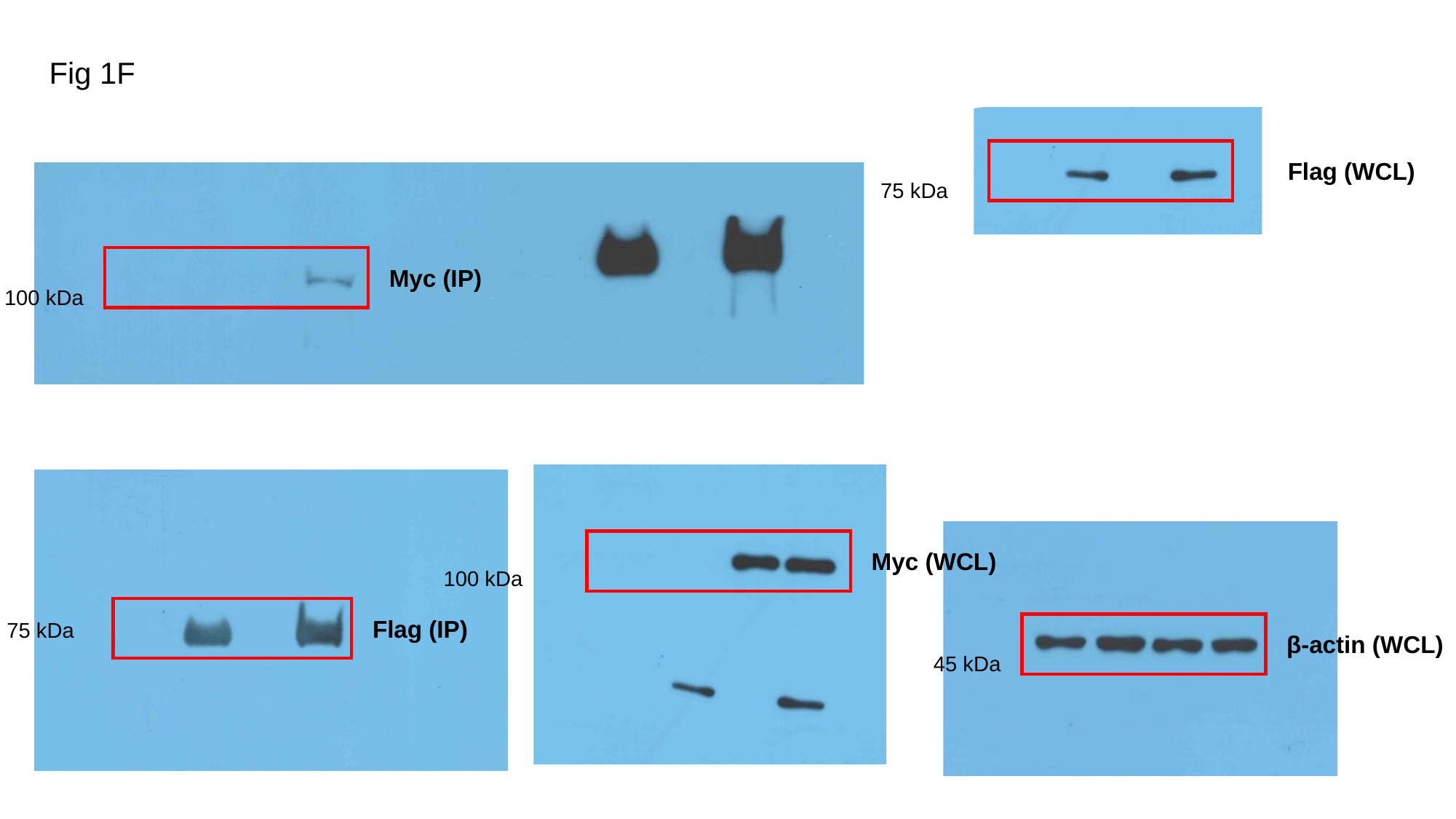

Fig 1F
Flag (WCL)
75 kDa
Myc (IP)
100 kDa
Myc (WCL)
100 kDa
Flag (IP)
75 kDa
β-actin (WCL)
45 kDa

## Slide 3
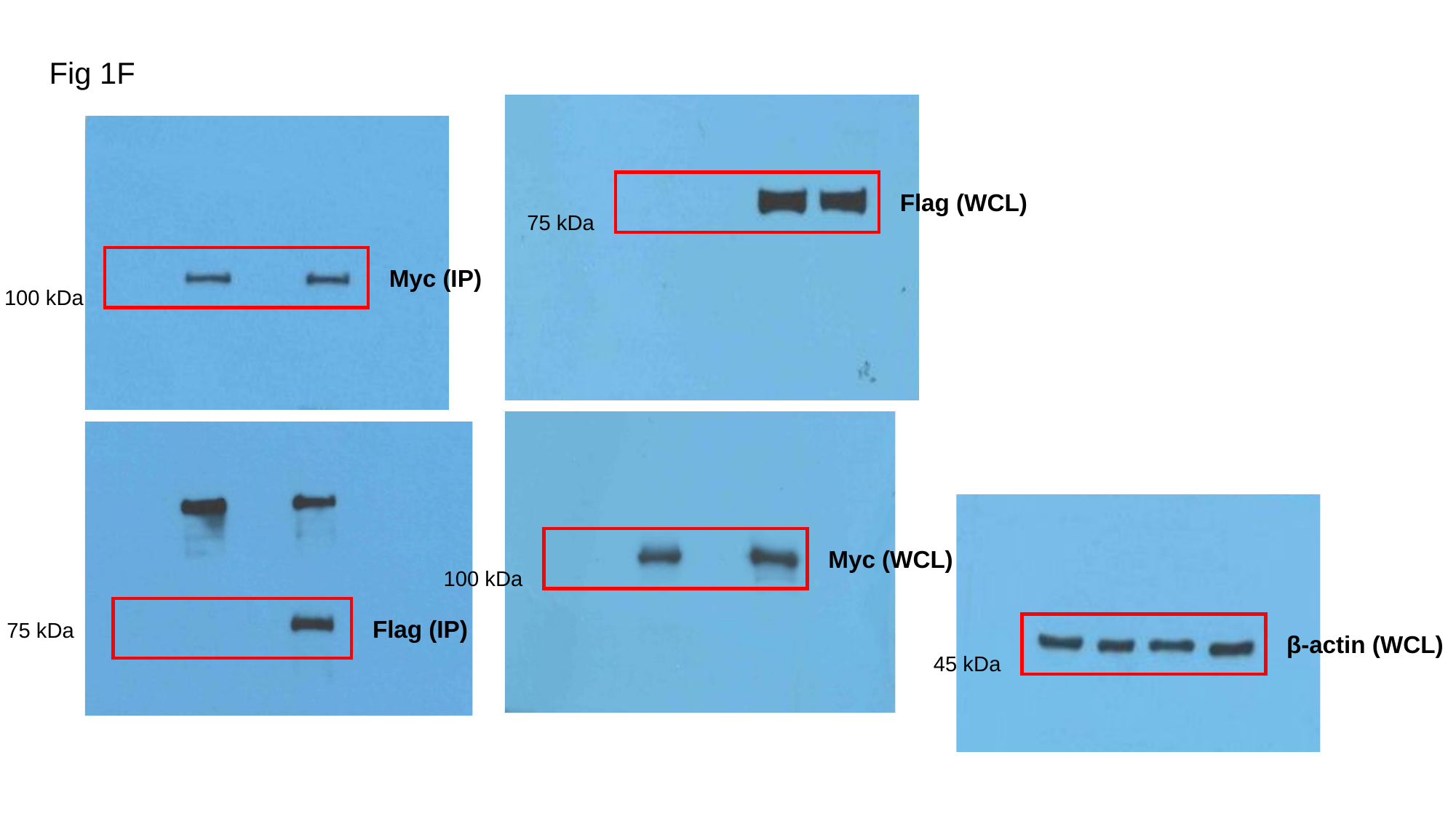

Fig 1F
Flag (WCL)
75 kDa
Myc (IP)
100 kDa
Myc (WCL)
100 kDa
Flag (IP)
75 kDa
β-actin (WCL)
45 kDa

## Slide 4
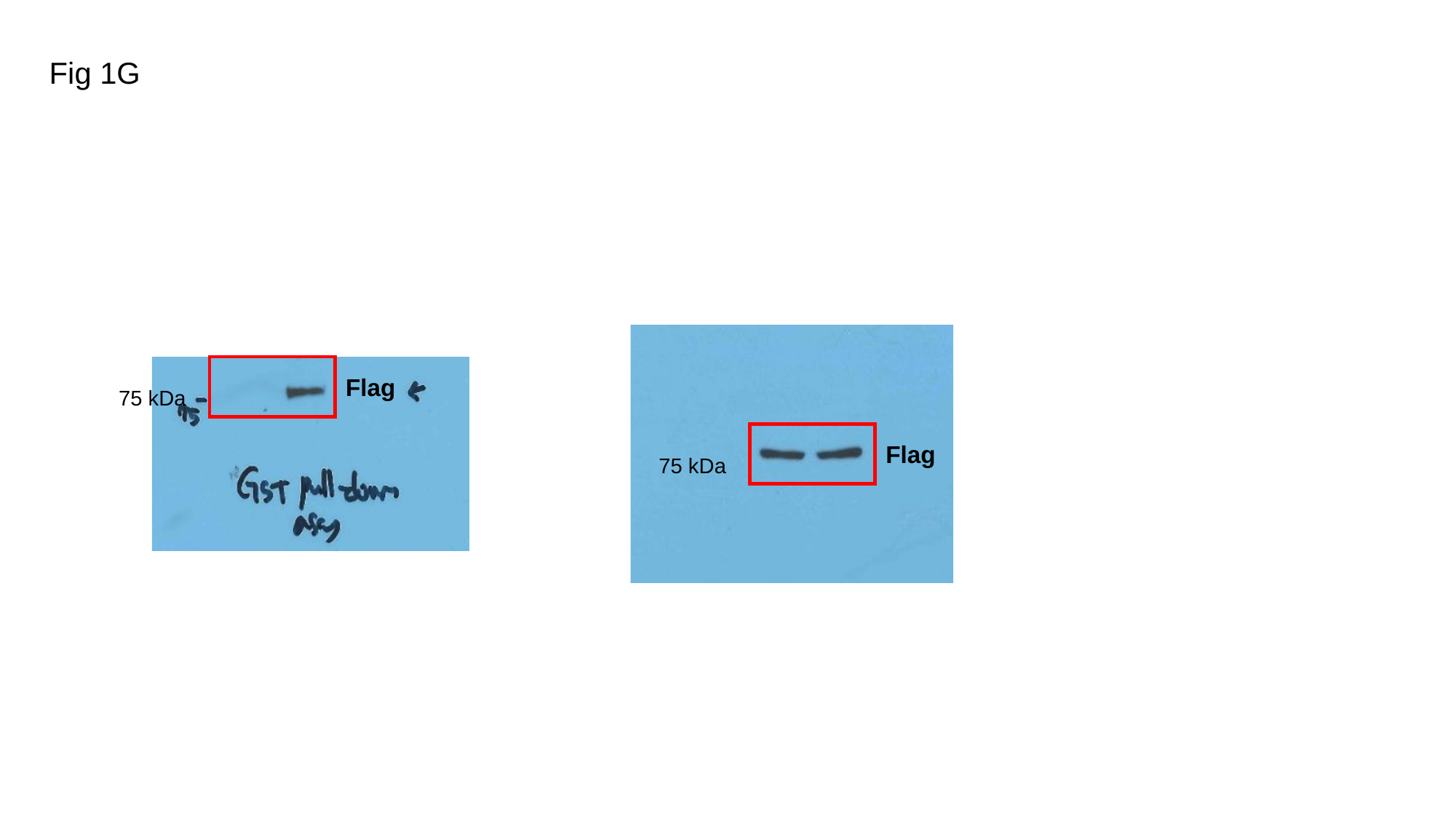

Fig 1G
Flag
75 kDa
Flag
75 kDa

## Slide 5
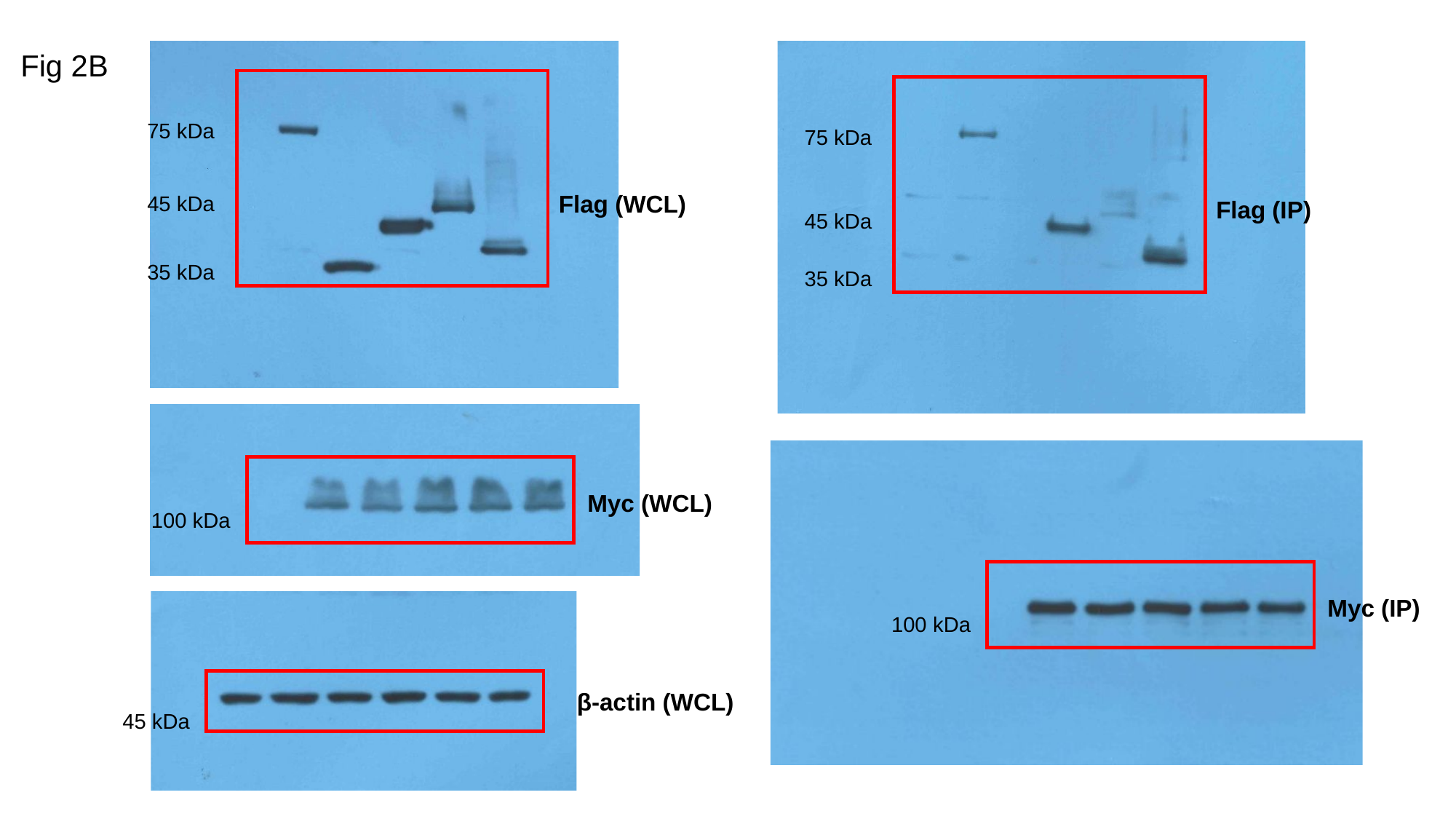

Fig 2B
75 kDa
75 kDa
Flag (WCL)
45 kDa
Flag (IP)
45 kDa
35 kDa
35 kDa
Myc (WCL)
100 kDa
Myc (IP)
100 kDa
β-actin (WCL)
45 kDa

## Slide 6
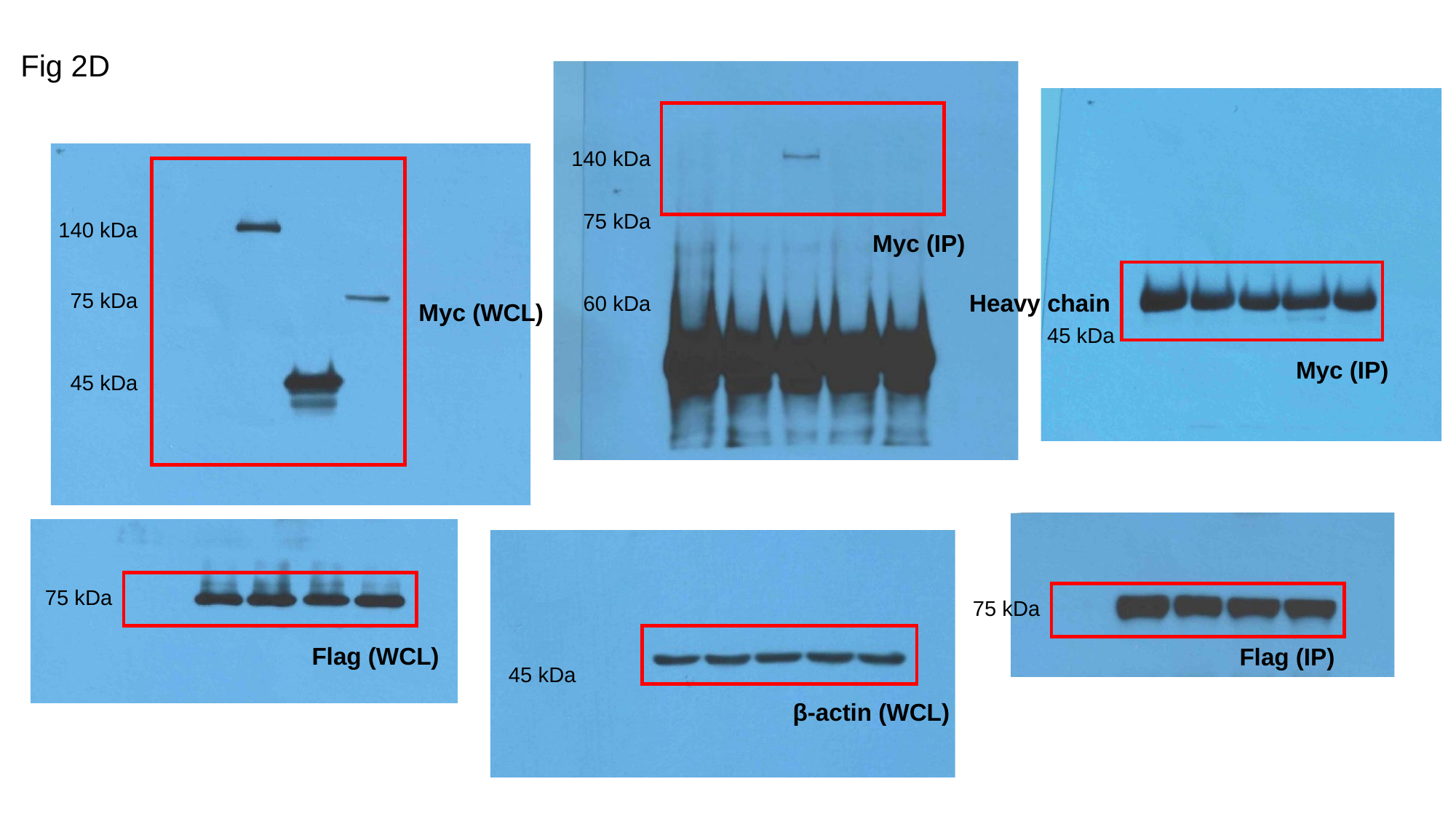

Fig 2D
140 kDa
75 kDa
140 kDa
Myc (IP)
75 kDa
Heavy chain
60 kDa
Myc (WCL)
45 kDa
Myc (IP)
45 kDa
75 kDa
75 kDa
Flag (WCL)
Flag (IP)
45 kDa
β-actin (WCL)

## Slide 7
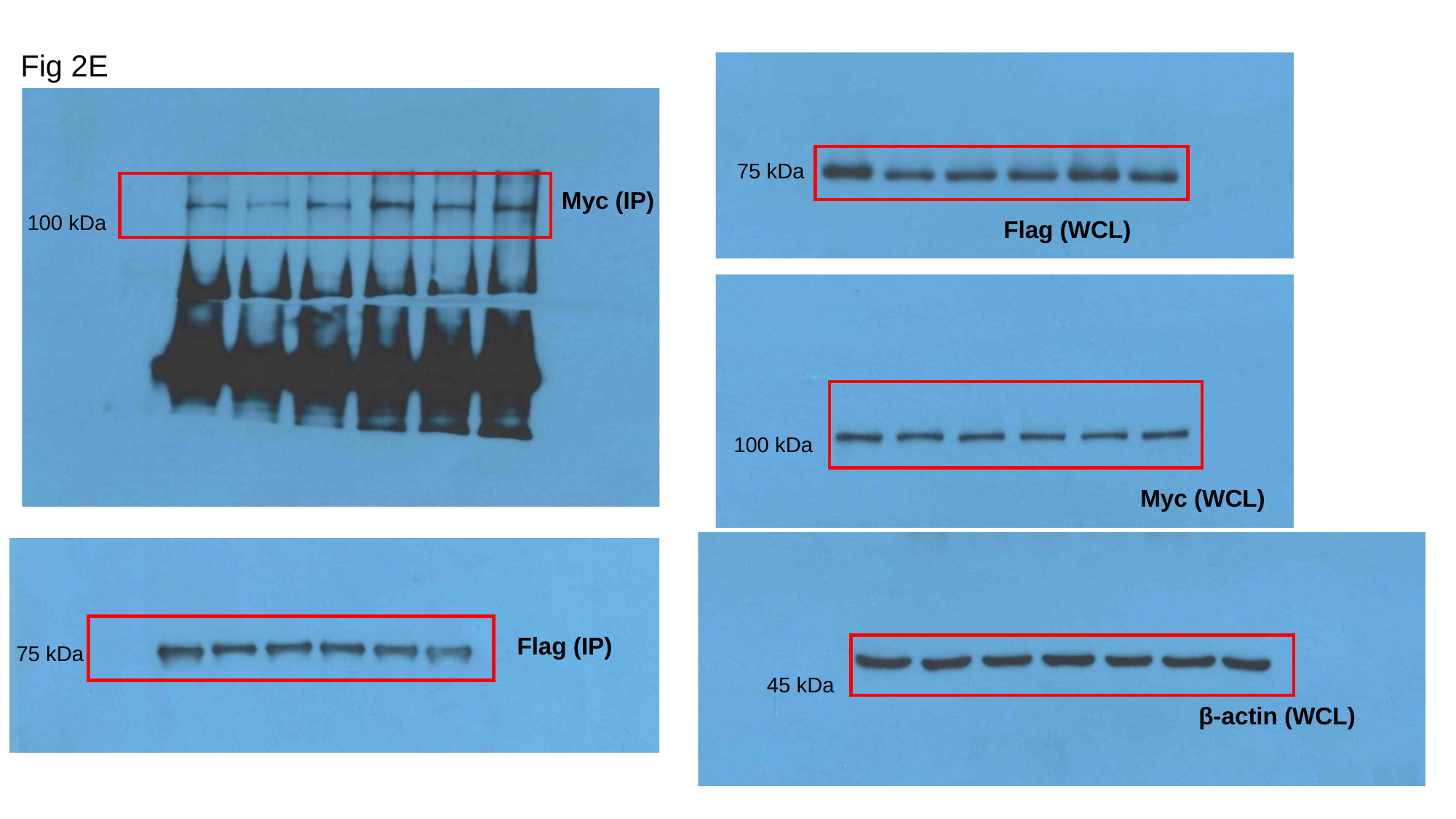

Fig 2E
75 kDa
Myc (IP)
100 kDa
Flag (WCL)
100 kDa
Myc (WCL)
Flag (IP)
75 kDa
45 kDa
β-actin (WCL)

## Slide 8
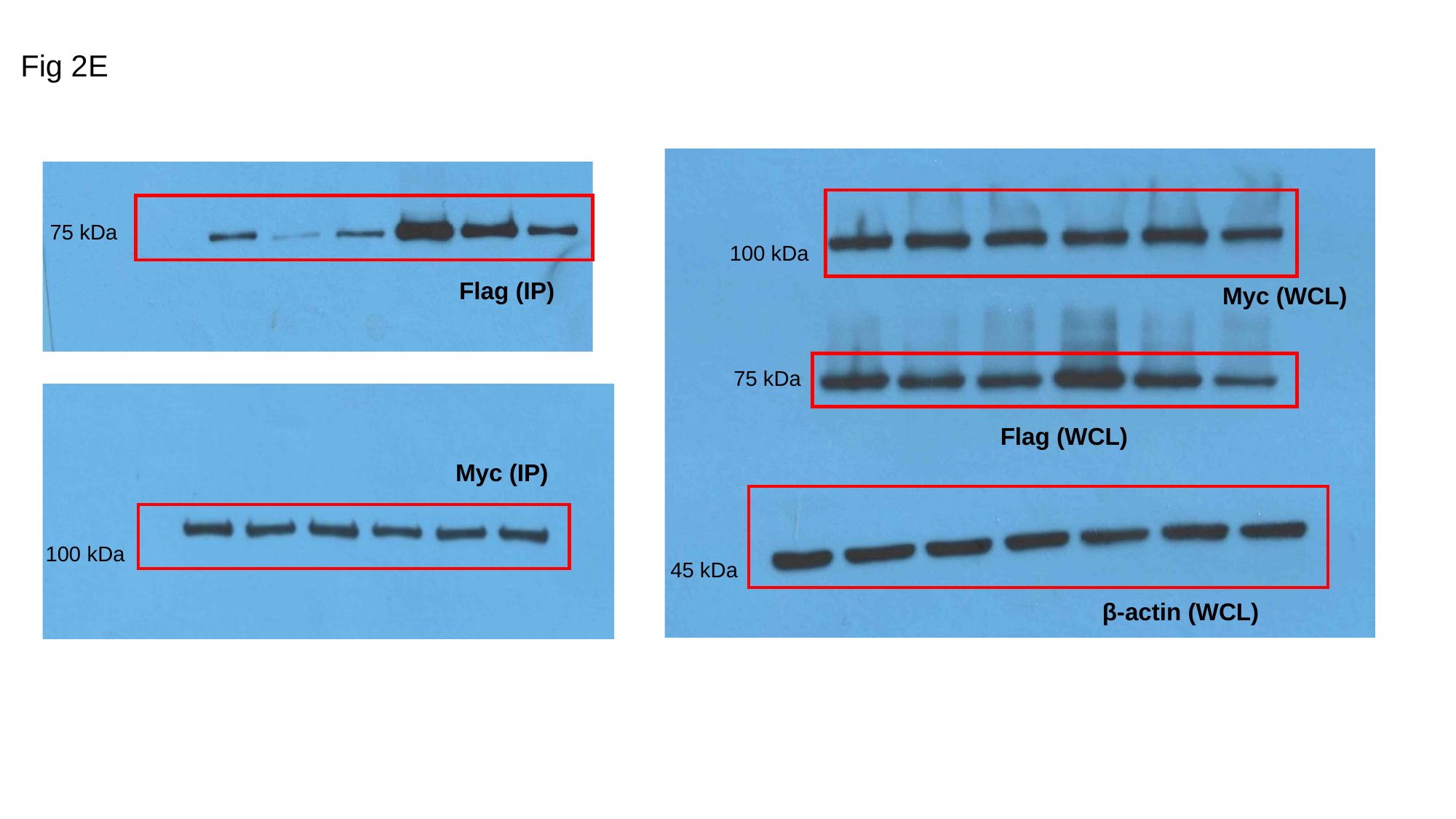

Fig 2E
75 kDa
100 kDa
Flag (IP)
Myc (WCL)
75 kDa
Flag (WCL)
Myc (IP)
100 kDa
45 kDa
β-actin (WCL)

## Slide 9
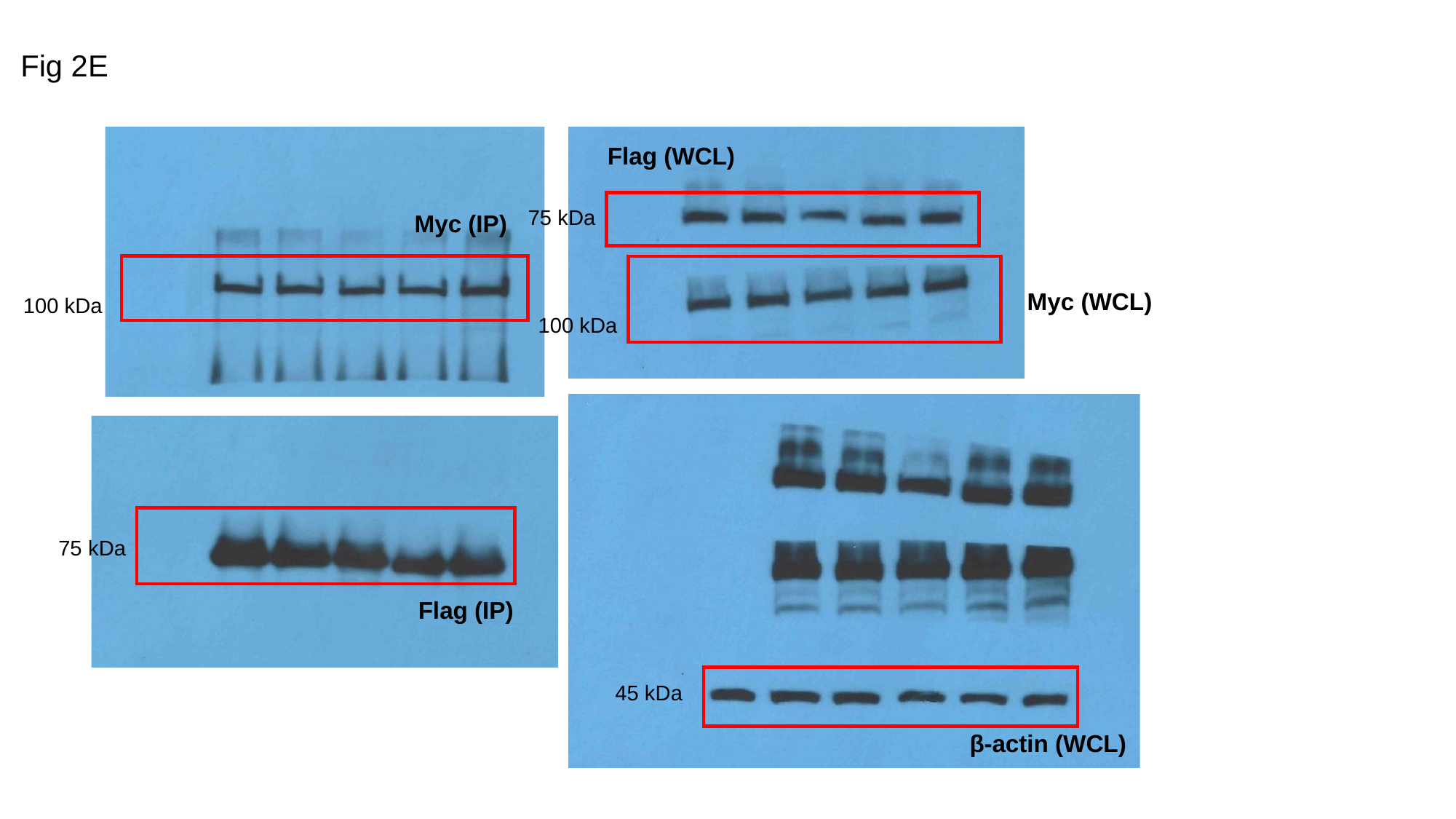

Fig 2E
Flag (WCL)
75 kDa
Myc (IP)
Myc (WCL)
100 kDa
100 kDa
75 kDa
Flag (IP)
45 kDa
β-actin (WCL)

## Slide 10
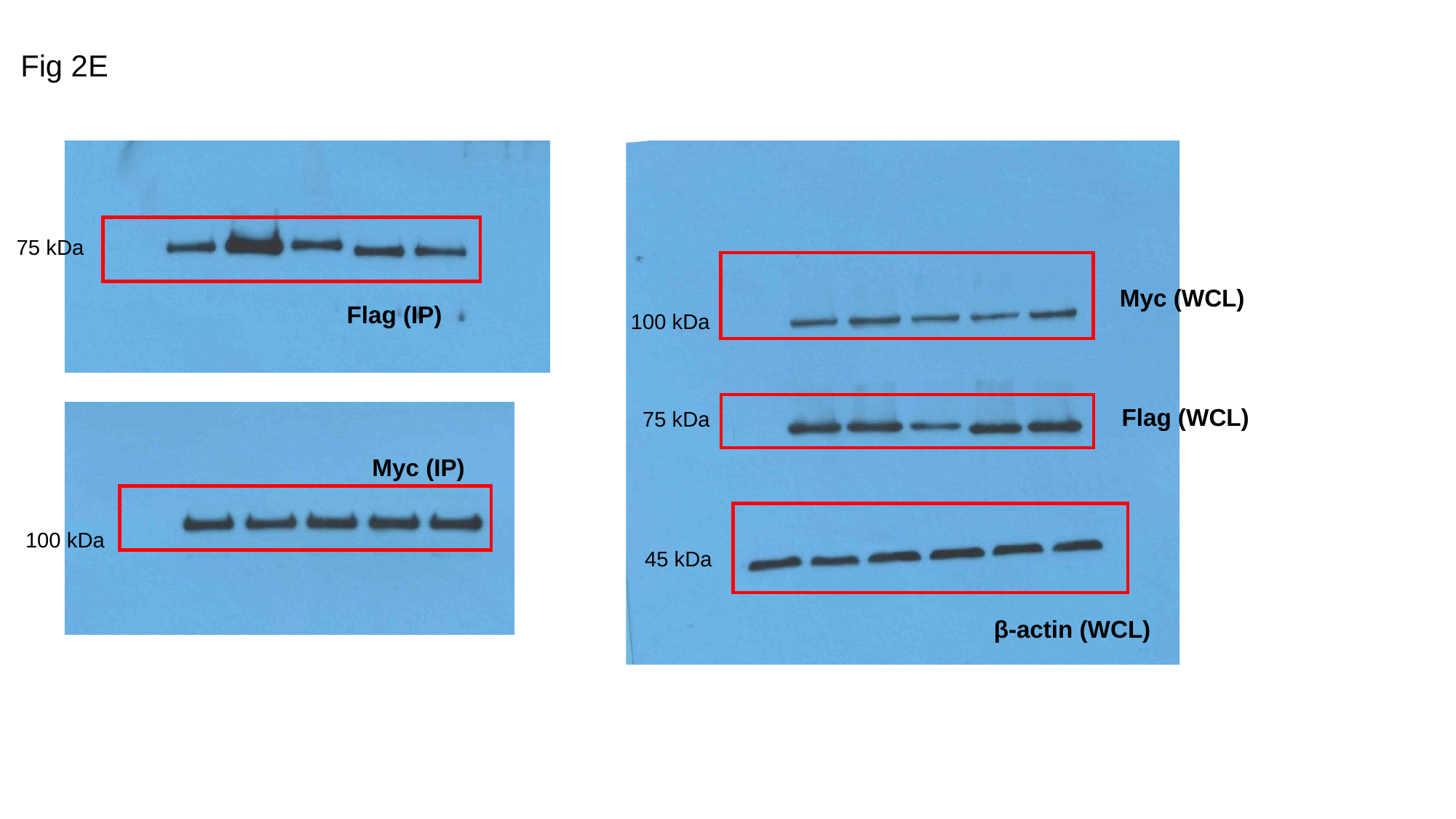

Fig 2E
75 kDa
Myc (WCL)
Flag (IP)
100 kDa
Flag (WCL)
75 kDa
Myc (IP)
100 kDa
45 kDa
β-actin (WCL)

## Slide 11
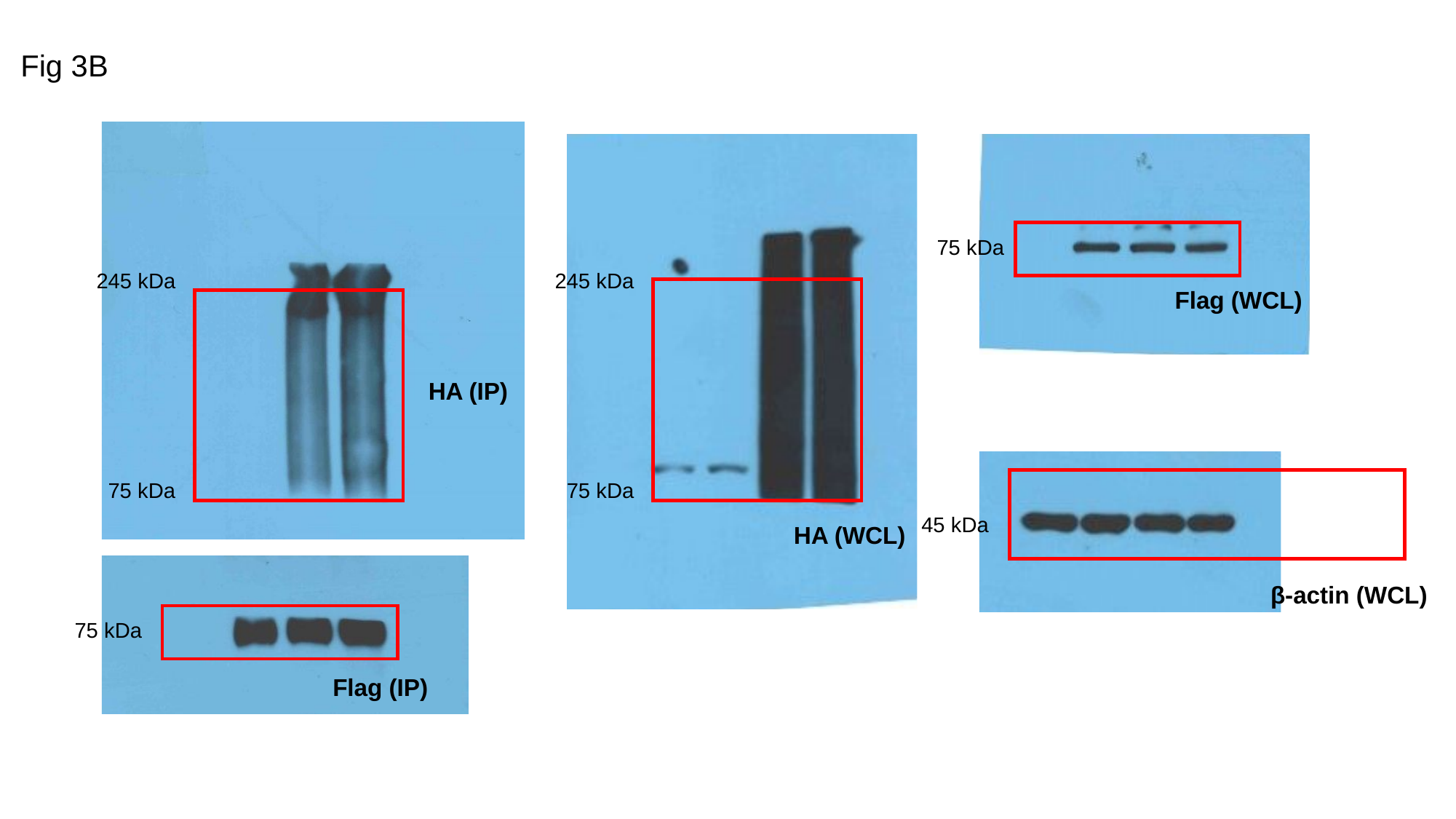

Fig 3B
75 kDa
245 kDa
245 kDa
Flag (WCL)
HA (IP)
75 kDa
75 kDa
45 kDa
HA (WCL)
β-actin (WCL)
75 kDa
Flag (IP)

## Slide 12
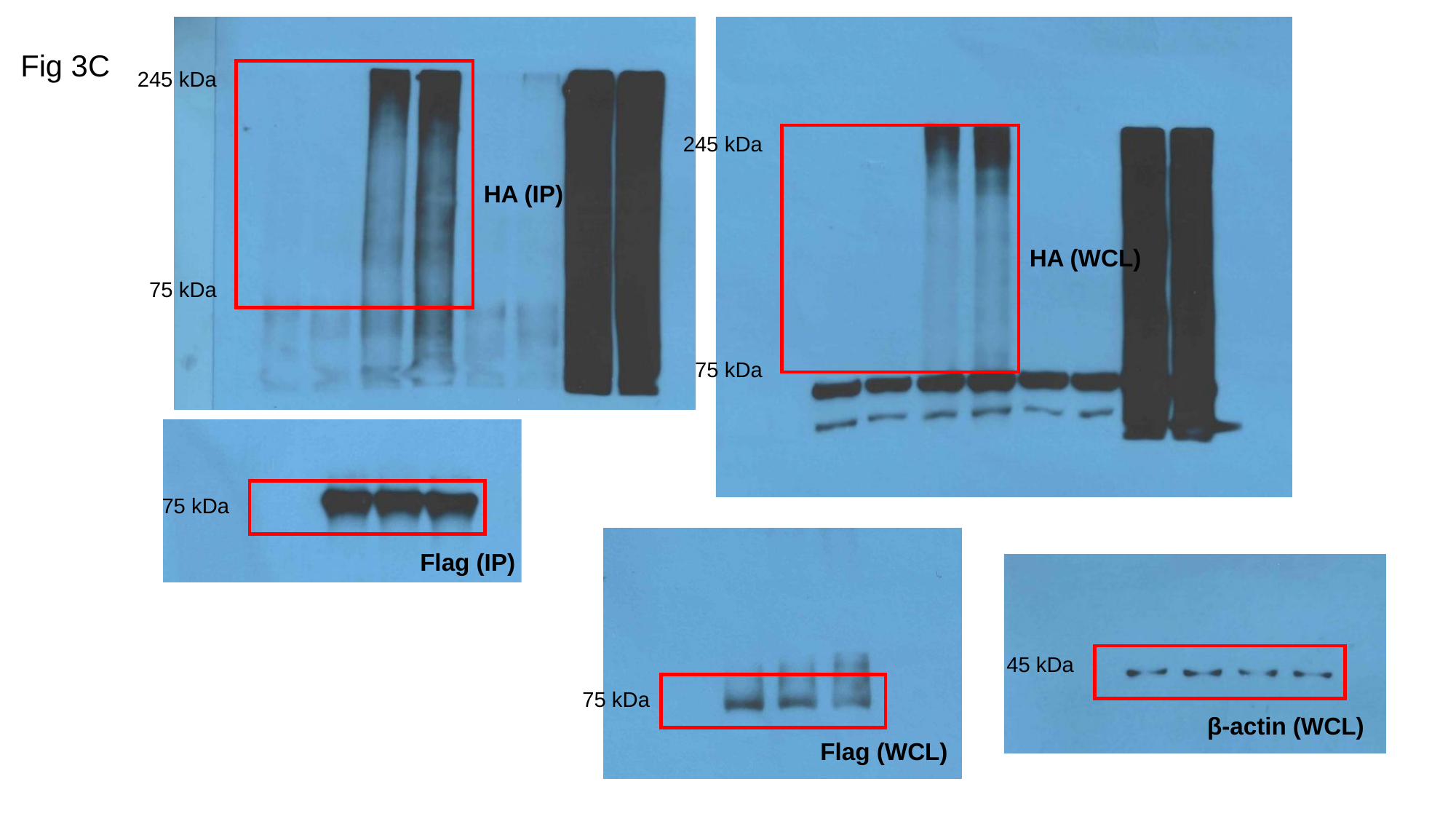

Fig 3C
245 kDa
245 kDa
HA (IP)
HA (WCL)
75 kDa
75 kDa
75 kDa
Flag (IP)
45 kDa
75 kDa
β-actin (WCL)
Flag (WCL)

## Slide 13
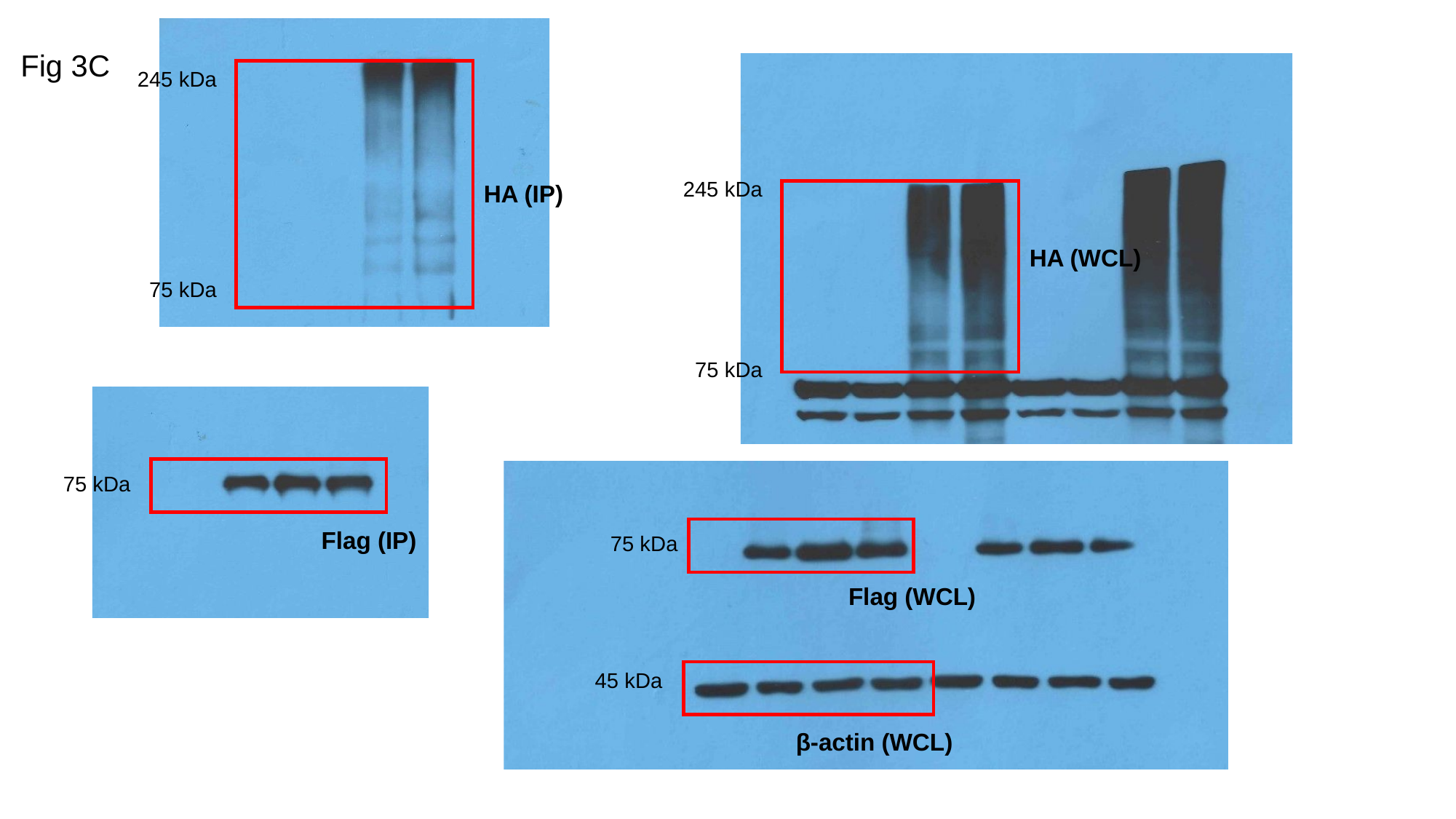

Fig 3C
245 kDa
245 kDa
HA (IP)
HA (WCL)
75 kDa
75 kDa
75 kDa
Flag (IP)
75 kDa
Flag (WCL)
45 kDa
β-actin (WCL)

## Slide 14
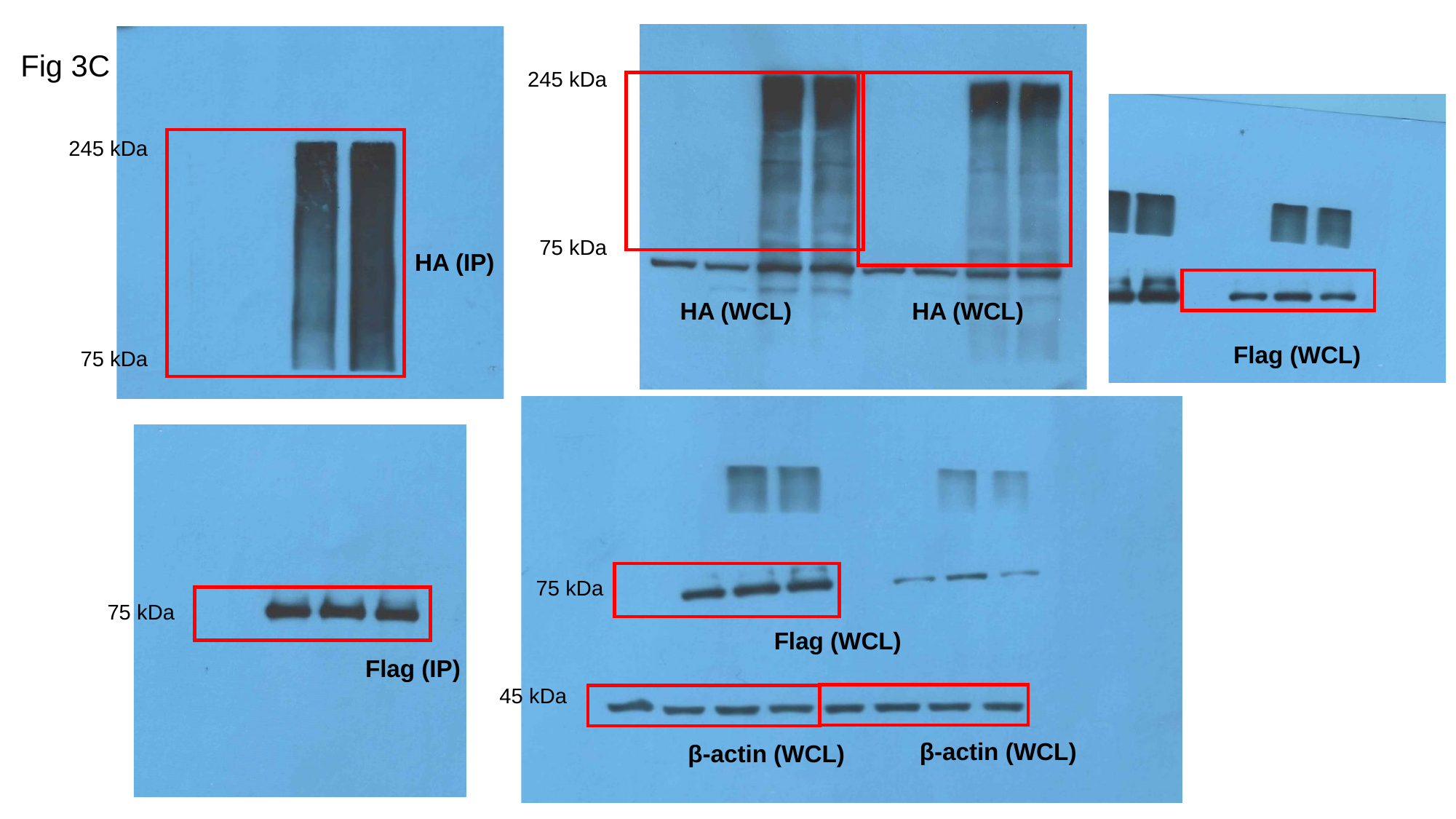

Fig 3C
245 kDa
245 kDa
75 kDa
HA (IP)
HA (WCL)
HA (WCL)
Flag (WCL)
75 kDa
75 kDa
75 kDa
Flag (WCL)
Flag (IP)
45 kDa
β-actin (WCL)
β-actin (WCL)

## Slide 15
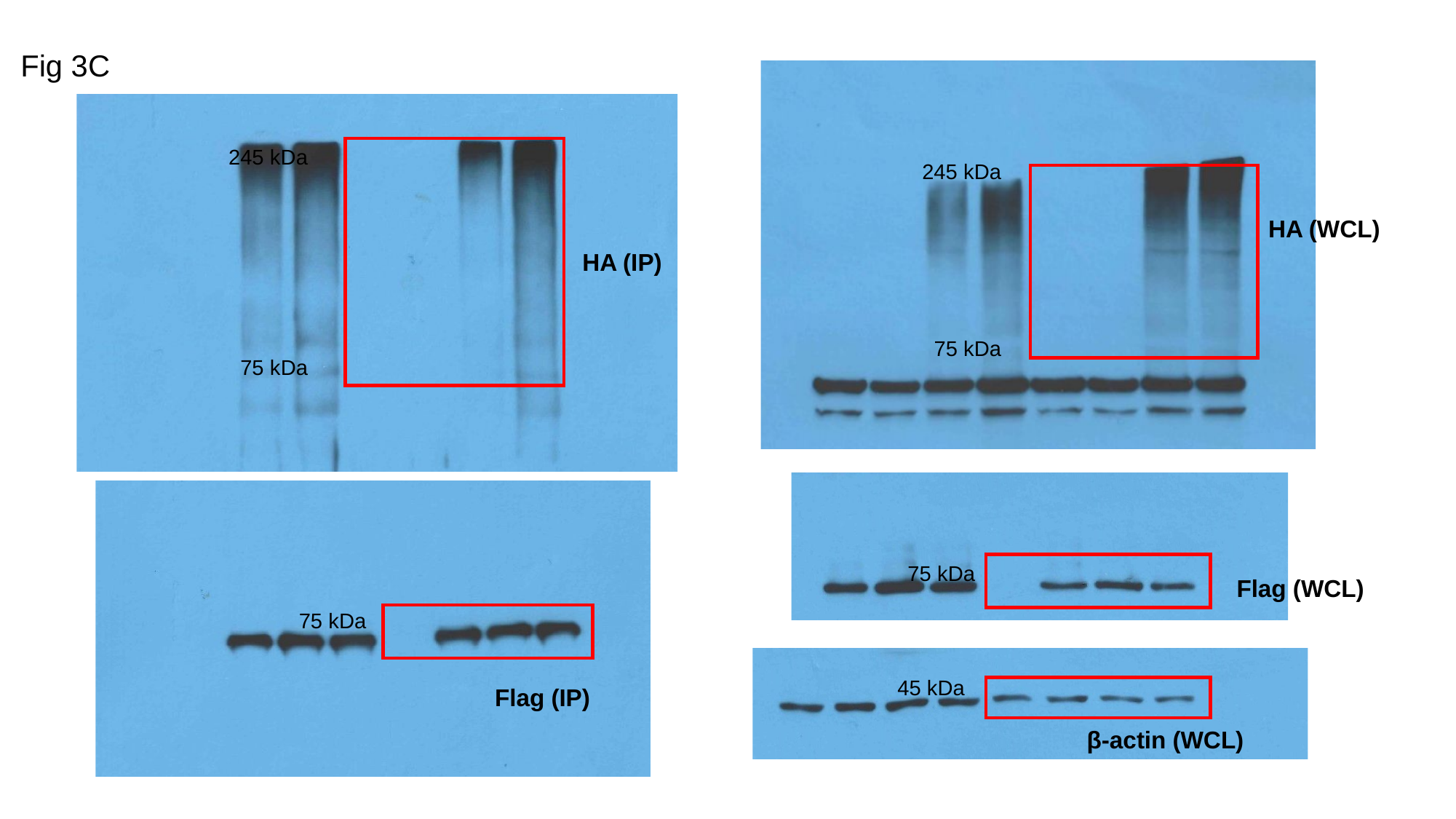

Fig 3C
245 kDa
245 kDa
HA (WCL)
HA (IP)
75 kDa
75 kDa
75 kDa
Flag (WCL)
75 kDa
45 kDa
Flag (IP)
β-actin (WCL)

## Slide 16
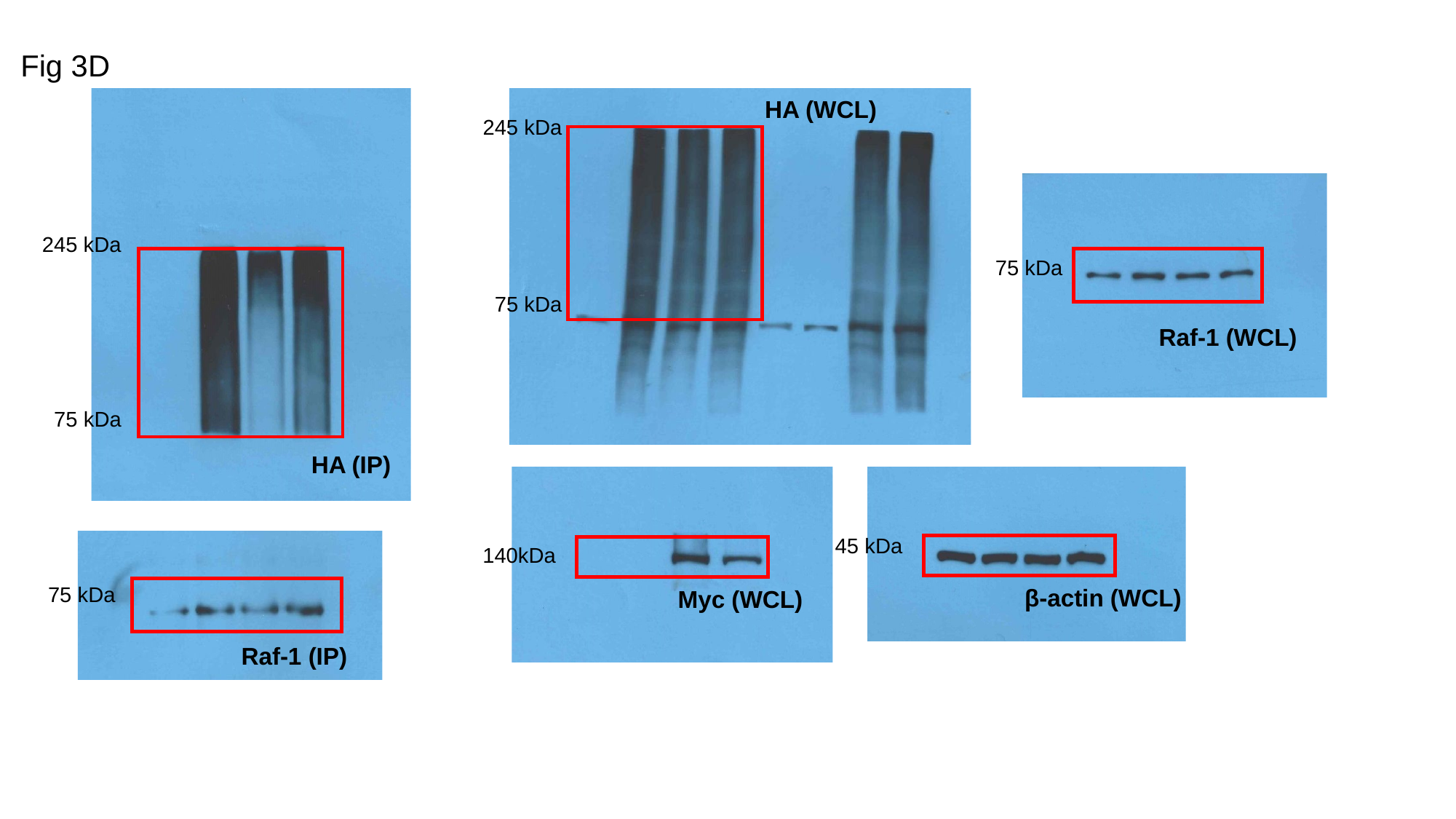

Fig 3D
HA (WCL)
245 kDa
245 kDa
75 kDa
75 kDa
Raf-1 (WCL)
75 kDa
HA (IP)
45 kDa
140kDa
75 kDa
β-actin (WCL)
Myc (WCL)
Raf-1 (IP)

## Slide 17
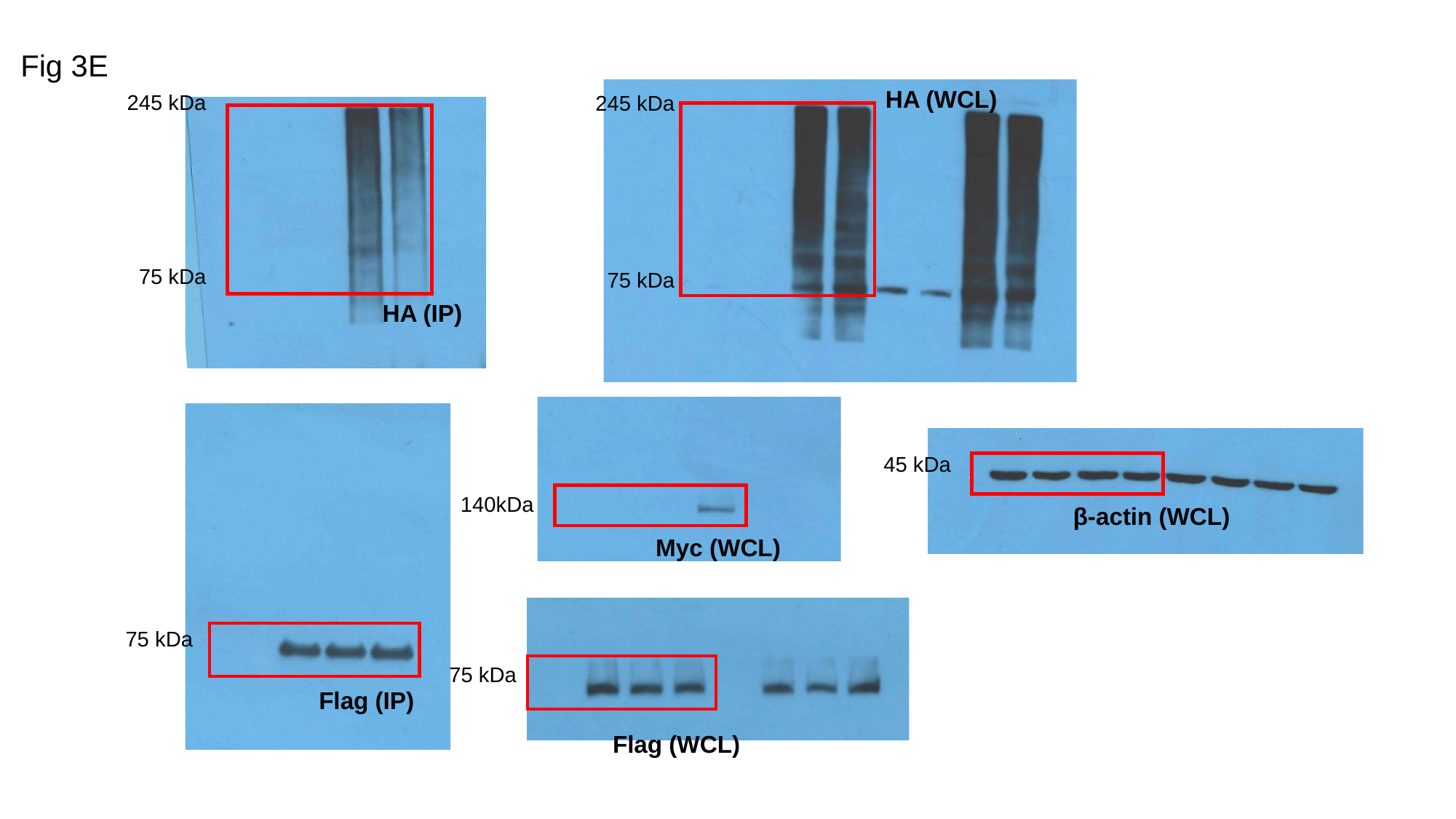

Fig 3E
HA (WCL)
245 kDa
245 kDa
75 kDa
75 kDa
HA (IP)
45 kDa
140kDa
β-actin (WCL)
Myc (WCL)
75 kDa
75 kDa
Flag (IP)
Flag (WCL)

## Slide 18
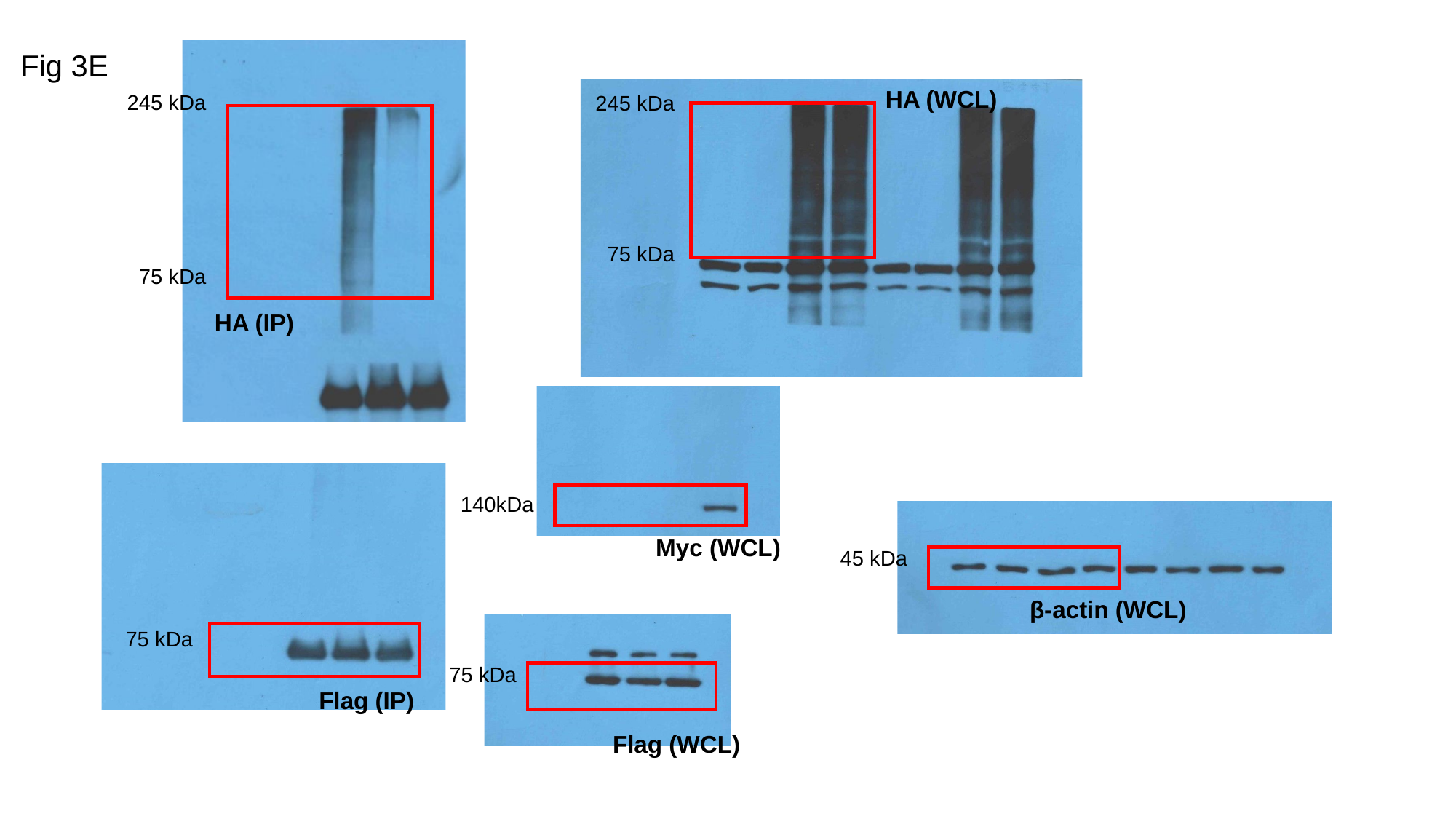

Fig 3E
HA (WCL)
245 kDa
245 kDa
75 kDa
75 kDa
HA (IP)
140kDa
Myc (WCL)
45 kDa
β-actin (WCL)
75 kDa
75 kDa
Flag (IP)
Flag (WCL)

## Slide 19
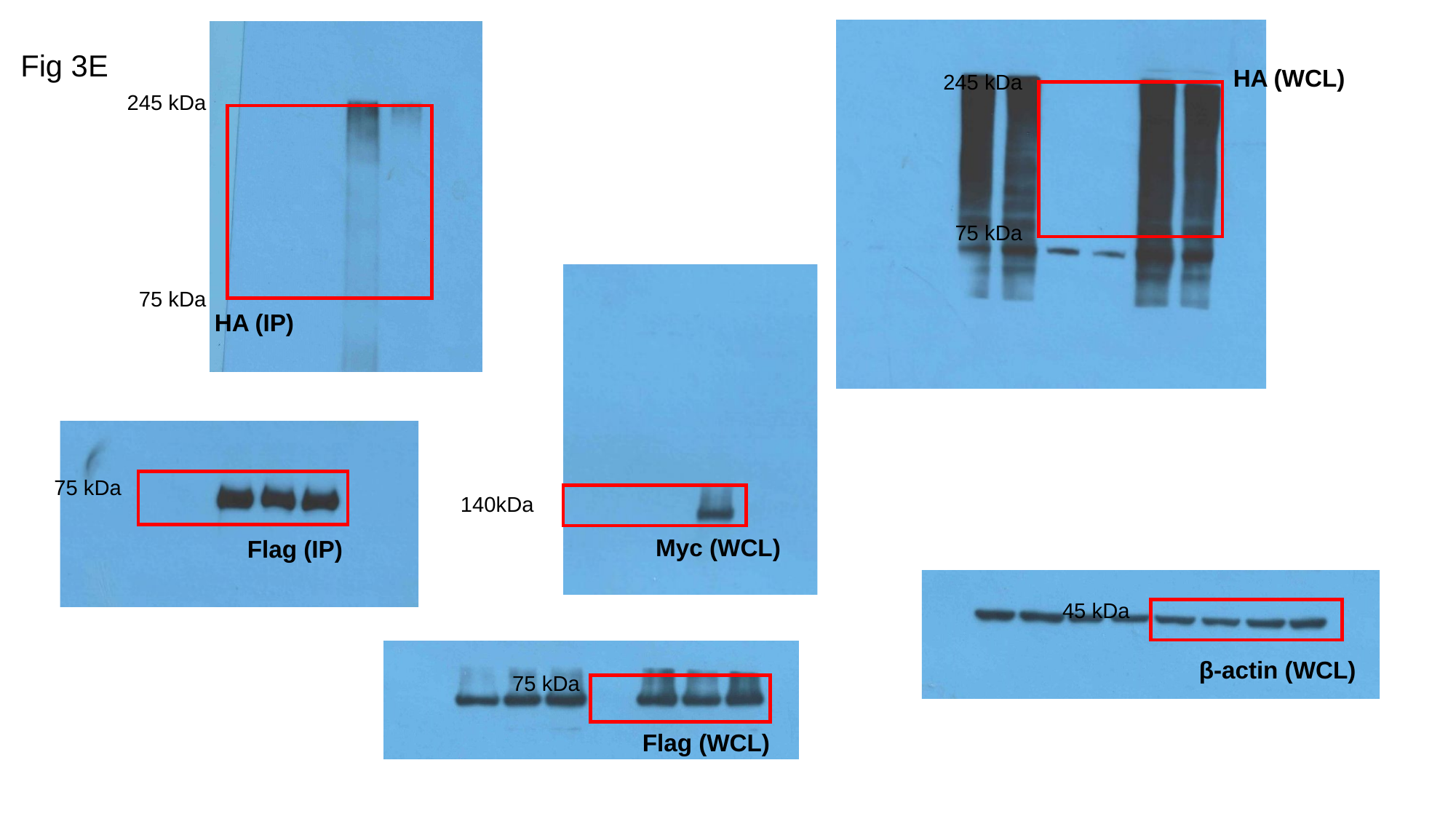

Fig 3E
HA (WCL)
245 kDa
245 kDa
75 kDa
75 kDa
HA (IP)
75 kDa
140kDa
Myc (WCL)
Flag (IP)
45 kDa
β-actin (WCL)
75 kDa
Flag (WCL)

## Slide 20
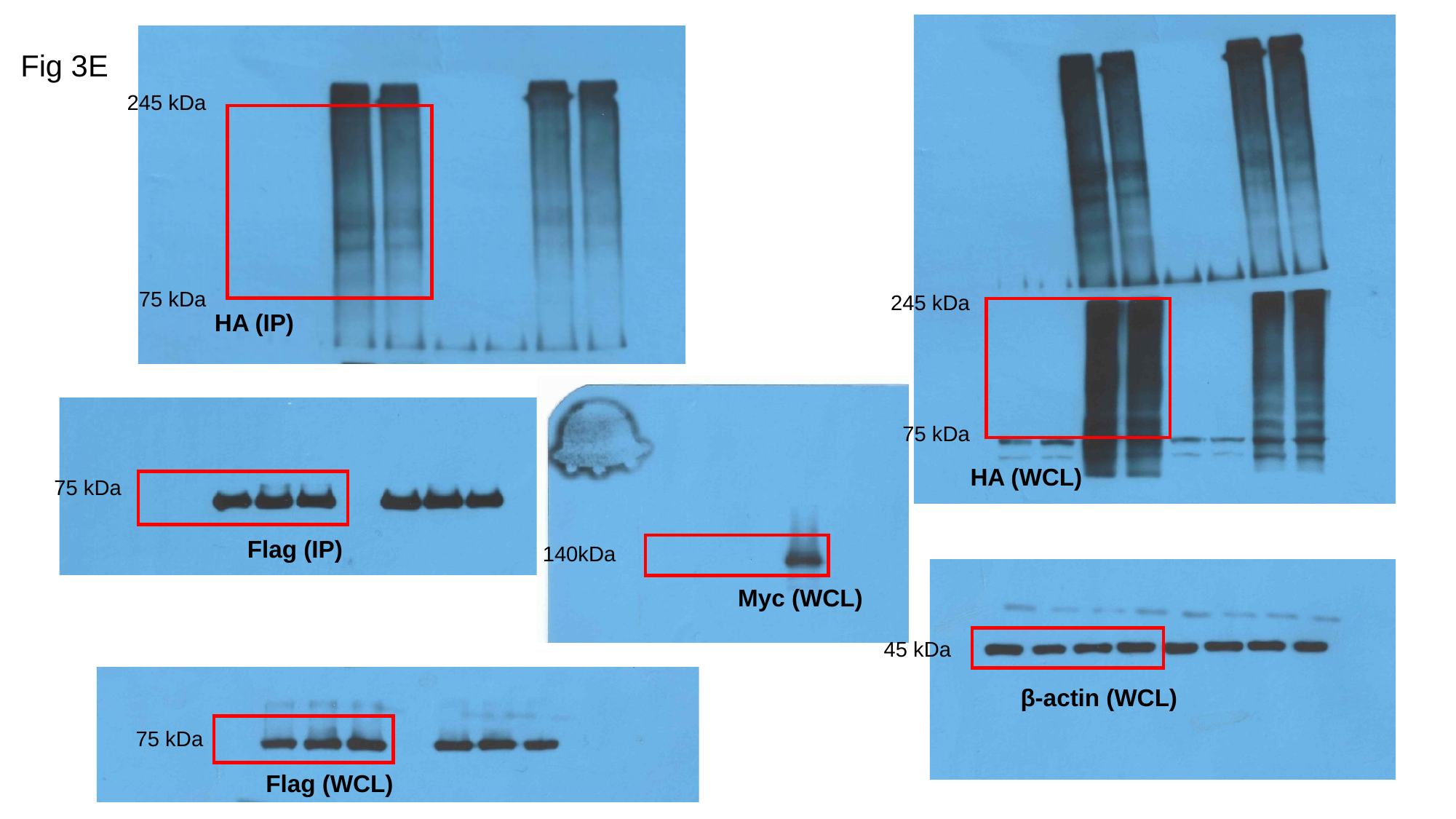

Fig 3E
245 kDa
75 kDa
245 kDa
HA (IP)
75 kDa
HA (WCL)
75 kDa
Flag (IP)
140kDa
Myc (WCL)
45 kDa
β-actin (WCL)
75 kDa
Flag (WCL)

## Slide 21
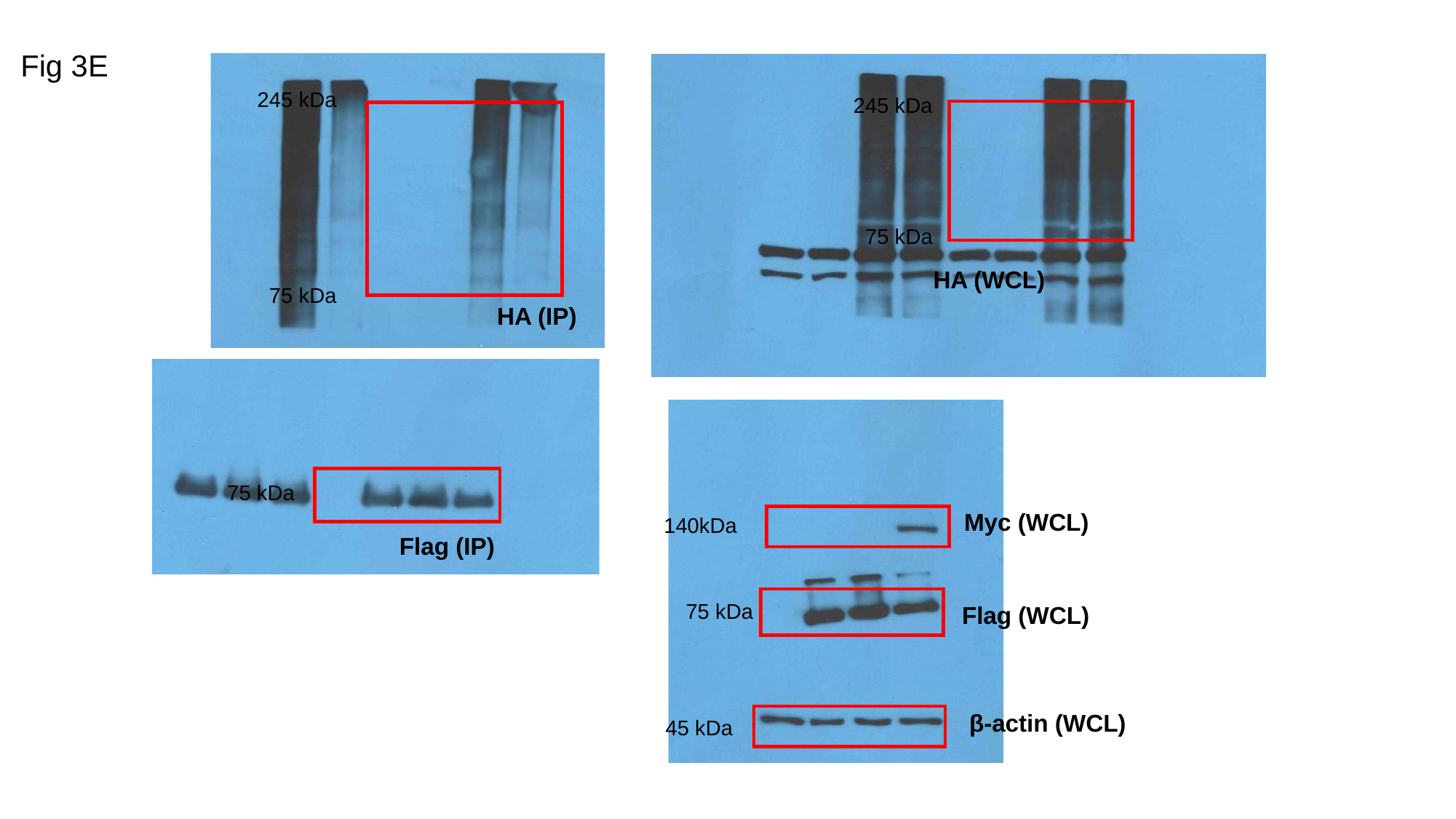

Fig 3E
245 kDa
245 kDa
75 kDa
HA (WCL)
75 kDa
HA (IP)
75 kDa
Myc (WCL)
140kDa
Flag (IP)
75 kDa
Flag (WCL)
β-actin (WCL)
45 kDa

## Slide 22
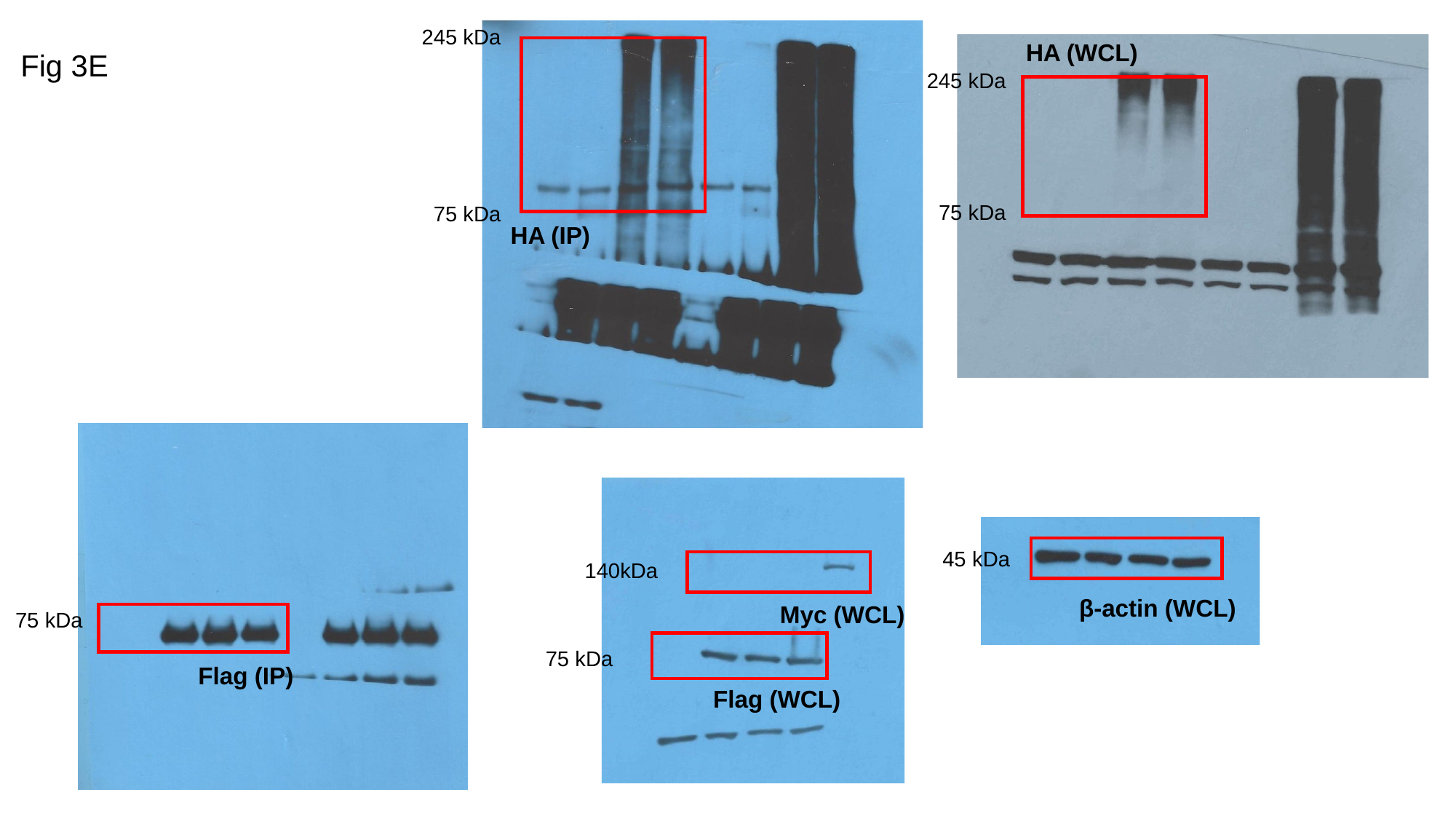

245 kDa
HA (WCL)
Fig 3E
245 kDa
75 kDa
75 kDa
HA (IP)
45 kDa
140kDa
β-actin (WCL)
Myc (WCL)
75 kDa
75 kDa
Flag (IP)
Flag (WCL)

## Slide 23
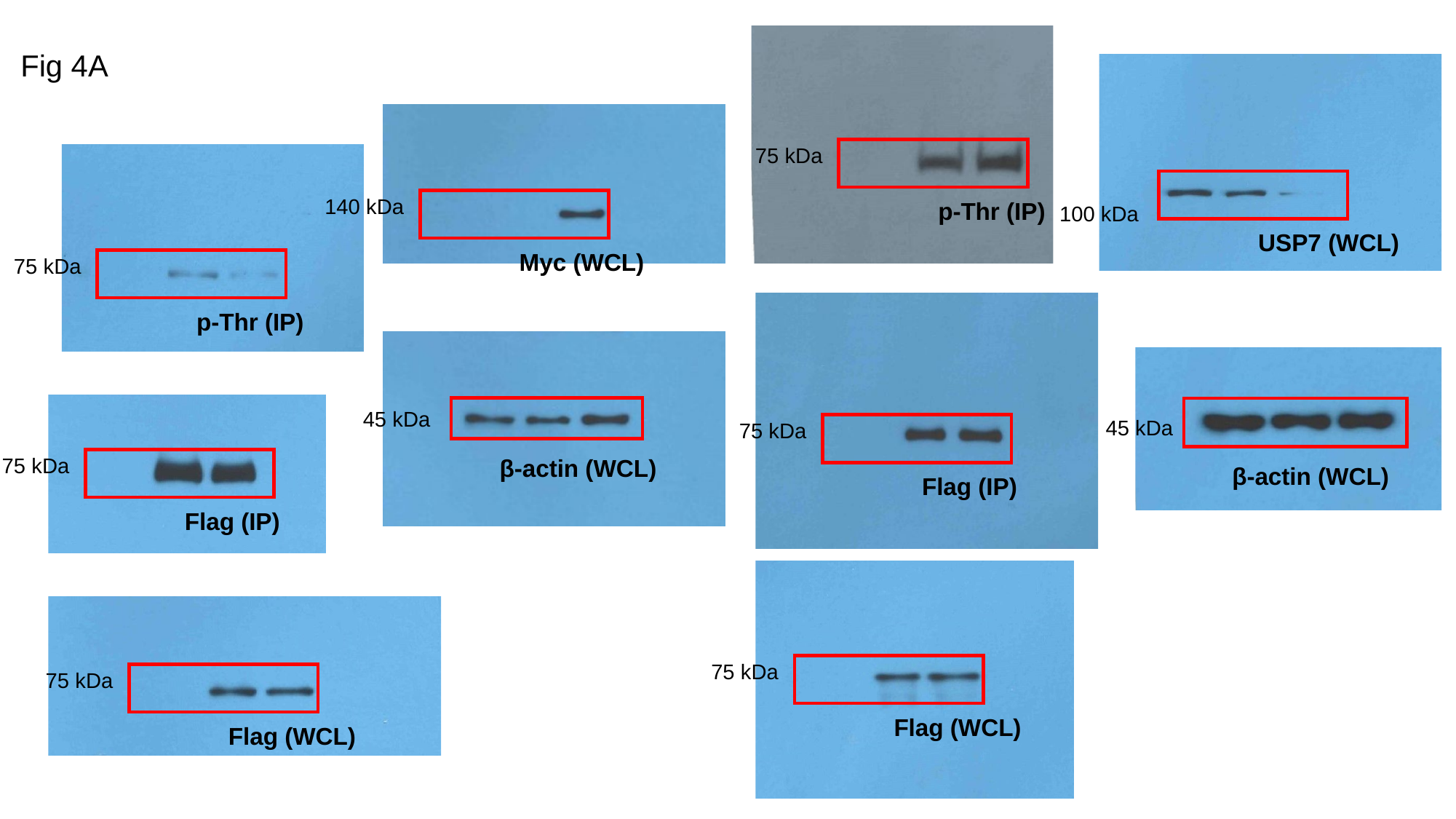

Fig 4A
75 kDa
140 kDa
p-Thr (IP)
100 kDa
USP7 (WCL)
Myc (WCL)
75 kDa
p-Thr (IP)
45 kDa
45 kDa
75 kDa
75 kDa
β-actin (WCL)
β-actin (WCL)
Flag (IP)
Flag (IP)
75 kDa
75 kDa
Flag (WCL)
Flag (WCL)

## Slide 24
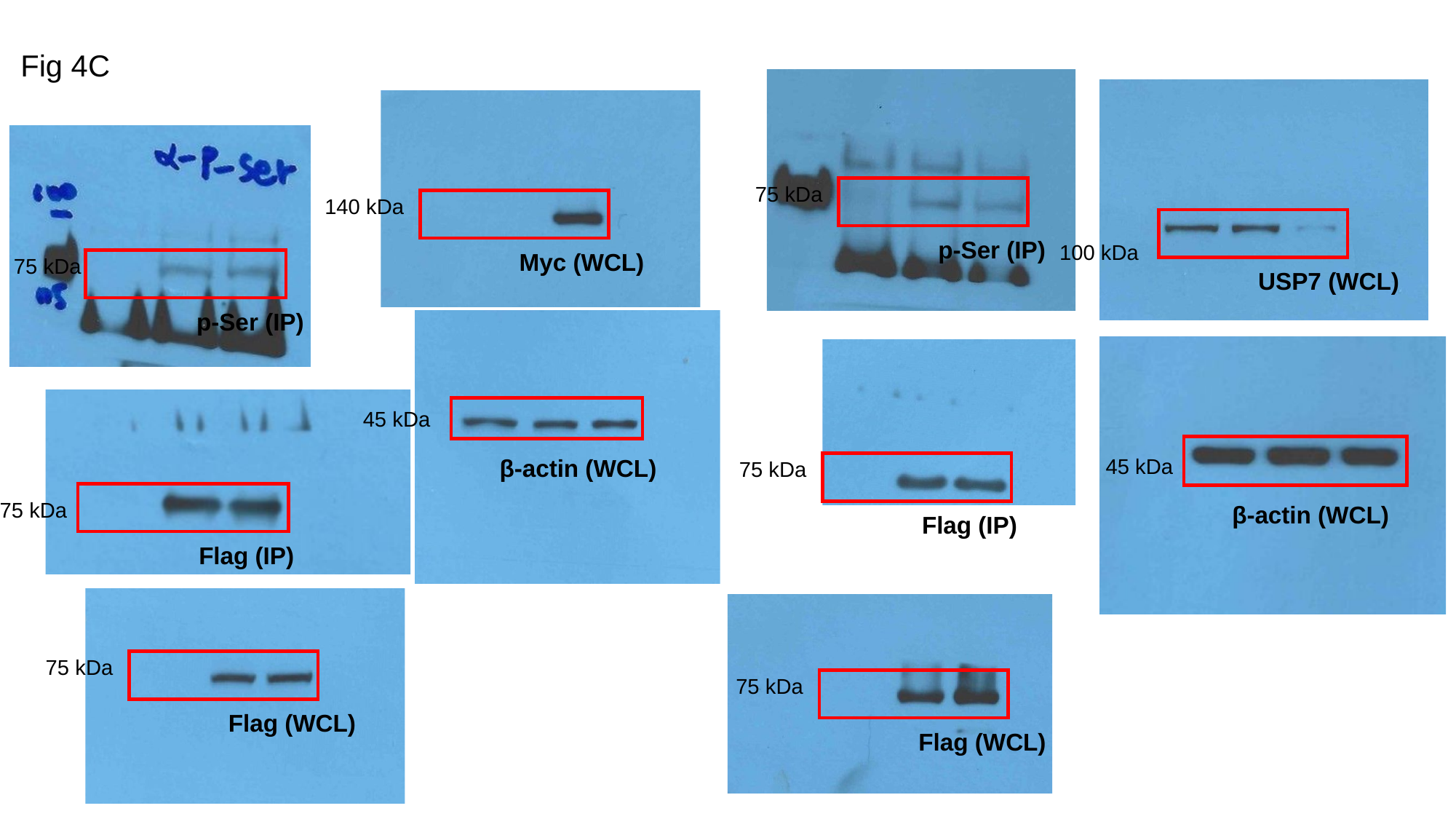

Fig 4C
75 kDa
140 kDa
p-Ser (IP)
100 kDa
Myc (WCL)
75 kDa
USP7 (WCL)
p-Ser (IP)
45 kDa
45 kDa
β-actin (WCL)
75 kDa
75 kDa
β-actin (WCL)
Flag (IP)
Flag (IP)
75 kDa
75 kDa
Flag (WCL)
Flag (WCL)

## Slide 25
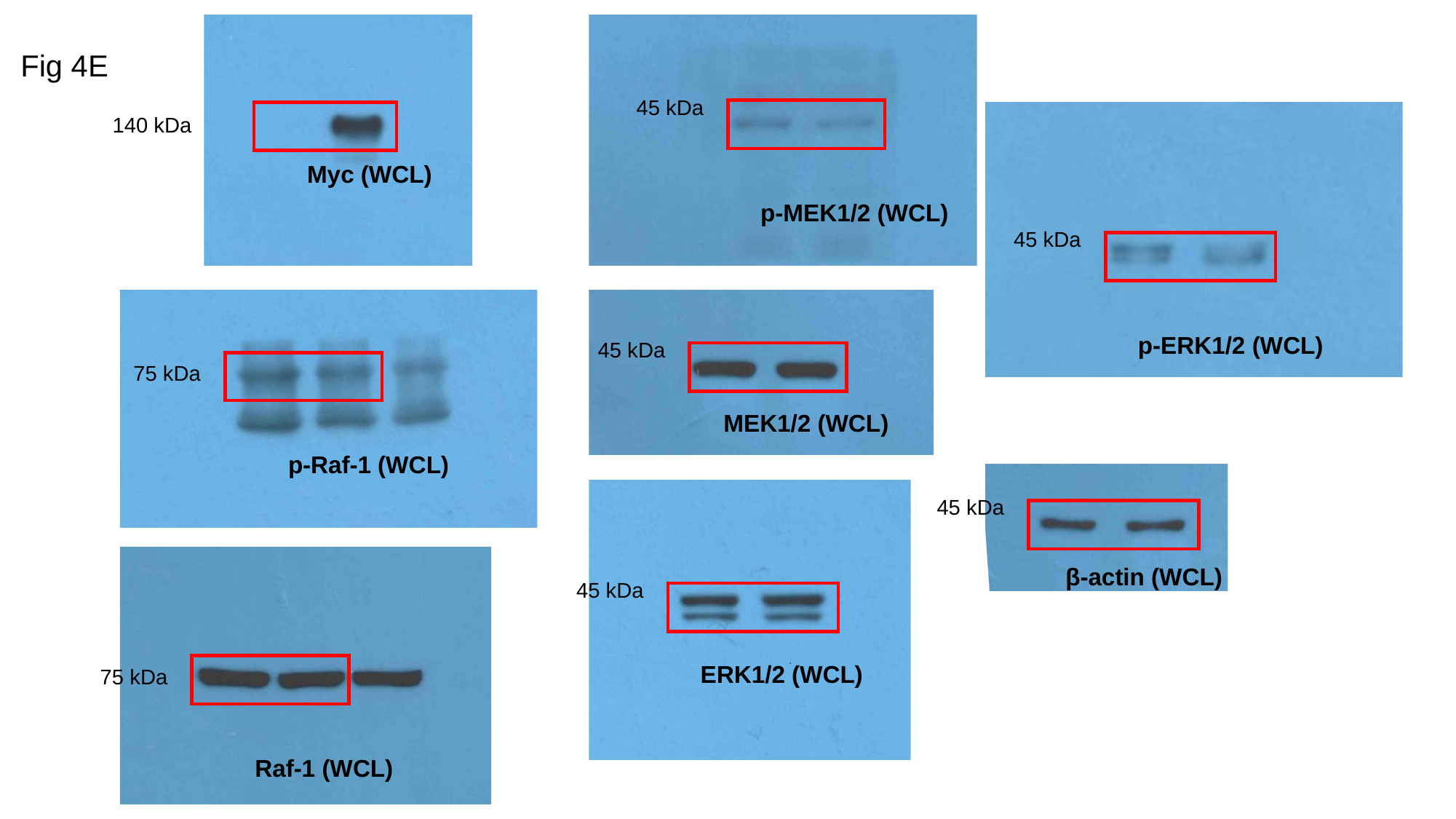

Fig 4E
45 kDa
140 kDa
Myc (WCL)
p-MEK1/2 (WCL)
45 kDa
p-ERK1/2 (WCL)
45 kDa
75 kDa
MEK1/2 (WCL)
p-Raf-1 (WCL)
45 kDa
β-actin (WCL)
45 kDa
ERK1/2 (WCL)
75 kDa
Raf-1 (WCL)

## Slide 26
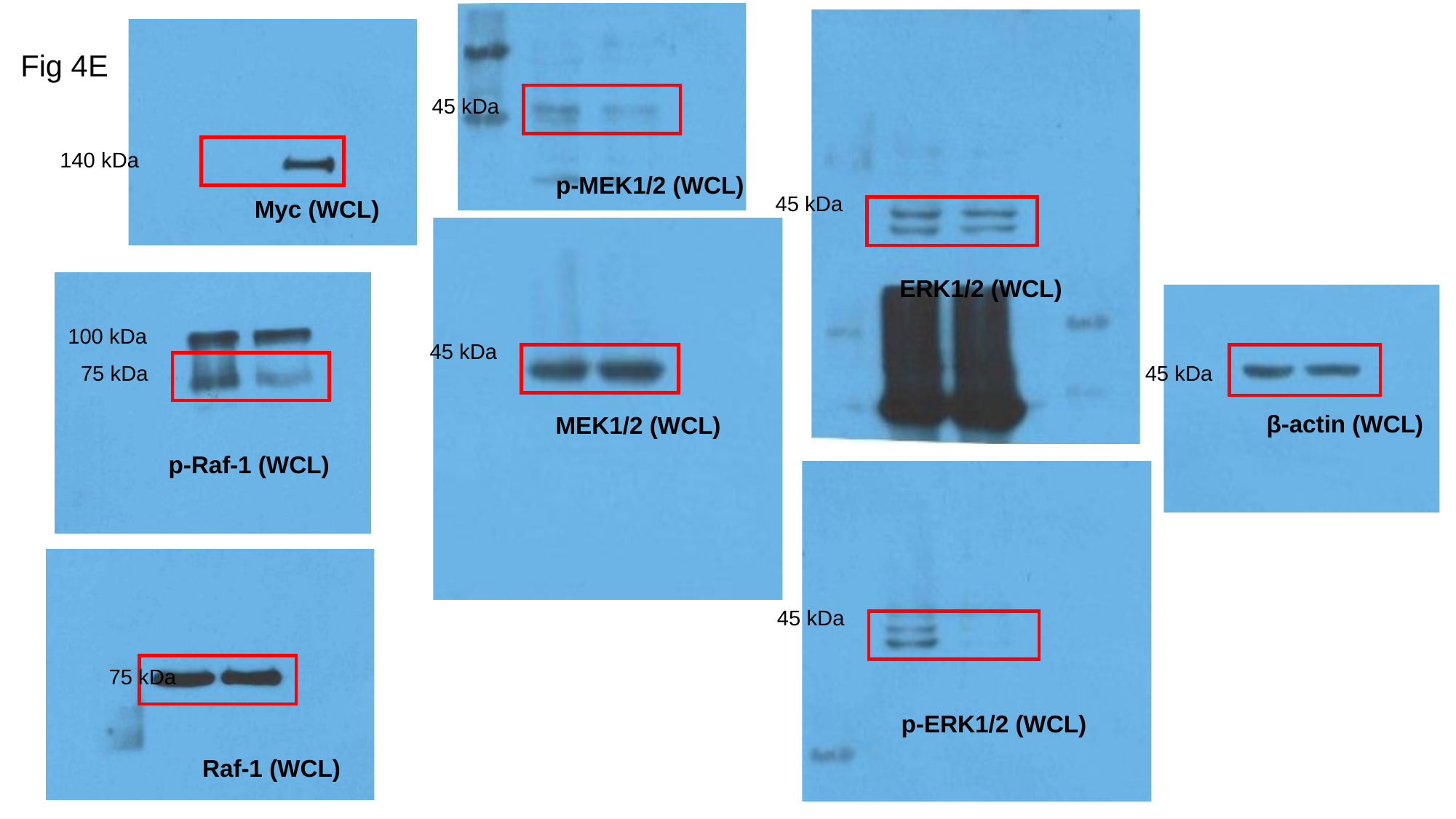

Fig 4E
45 kDa
140 kDa
p-MEK1/2 (WCL)
45 kDa
Myc (WCL)
ERK1/2 (WCL)
100 kDa
45 kDa
75 kDa
45 kDa
β-actin (WCL)
MEK1/2 (WCL)
p-Raf-1 (WCL)
45 kDa
75 kDa
p-ERK1/2 (WCL)
Raf-1 (WCL)

## Slide 27
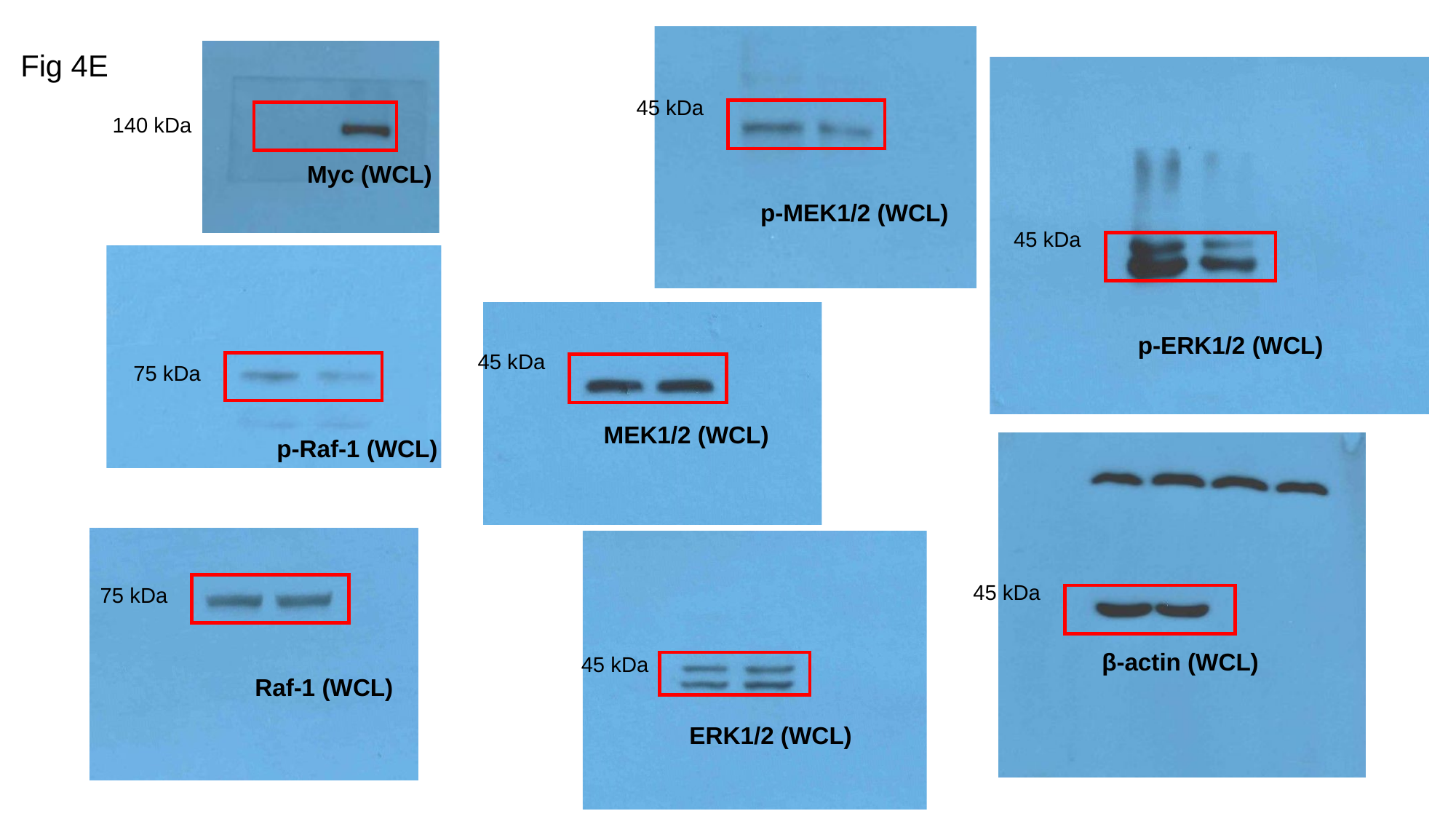

Fig 4E
45 kDa
140 kDa
Myc (WCL)
p-MEK1/2 (WCL)
45 kDa
p-ERK1/2 (WCL)
45 kDa
75 kDa
MEK1/2 (WCL)
p-Raf-1 (WCL)
45 kDa
75 kDa
β-actin (WCL)
45 kDa
Raf-1 (WCL)
ERK1/2 (WCL)

## Slide 28
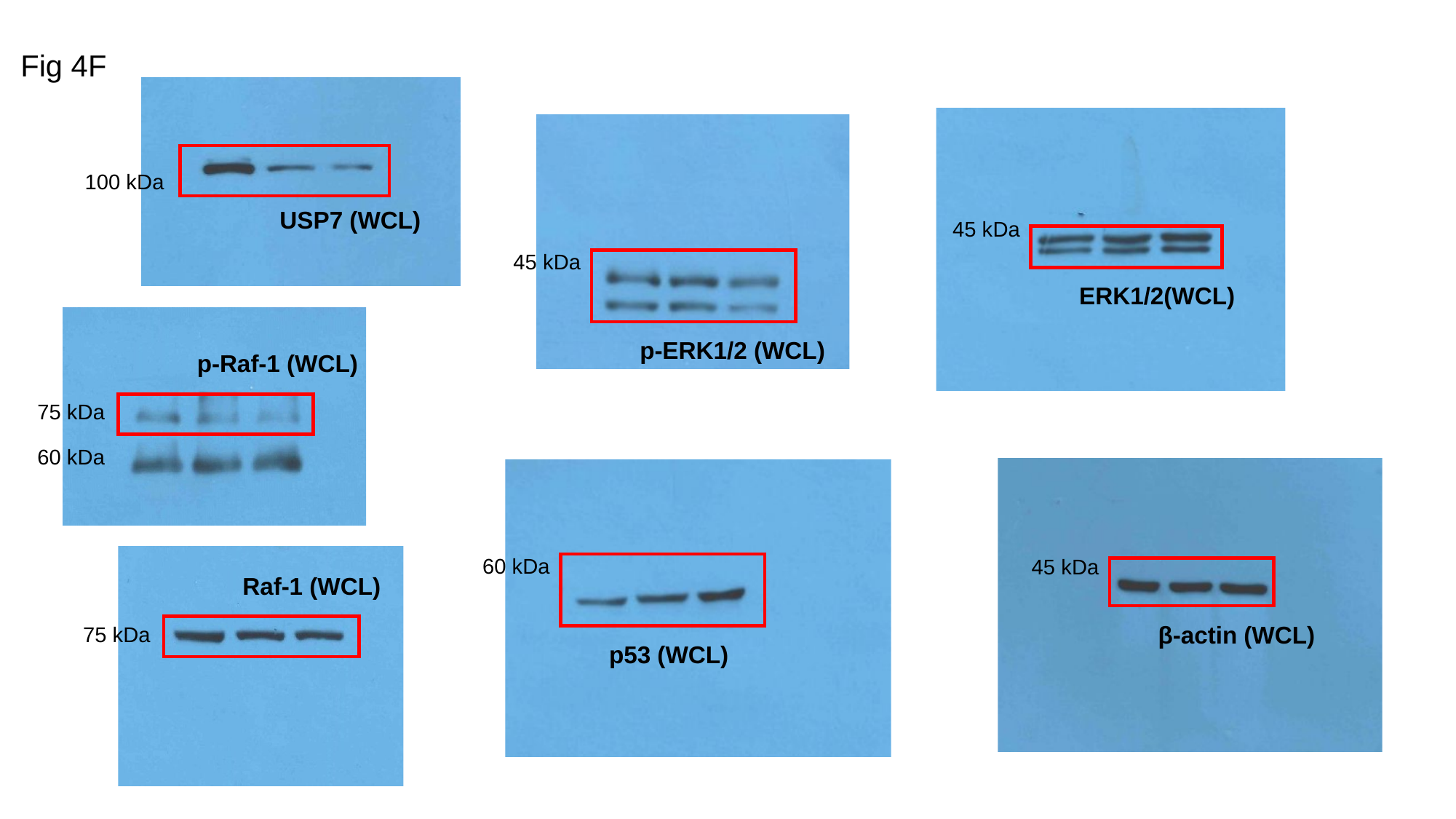

Fig 4F
100 kDa
USP7 (WCL)
45 kDa
45 kDa
ERK1/2(WCL)
p-ERK1/2 (WCL)
p-Raf-1 (WCL)
75 kDa
60 kDa
60 kDa
45 kDa
Raf-1 (WCL)
β-actin (WCL)
75 kDa
p53 (WCL)

## Slide 29
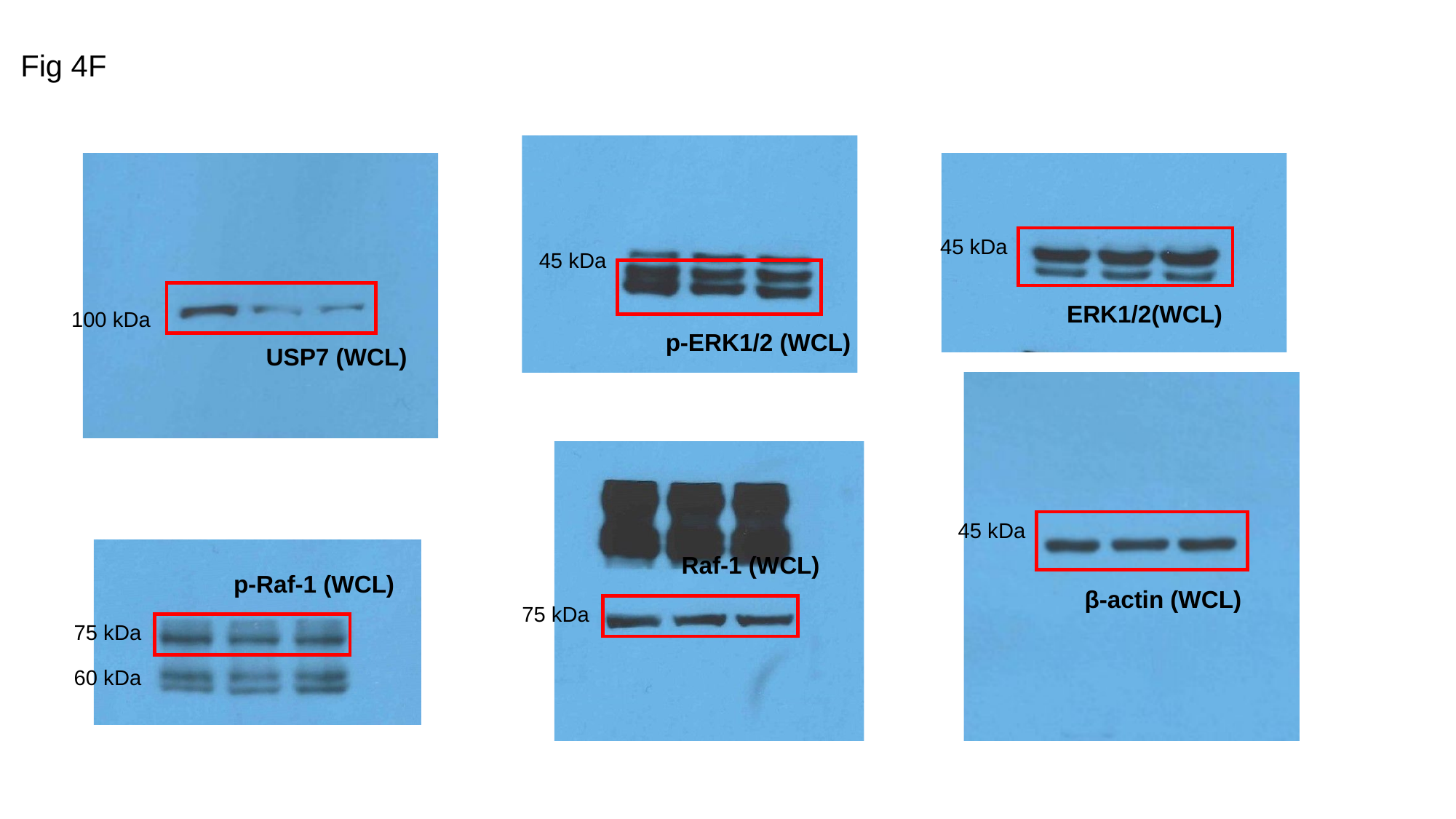

Fig 4F
45 kDa
45 kDa
ERK1/2(WCL)
100 kDa
p-ERK1/2 (WCL)
USP7 (WCL)
45 kDa
Raf-1 (WCL)
p-Raf-1 (WCL)
β-actin (WCL)
75 kDa
75 kDa
60 kDa

## Slide 30
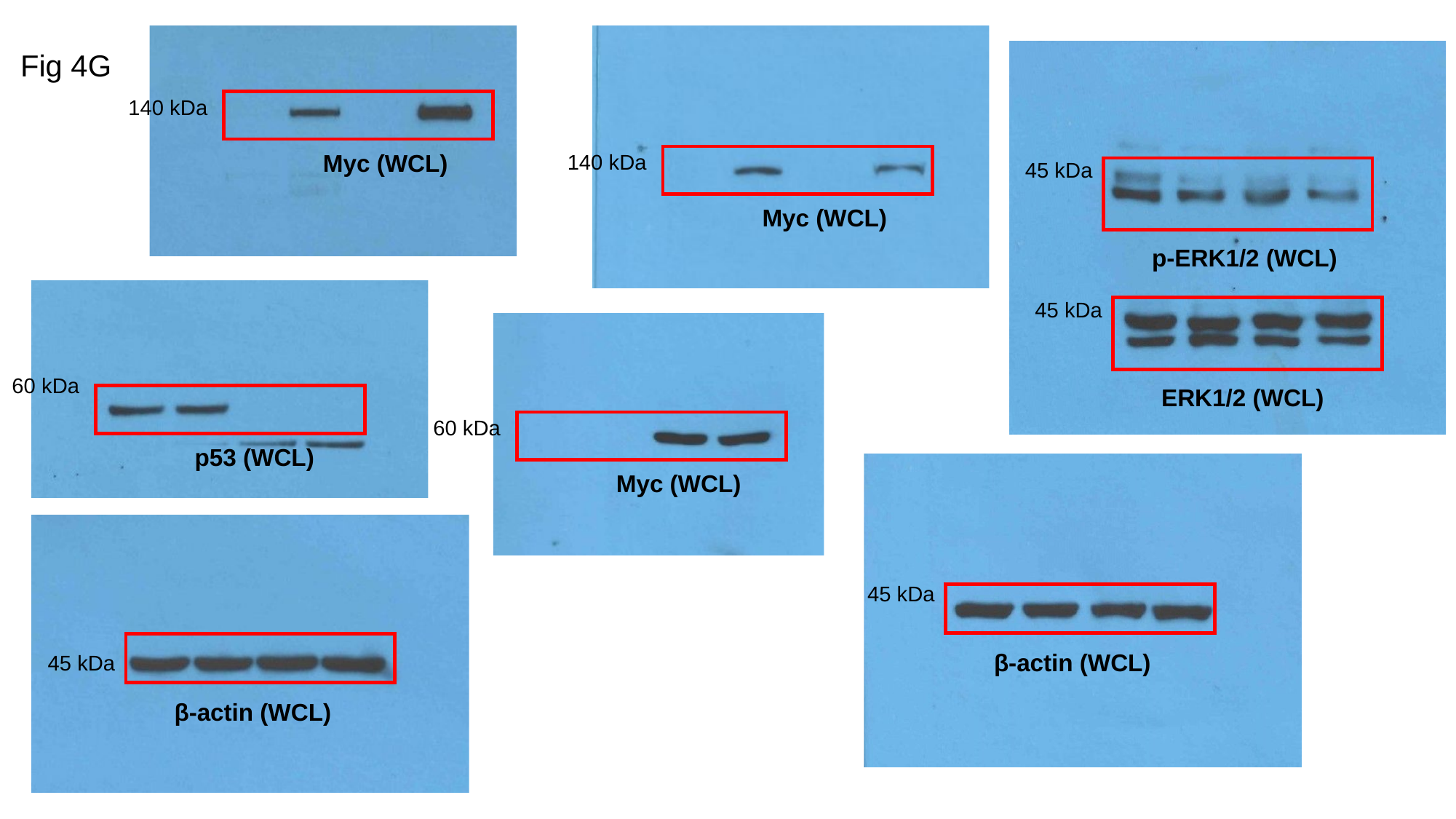

Fig 4G
140 kDa
Myc (WCL)
140 kDa
45 kDa
Myc (WCL)
p-ERK1/2 (WCL)
45 kDa
60 kDa
ERK1/2 (WCL)
60 kDa
p53 (WCL)
Myc (WCL)
45 kDa
β-actin (WCL)
45 kDa
β-actin (WCL)

## Slide 31
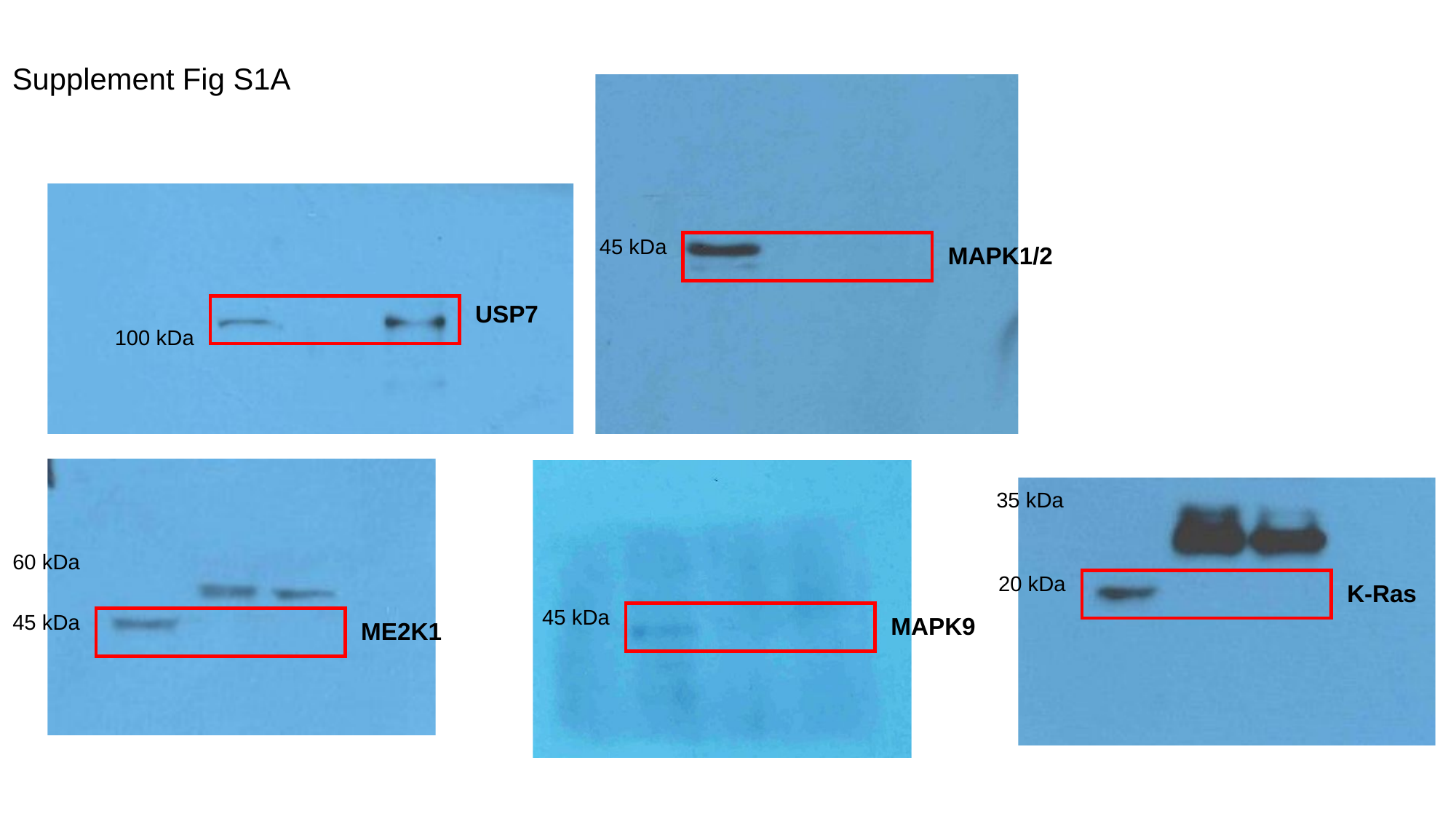

Supplement Fig S1A
45 kDa
MAPK1/2
USP7
100 kDa
35 kDa
60 kDa
20 kDa
K-Ras
45 kDa
45 kDa
MAPK9
ME2K1

## Slide 32
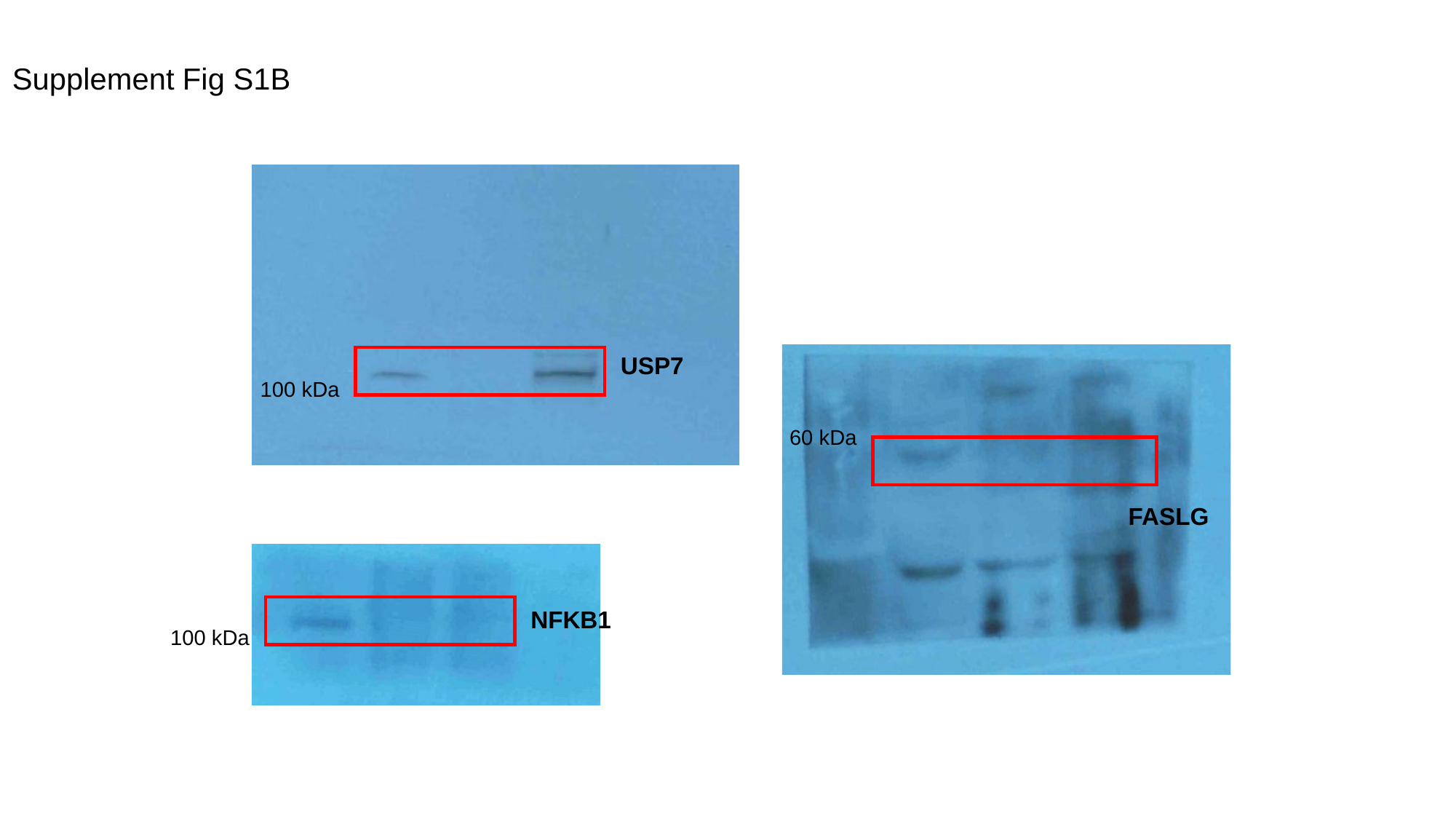

Supplement Fig S1B
USP7
100 kDa
60 kDa
FASLG
NFKB1
100 kDa

## Slide 33
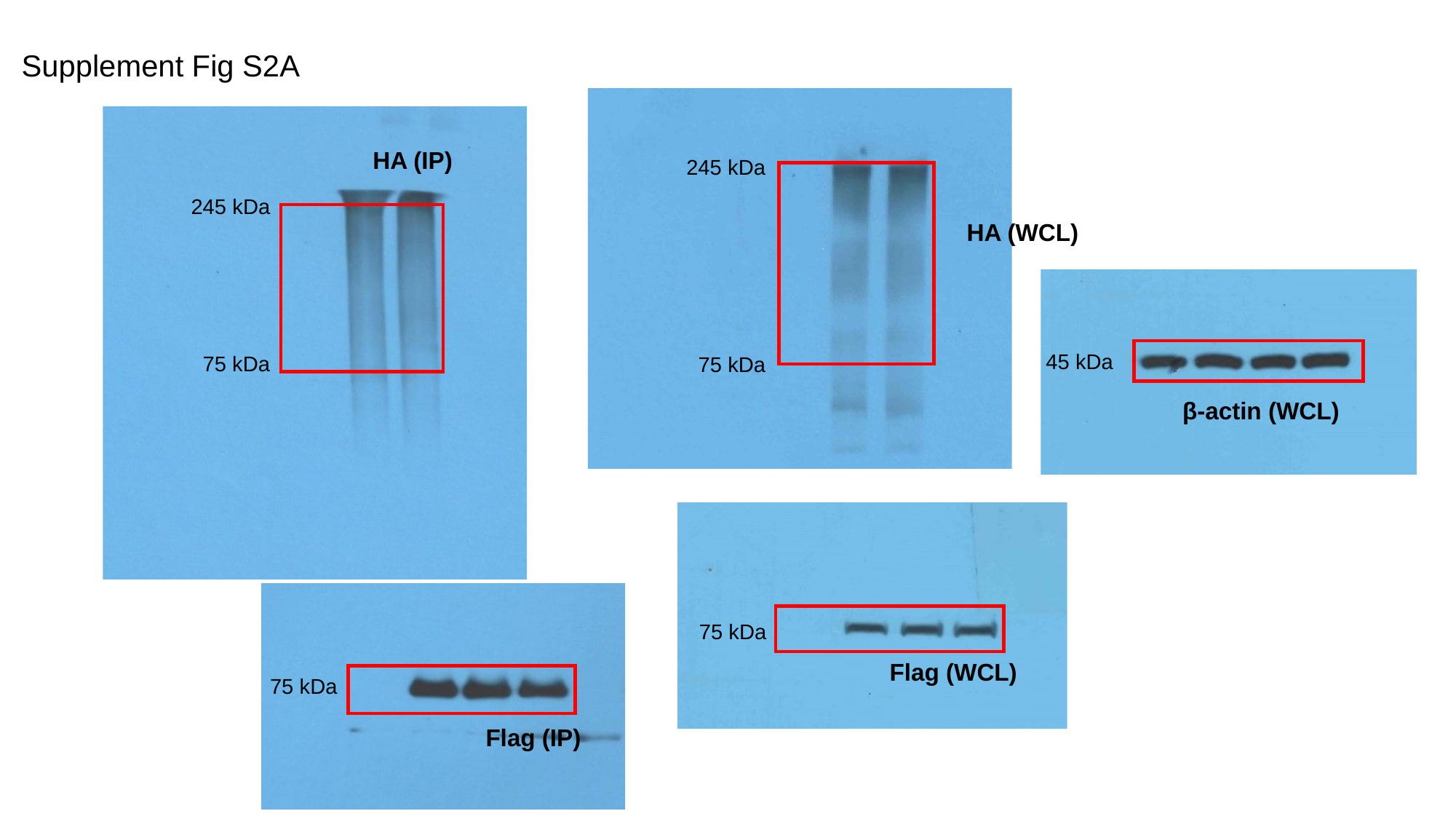

Supplement Fig S2A
HA (IP)
245 kDa
245 kDa
HA (WCL)
45 kDa
75 kDa
75 kDa
β-actin (WCL)
75 kDa
Flag (WCL)
75 kDa
Flag (IP)

## Slide 34
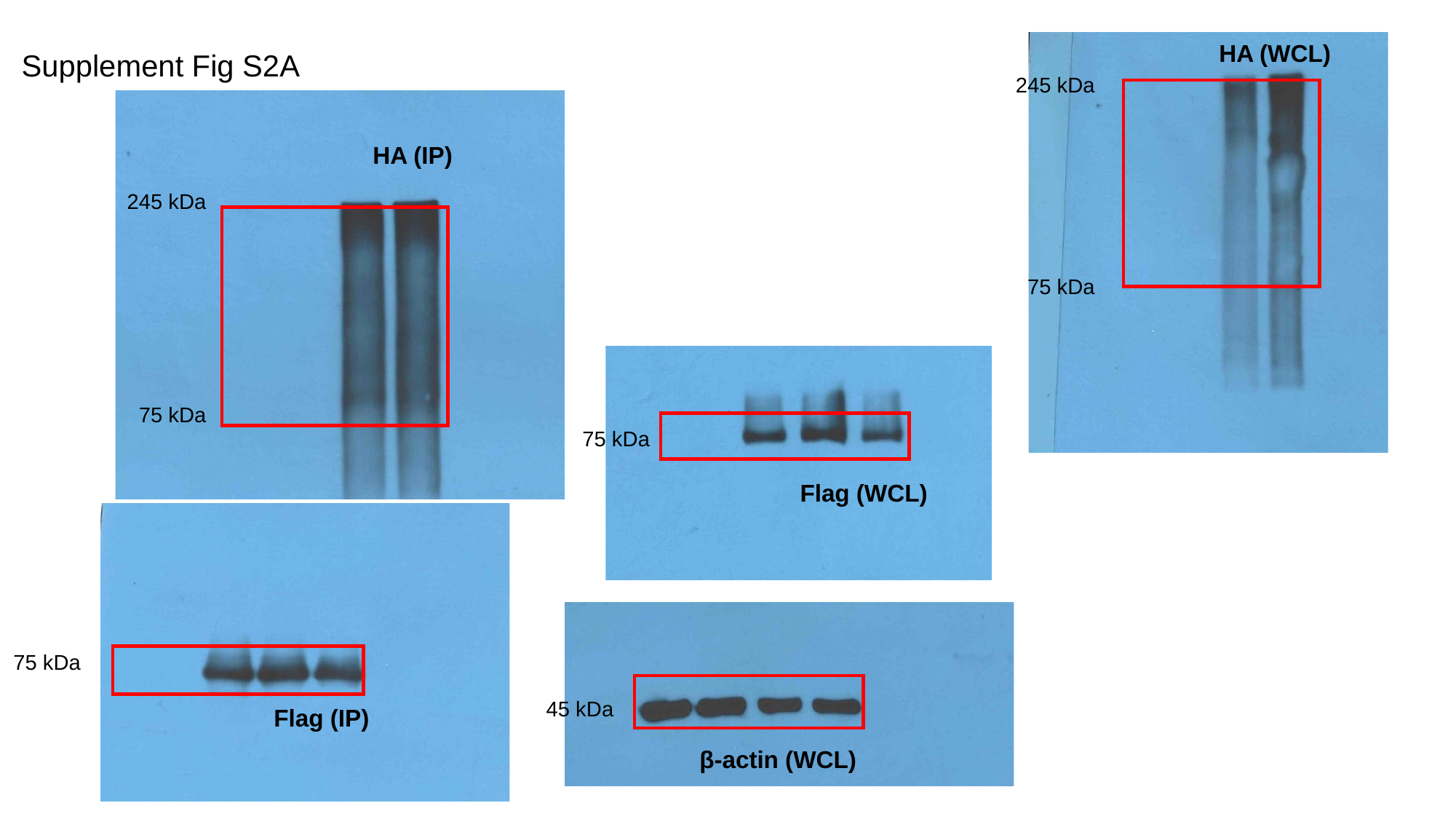

HA (WCL)
Supplement Fig S2A
245 kDa
HA (IP)
245 kDa
75 kDa
75 kDa
75 kDa
Flag (WCL)
75 kDa
45 kDa
Flag (IP)
β-actin (WCL)

## Slide 35
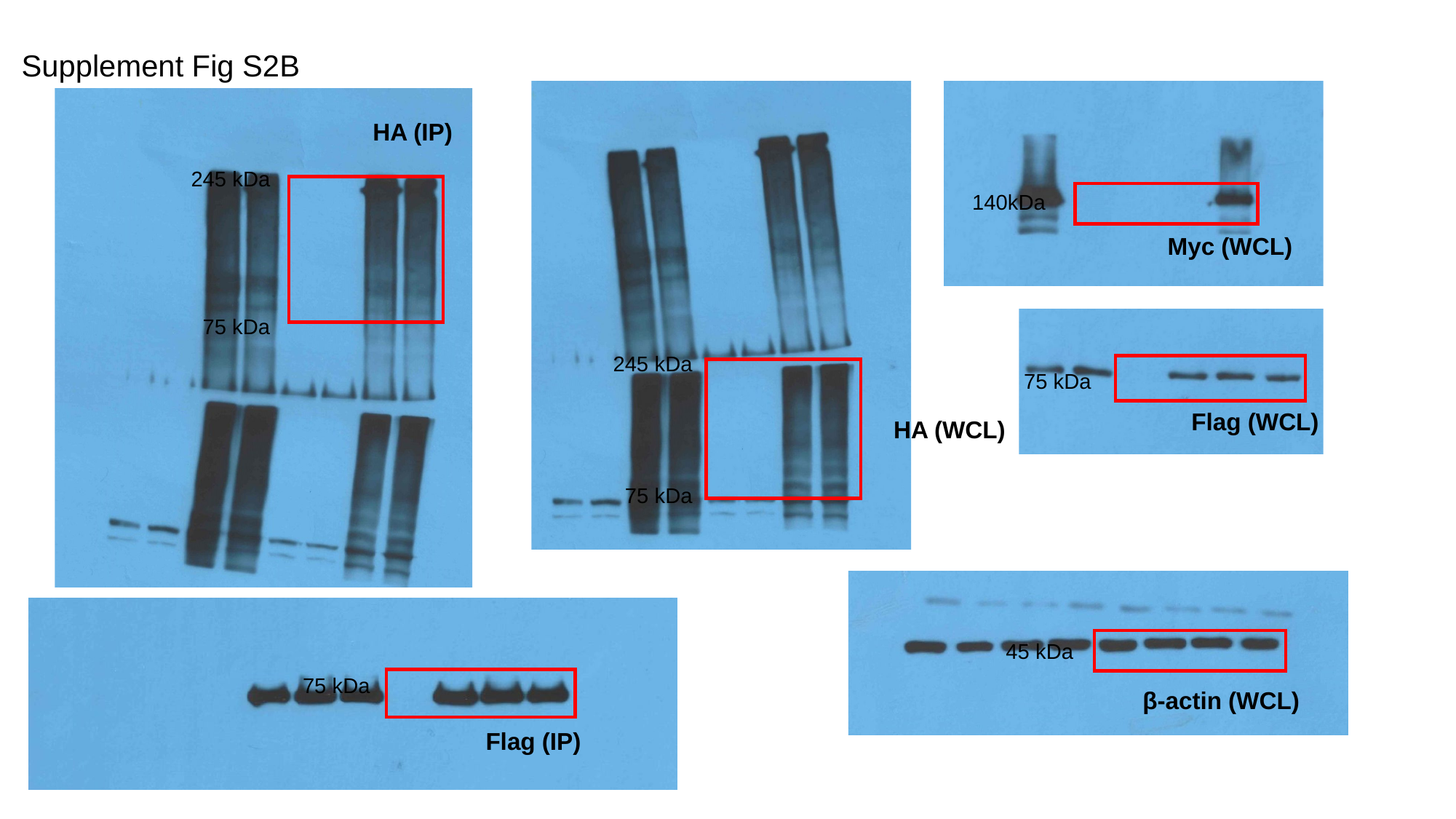

Supplement Fig S2B
HA (IP)
245 kDa
140kDa
Myc (WCL)
75 kDa
245 kDa
75 kDa
Flag (WCL)
HA (WCL)
75 kDa
45 kDa
75 kDa
β-actin (WCL)
Flag (IP)

## Slide 36
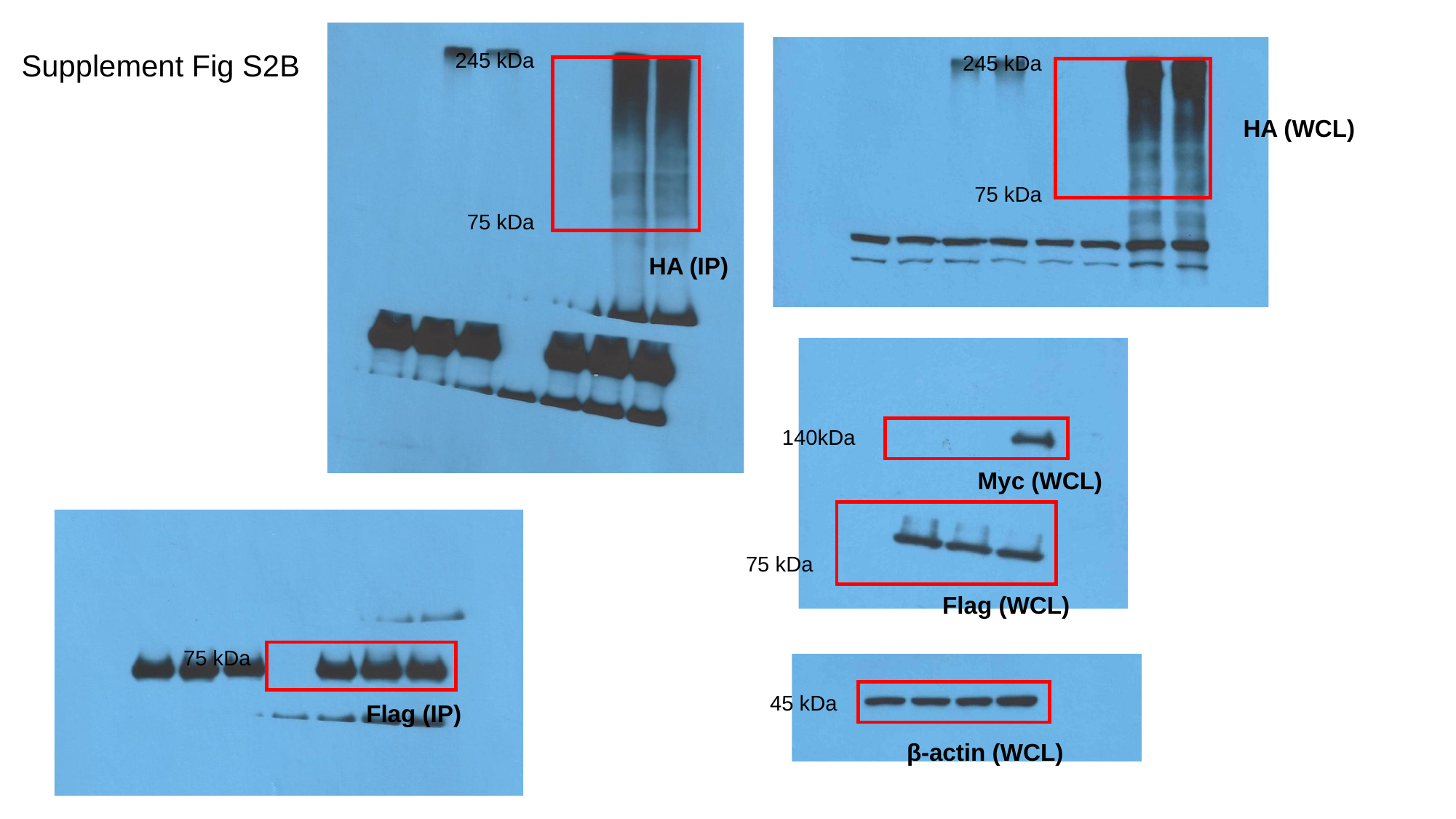

245 kDa
75 kDa
HA (IP)
Supplement Fig S2B
245 kDa
HA (WCL)
75 kDa
140kDa
Myc (WCL)
75 kDa
Flag (WCL)
75 kDa
45 kDa
Flag (IP)
β-actin (WCL)

## Slide 37
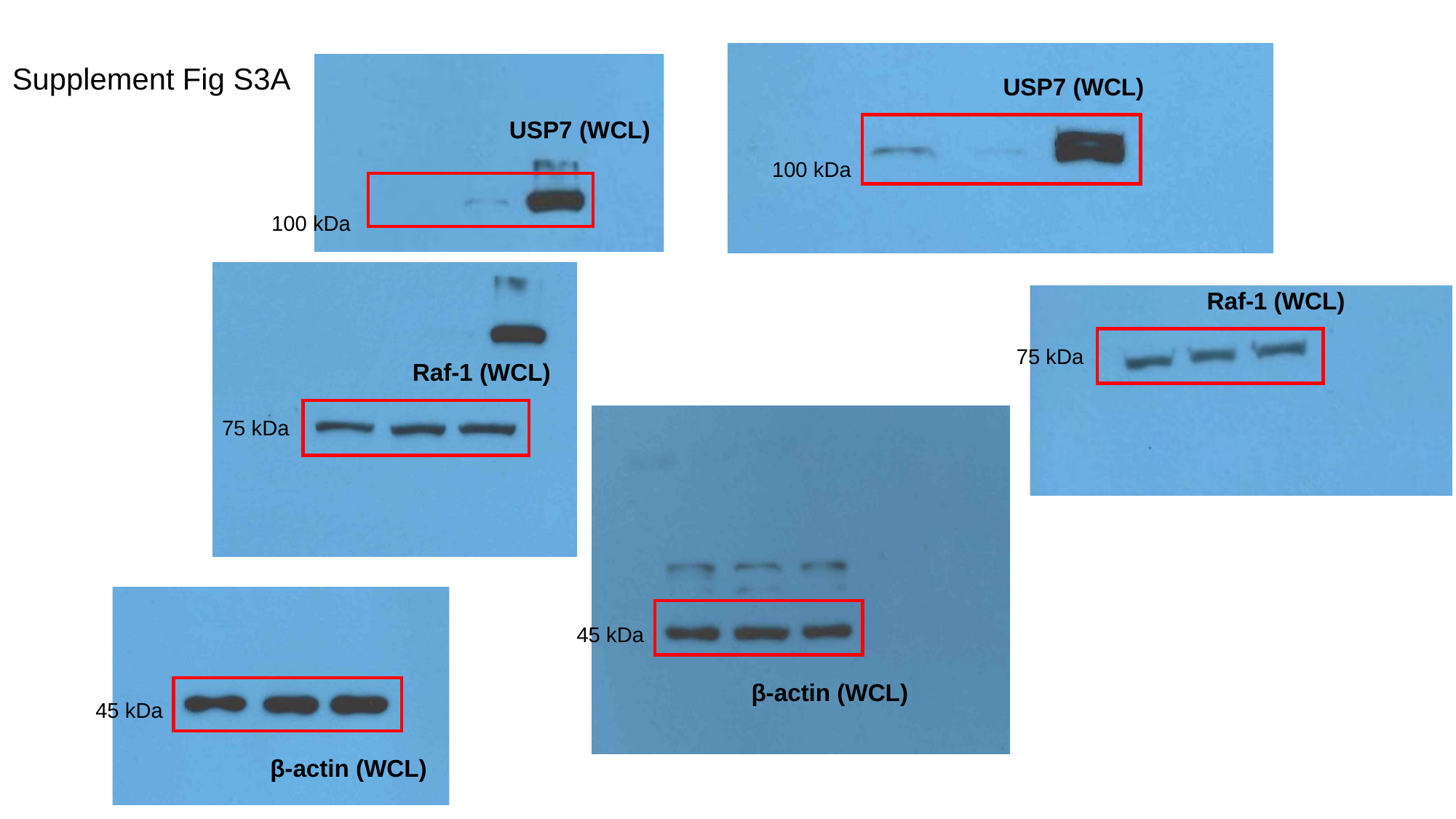

Supplement Fig S3A
USP7 (WCL)
USP7 (WCL)
100 kDa
100 kDa
Raf-1 (WCL)
75 kDa
Raf-1 (WCL)
75 kDa
45 kDa
β-actin (WCL)
45 kDa
β-actin (WCL)

## Slide 38
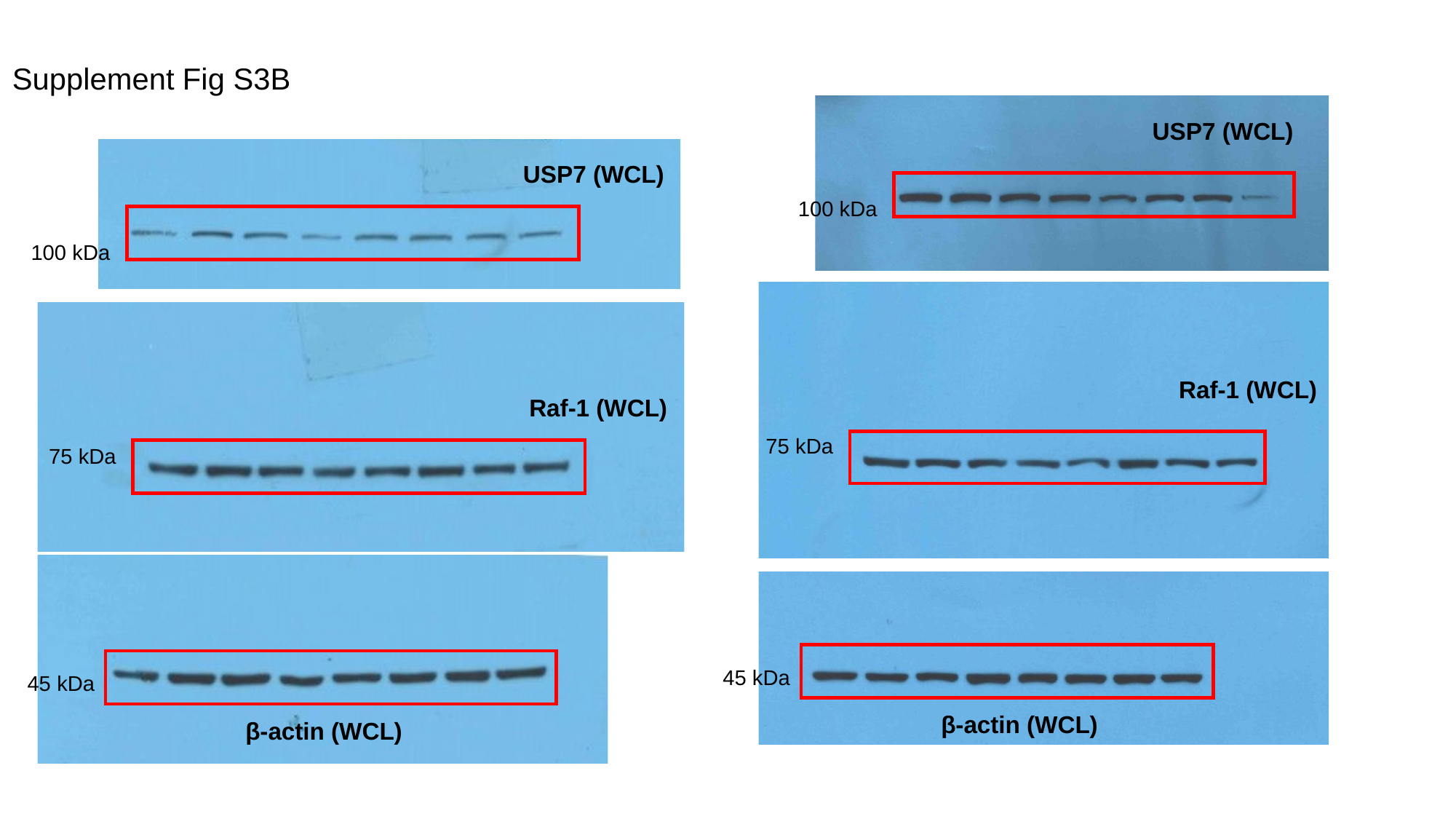

Supplement Fig S3B
USP7 (WCL)
USP7 (WCL)
100 kDa
100 kDa
Raf-1 (WCL)
Raf-1 (WCL)
75 kDa
75 kDa
45 kDa
45 kDa
β-actin (WCL)
β-actin (WCL)

## Slide 39
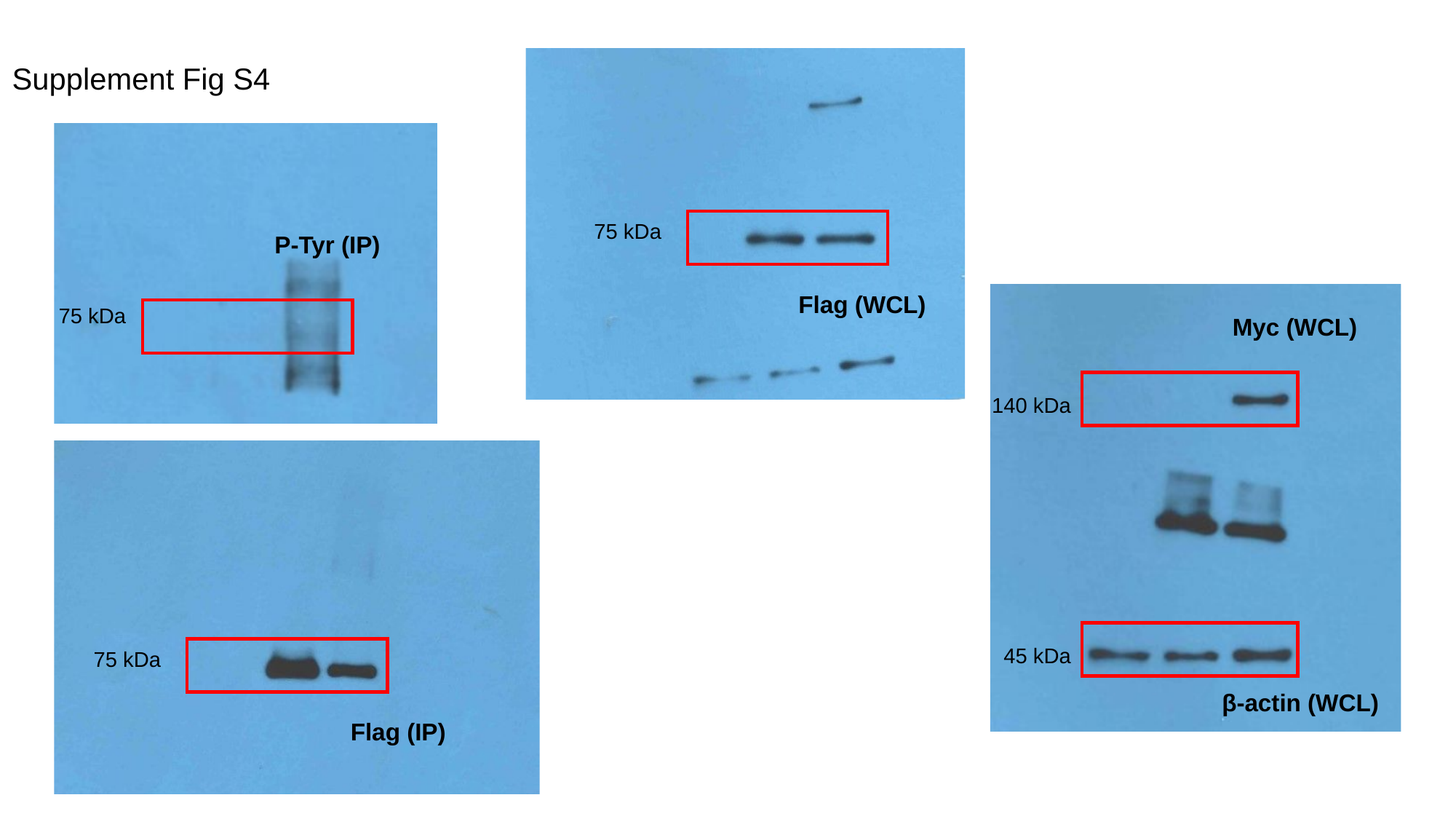

Supplement Fig S4
75 kDa
P-Tyr (IP)
Flag (WCL)
75 kDa
Myc (WCL)
140 kDa
45 kDa
75 kDa
β-actin (WCL)
Flag (IP)
